# VS411 Reduced Immune Activation and HIV-1 RNA Levels in 28 Days: Randomized Proof-of-Concept Study for AntiViral-HyperActivation Limiting Therapeutics

**DOI:** 10.1371/journal.pone.0047485

**Published:** 2012-10-19

**Authors:** Franco Lori, Davide De Forni, Elly Katabira, Denis Baev, Renato Maserati, Sandra A. Calarota, Pedro Cahn, Marco Testori, Aza Rakhmanova, Michael R. Stevens

**Affiliations:** 1 ViroStatics srl, Alghero, Italy; 2 Makerere Medical School, Kampala, Uganda; 3 Infectious Diseases Unit, Fondazione Istituto di Ricovero e Cura a Carattere Scientifico Policlinico San Matteo, Pavia, Italy; 4 Fundación Huesped, Buenos Aires, Argentina; 5 Department of Infectious Diseases, St. Petersburg Health Department, St. Petersburg, Russia; 6 ViroStatics srl, Princeton, New Jersey, United States of America; Rush University, United States of America

## Abstract

**Background:**

A new class of antiretrovirals, AntiViral-HyperActivation Limiting Therapeutics (AV-HALTs), has been proposed as a disease-modifying therapy to both reduce Human Immunodeficiency Virus Type 1 (HIV-1) RNA levels and the excessive immune activation now recognized as the major driver of not only the continual loss of CD4^+^ T cells and progression to Acquired Immunodeficiency Syndrome (AIDS), but also of the emergence of both AIDS-defining and non-AIDS events that negatively impact upon morbidity and mortality despite successful (*ie, fully suppressive*) therapy. VS411, the first-in-class AV-HALT, combined low-dose, slow-release didanosine with low-dose hydroxycarbamide to accomplish both objectives with a favorable toxicity profile during short-term administration. Five dose combinations were administered as VS411 to test the AV-HALT Proof-of-Concept in HIV-1-infected subjects.

**Methods:**

Multinational, double-blind, 28-day Phase 2a dose-ranging Proof-of-Concept study of antiviral activity, immunological parameters, safety, and genotypic resistance in 58 evaluable antiretroviral-naïve HIV-1-infected adults. Randomization and allocation to study arms were carried out by a central computer system. Results were analyzed by ANOVA, Kruskal-Wallis, ANCOVA, and two-tailed paired *t* tests.

**Results:**

VS411 was well-tolerated, produced significant reductions of HIV-1 RNA levels, increased CD4^+^ T cell counts, and led to significant, rapid, unprecedented reductions of immune activation markers after 28 days despite incomplete viral suppression and without inhibiting HIV-1-specific immune responses. The didanosine 200 mg/HC 900 mg once-daily formulation demonstrated the greatest antiviral efficacy (HIV-1 RNA: −1.47 log_10_ copies/mL; CD4^+^ T cell count: +135 cells/mm^3^) and fewest adverse events.

**Conclusions:**

VS411 successfully established the Proof-of-Concept that AV-HALTs can combine antiviral efficacy with rapid, potentially beneficial reductions in the excessive immune system activation associated with HIV-1 disease. Rapid reductions in markers of immune system hyperactivation and cellular proliferation were obtained despite the fact that VS411 did not attain maximal suppression of HIV RNA, suggesting this effect was due to the HALT component.

**Trial Registration:**

ITEudraCT 2007-002460-98

## Introduction

During viral infections, in particular with Human Immunodeficiency Virus Type 1 (HIV-1) infection, if the pathogen is not cleared (ie, chronic disease) a well-documented hyperactivation of the immune system occurs [1], [2], [3], [4], leading to a continuous cycle of increased T cell hyperproliferation and, ultimately, systemic inflammation. In HIV disease, however, chronic immune stimulation due to persistent HIV-1 replication, microbial translocation and other factors results in the eventual exhaustion of the immune system, accelerated senescence and, consequently, the onset of the acquired immunodeficiency syndrome (AIDS) despite maximally suppressive antiretroviral therapy (ART) regimens [5], [6], [7], [8].

With more than 20 antiretroviral medications available in six major classes [9] the use of multi-drug regimens has resulted in substantial reductions in progression to AIDS, opportunistic infections, hospitalizations, and deaths [10]. Current anti-HIV therapy can significantly reduce viral replication; however, despite achieving “undetectable” levels of circulating HIV RNA (ie, less than 50 copies/mL), immune system hyperactivation, cellular hyperproliferation and inflammation do not return to normal levels [2], [11], [12]. It is this incomplete deactivation of the immune system – and not the virus itself – that is now considered the primary driver of a growing number of “non-AIDS defining events” including cardiovascular disease, liver disease, kidney disease, bone loss, increased rates of cancer, and accelerated aging even in individuals for whom today’s anti-HIV combinations have reduced the HIV virus to undetectable levels in their blood [13], [14], [15], [16], [17].

There is a growing recognition that successful long-term therapy for the treatment of HIV-1 infection should be disease modifying, not only reducing replicating virus but also directly diminishing the excessive chronic activation of the immune system [2], [4], [18], [19], [20], [21]. In order to address this unmet need, researchers at ViroStatics have developed an innovative core platform of antiviral-immune protective compounds called AntiViral-HyperActivation Limiting Therapeutics (AV-HALTs) designed to reduce both HIV-1 RNA levels and excessive chronic immune system hyperactivation. To accelerate drug development and establish the AV-HALT Proof-of-Concept in the treatment of HIV-1 in man, two available generic drugs, a direct-acting antiviral (didanosine) and a hyperactivation-limiting agent (hydroxycarbamide [HC]) were combined in a fixed-dose-combination (FDC) capsule known as VS411. Individually, these two agents have been employed in the clinical setting for many years [22], [23].

By targeting a human enzyme, ribonucleotide reductase, rather than a viral one, the HC component of VS411 renders the intracellular milieu hostile to the HIV virus while also generating synergistic antiviral activity in combination with didanosine [24], [25], [26], [27], [28]. As HIV-1 requires actively dividing T cells to replicate [29], [30], HC can also reduce HIV-1 replication by limiting T cell proliferation required for maximal HIV-1 replication and increasing the levels of CC (or β) chemokines, the endogenous ligands of the HIV-1 co-receptor CCR5 (CC chemokine receptor type 5), theoretically hampering HIV-1 entry into host T cells [31].

Didanosine and HC have been experimentally combined in several HIV studies [32], [33], [34], [35], [36], [37], [38], mainly to test their antiviral (AV) and not their HALT activity. In this study we extensively measured, for the first time, the antiproliferative and anti-hyperactivation properties of this combination.

The dosages of the two drugs historically used to treat HIV-1 infection are now recognized as having been too high, generating immunosuppressive effects and toxicities that were further exacerbated by the frequent addition of a second nucleoside analogue, stavudine [39], [40], [41]. Since the side effects were largely dose dependent, we decided to investigate whether lower doses of both didanosine and HC could maintain antiviral efficacy while reducing toxicity during short-term (28 day) administration.

The five VS411 FDC formulations used in this dose-ranging study were developed using proprietary slow release, enterically-coated 100 mg didanosine tablets designed to modify the drug’s release profile, and thereby the didanosine plasma concentration-time profile, to further reduce potential toxicity. These tablets were combined with HC 150 mg tablets designed to duplicate the previous plasma concentration-time profile of HC [39]. The use of individual tablets of didanosine and HC allowed differing numbers of didanosine and HC tablets, as well as placebos for both, to be placed into blinded capsules for each cohort of the five-arm dose-ranging study.

We present here the results from a randomized Phase 2a study of VS411 devised to test the Proof-of-Concept that an AV-HALT can both inhibit viral replication and directly reduce markers of excessive immune system activation and cellular proliferation. After 28 days of therapy, VS411 was seen to safely provide both the AV and HALT activities required of the new AV-HALT drug class.

## Materials and Methods

### Study Design

This was a randomized, five-arm, double-blind Phase 2a multi-center study in which HIV-1-infected subjects naïve to antiviral therapy were treated with one of five distinct oral dose-dose combinations of VS411 for 28 days from Day 1 (baseline) to Day 29. Recruitment began in March of 2008. The first subject was dosed on May 12, 2008 and the trial concluded with the last subject’s final follow-up visit performed on January 12^th^, 2009. The protocol for this trial and supporting CONSORT checklist are available as supporting information; see Checklist S1 and Protocol S1.

The study protocol was approved by the following local or national institutional review boards for each of the participating sites: Fundación Huesped, Buenos Aires, Argentina (ANMAT [Administración Nacional de Medicamentos, Alimentos y Tecnología Médica]); Makerere University, Kampala, Uganda (UNCST [Uganda National Council for Science and Technology]); Centro de Asistencia e Investigación Clinica en Inmunocomprometidos (CAICI), Rosario, Argentina (ANMAT); Hospital General de Agudos Teodoro Alvarez, Buenos Aires, Argentina (ANMAT); Centro de Estudio y Tratamiento Infectológico, Buenos Aires, Argentina (ANMAT); Fondazione IRCCS Policlinico San Matteo, Pavia, Italy (Comitato di Bioetica della Fondazione IRCCS Policlinico San Matteo di Pavia); Azienda Ospedaliera di Parma, Parma, Italy (Comitato Etico Unico per la Provincia di Parma); Center for Prophylaxis and Control of AIDS, St. Petersburg, Russia (Russian Federation Ministry of Health [Federal Service on Surveillance in Healthcare and Social Development]); and Infectious Diseases City Hospital, St. Petersburg, Russia (Russian Federation Ministry of Health [Federal Service on Surveillance in Healthcare and Social Development]).

The objectives of this study were to characterize five different dose combinations of hydroxycarbamide plus didanosine in the form of VS411 with respect to:

1. HIV-1 antiviral activity;
2. Immunological parameters;
3. Safety/tolerability;
4. Evaluation of genotypic resistance to nucleoside/nucleotide analogues at baseline and after four weeks of treatment
5. Pharmacokinetics;
6. Pharmacokinetic and pharmacodynamic relationships; and
7. Quantification of intracellular ddATP/dATP.

This study report will address those factors associated with the use of VS411 as an AV-HALT – factors 1 through 4, above.

A sample size of 12 subjects per arm (total N = 60) was chosen for this study. This number of subjects is considered adequate for exploratory pharmacokinetic and first-in-man trials in which the lowest possible volunteer numbers are selected for safety reasons while the outcomes provide sufficient guidance for future development steps. IATEC Data Management performed the randomization centrally. Once the patient was considered eligible for the study and provided informed consent, based on the outcome of screening at the site, he/she was randomly assigned in a 1∶1∶1∶1∶1 ratio to one of the five treatment groups:

1. Didanosine 200 mg QD plus Hydroxyurea 300 mg QD
2. Didanosine 200 mg QD plus Hydroxyurea 600 mg QD
3. Didanosine 200 mg QD plus Hydroxyurea 900 mg QD
4. Didanosine 400 mg QD plus Hydroxyurea 300 mg QD
5. Didanosine 400 mg QD plus Hydroxyurea 600 mg QD.

Study arms 1, 2 and 3 contained one-half of the 400 mg daily didanosine dose recommended for those weighing 60 kg or more. Study arms 4 and 5 contained the full daily didanosine dose recommended for those weighing 60 kg or more. In order to allow for randomization across all five study arms, only those individuals weighing 60 kilograms or more were included in the study.

A randomization schedule containing the randomization numbers was used. The randomization number was a unique number composed of an “R” followed by three digits that represented the allocated treatment arm. The distribution of randomization numbers is listed in Table 1.

**Table 1:** Distribution of medication “block-wise” to the study counties.

Country	Uganda	Russia	Argentina	Italy	Spare Kits	Total
**Estimated subjects**	35^a^	12 to 20	8 to 24	4 to 12		
**Random blocks of 5**	8	2	2	1	2	15 blocks
**Identical blocks of 5**	0	3	3	3	0	9 blocks
**Total blocks**	8	5	5	4	2	24 blocks
**Total kits**	40	25	25	20	10	120 kits

a A maximum of 35 randomized subjects was set for Uganda.

A treatment block – whether it was random or identical – each contained a randomized mixture of the five study treatment arms. Random blocks were used for the first randomizations in each country and were preferably filled (ie, five subjects assigned within each block) in order to minimize the differences in distribution of treatment groups among the different countries.

Study clinicians and participants were blinded to the identity of the study medication. VS411 was administered daily as three capsules that, when taken together, yielded five distinct dosing combinations of hydroxycarbamide and didanosine. To ensure blinding, each individual hard gelatin capsule contained 4 tablets consisting of enterically-coated didanosine 100 mg or matching placebo or HC 150 mg or matching placebo as listed in Table 2. To achieve the five dosing arms, each subject received individual bottles containing a daily dose of three capsules as represented in Table 3. The VS411 study medication was prepared, packed and labeled by Aptuit Limited (Edinburgh, Scotland, UK) in accordance with Good Manufacturing Practices and applicable local laws and regulations. Subjects were instructed to take VS411 orally on an empty stomach in the morning, without food, and at least 2 hours before breakfast.

**Table 2:** Components of the VS411 capsules.

Capsule Type	Components	Resulting Doses
**VS411-A**	2 ddI^a^ 100 mg EC^b^ tablets/2 HC^c^ 150 mg tablets	ddI: 200 mg/HC: 300 mg
**VS411-B**	2 ddI placebo EC tablets/2 HC 150 mg tablets	ddI: 0 mg/HC: 300 mg
**VS411-C**	2 ddI 100 mg EC tablets/2 HC placebo tablets	ddI: 200 mg/HC: 0 mg
**VS411-D**	2 ddI placebo EC tablets/2 HC placebo tablets	ddI: 0 mg/HC: 0 mg

a ddI  =  didanosine.

b EC  =  Enterically coated.

c HC  =  hydroxycarbamide.

**Table 3:** Number of VS411 capsules required for each study arm.

Dosing Arm	VS411-A	VS411-B	VS411-C	VS411-D
**ddI** a **400 mg/HC** b **600 mg**	2			1
**ddI 200 mg/HC 600 mg**	1	1		1
**ddI 200 mg/HC 300 mg**	1			2
**ddI 400 mg/HC 300 mg**	1		1	1
**ddI 200 mg/HC 900 mg**	1	2		

a ddI  =  didanosine.

b HC  =  hydroxycarbamide.

Study endpoints were: changes from baseline in the log-transformed plasma HIV-1 RNA concentrations; changes in CD4^+^ T cell counts; incidence and severity of adverse events; a comparative evaluation of genotypic resistance to nucleoside/nucleotide analogues at baseline and after 28 days of VS411 treatment; a comparison of a panel of pre- and post-treatment markers of immune system activation and cellular proliferation in a representative 32-subject subset; and an analysis of changes in HIV-1-specific immune responses in a representative 22-subject subset.

### Study Population

This was a multinational study enrolling subjects in nine centers in four countries selected to mirror the global pandemic: Italy, Russia, Uganda, and Argentina. The study began upon receiving written regulatory approval from the national authorities and local ethical committees operating according to The International Conference on Harmonisation of Technical Requirements for Registration of Pharmaceuticals for Human Use (ICH) and the World Health Organization’s Guidelines for good clinical (GCP) for trials on pharmaceutical products. All subjects provided written informed consent.

Eligible subjects were chronically infected adult males or non-lactating females with HIV-1 who were HIV-1 antiretroviral treatment-naïve with plasma HIV-1 RNA levels greater than 5,000 copies/mL, CD4^+^ T-cell counts greater than 250 cells/mm^3^, and falling under CDC AIDS Surveillance Case Definition classification stage A or B. To allow for the randomized dosing of didanosine at 400 mg per day, subjects had to weight 60 kilograms or more. Sexually active subjects were required to use adequate and reliable forms of contraception during the study, starting at least one month prior to study drug administration for females and continuing for 30 days after discontinuation of study medication.

Exclusion criteria included HIV-2 co-infection; primary (acute) HIV-1 infection; less than 60 kg body weight; a series of clinically relevant laboratory abnormalities indicative of anemia, leucopenia, elevated triglycerides, elevated liver transaminases, elevated bilirubin, elevated amylase, elevated lipase, or decreased estimated creatinine clearance; use of co-medication with a known clinically significant pharmacological interaction with one or more of the study drugs; active alcohol or drug abuse (methadone use was allowed); use of proton pump inhibitors; anticipated non-compliance with the protocol; anticipated need to start HAART within the study period; presence of a newly (within 30 days) diagnosed HIV-related opportunistic infection or condition requiring acute therapy at the time of enrolment; having received any investigational drug 30 days prior to the start of the study; chronic active viral hepatitis B or hepatitis C, current or previous pancreatitis; pregnancy; history of, or currently receiving, interferon therapy; presence of didanosine-associated resistance mutations; current or recent (within the prior three months) use of immunomodulatory agents (including vaccines); current or recent (within the prior three months) use of ribavarin; and known allergy to the active or inactive components of VS411.

### Study Monitoring, Data Collection, Analysis and Statistical Methods

The study was monitored by an independent CRO, IATEC, who also provided data management. Data were described by means and standard deviations or median and interquartile ranges. The changes from baseline in log-transformed plasma HIV-1 RNA levels and immunological parameters were analyzed by a repeated measurements procedure using a generalized linear model (PROC MIXED of SAS software, SAS version 9.1). The incidence of adverse events and laboratory abnormalities were tabulated and compared between study arms using the chi-square test. All tests of hypotheses were two-sided and used a 5% level of significance. The described analyses were performed on a modified Intent-to-treat (mITT) population (defined as all randomized subjects who received at least one dose of study medication [N = 58]), the On-Treatment (OT) population (defined as all subjects who took 75% or more of the planned doses of study medication and complete the study without major protocol violations [N = 56]). Data presented in this paper reflect the mITT population.

Two sub-populations were also studied to document changes in immunological parameters. Pre- and post-treatment time points were selected for these analyses due to the short nature of the study (ie, 28 days). These analyses were intended to be conducted only on a subset of the total population due to the logistical complexity of adequately collecting, processing and shipping viable cells at multiple time points. Analyses of the panel of four biomarkers were performed upon viable cells obtained from 38 subjects from sites that had the capacity to properly collect, store, and ship viable cell samples per the protocol. An analysis of changes in HIV-1-specific immune responses was performed in a subset of 22 of those 38 subjects with a sufficient number of viable cells remaining after the biomarker analyses. Comparisons of each sub-population to the mITT population showed them to be statistically similar in terms of response to study therapy (data not shown) and baseline demographics. The baseline demographics for the two sub-studies are available as supporting information; see Sub-Study Demographics S1.

### HIV-1 RNA

Eurofins Medinet B.V. (Breda, The Netherlands) provided the Central Laboratory services. The plasma HIV-1 RNA levels were determined using the commercial “RT-PCR Cobas Ampliprep-Cobas Amplicor” (Roche, Indianapolis, IN) assay with a lower limit of quantification of 50 copies/mL.

### Genotypic Resistance

HIV Genosure (Lab Corp), an HIV genotyping assay, was employed to examine the viral genetic sequence to identify resistance-associated mutations. Both the reverse transcriptase and the protease genes were examined. Viral RNA was isolated from plasma samples at baseline and at Day 29 (after 28 days of VS411 treatment), reverse transcribed and the relevant genome segment amplified and sequenced using Applied Biosystems Incorporated technology. Subject-derived viral sequences were compared to wild type to identify specific mutations associated with current antivirals.

### Collection and Processing of Peripheral Blood Mononuclear Cells (PBMCs)

Selected sites were recruited to provide subject blood samples for the two immunology sub-studies – flow cytometry analysis of immune activation markers in 32 subjects and analysis of HIV-1-specific immune responses by ELISPOT assay in 22 subjects. At these sites, 30 mL of blood were collected on Visit 1 (pre-therapy) and Visit 7 (after 28 days of therapy) from each subject. Viable PBMCs were then obtained by Ficoll-Paque Plus density gradient separation, aliquoted in vials, frozen and stored in liquid nitrogen. Each study site was instructed in, and was responsible for, the separation, cryopreservation and storage of all samples collected until the material was sent via international courier to ViroStatics laboratories in Pavia, Italy where the analyses were performed.

The PBMCs were shipped using dry ice to avoid thawing of the material in transit and materials received by ViroStatics were immediately inspected to ensure thawing had not occurred. All laboratories involved used human materials sampled in the study only in accordance with the study protocol, the international Good Clinical Practice principles as stated in the ICH GCP guidelines, the EU Clinical Trial Directive, and other relevant legislative or regulatory requirements as was required by the site’s country.

### Flow Cytometry Analysis

PBMCs isolated from 32 individuals at baseline (Visit 1) and at Day 29 (Visit 7) were thawed and stained with AmCyan – anti-CD4 (Becton Dickinson Biosciences, San Jose, CA, USA), PacificBlue – anti-CD8 (Becton Dickinson Biosciences, San Jose, CA, USA), Alexa Fluor700– anti-CD3 (Becton Dickinson Biosciences, San Jose, CA, USA), APC – anti-HLA-DR (eBioscience, San Diego, CA, USA), PerCP-Cy5.5– anti-CD45RA (eBioscience, San Diego, CA, USA), APC-Cy7– anti-CD27 (BioLegend, San Diego, CA, USA), FITC – anti-PD1 (Becton Dickinson Biosciences, San Jose, CA, USA), ECD (PE-TexRed) – anti-CD69 (Beckman-Coulter, Brea, CA, USA), and PE-Cy5– anti-CD38 (Beckman-Coulter, Brea, CA, USA) for surface markers. For intracellular staining, samples were permeabilized with the BD Cytofix/Cytoperm™ Fixation/Permeabilization Solution Kit (Becton Dickinson Biosciences, San Jose, CA, USA) according to the manufacturer’s protocol. Cells were then stained with PE – anti-Ki-67 (Becton Dickinson Biosciences, San Jose, CA, USA). PE - IgG1- isotype control (Becton Dickinson Biosciences, San Jose, CA, USA) was employed. This subgroup of 32 subjects had baseline characteristics and response to treatment similar to the overall cohort (data not shown).

Samples were analyzed by using a BD LSR-II flow cytometer supplied with DiVa 6.1 acquisition and analysis software (Becton Dickinson Biosciences, San Jose, CA, USA). A minimum of 0.5 million events in the CD3^+^ T cell subset were acquired for all samples. Student’s and Wilcoxon non-parametric paired tests were used to evaluate the statistical significance of data obtained. *P* values <.05 were considered as significant.

### ELISPOT Assay

ELISPOT assays were carried out with PBMCs collected from a 22-subject subgroup. This subgroup had baseline characteristics and response to treatment similar to the overall cohort (data not shown). Peptides used in this study were obtained from the National Institutes of Health (NIH) AIDS Research and Reference Reagent Program, Division of AIDS, National Institute of Allergy and Infectious Diseases.

A human dual IFN-**γ**/IL-2 ELISPOT kit (Diaclone, Besançon Cedex, France) was used. PBMCs were plated in duplicate at 1×10^5^ cells per well. HIV-1 peptide pools were added and PHA (5 µg/mL; Sigma-Aldrich) was used as a positive control. Cells were resuspended in complete culture medium served as a negative control. Plates were read and the spots counted using an automated ELISPOT reader system (A-EL-Vis GmbH, Hannover, Germany). The mean number of single IFN-**γ**, single IL-2 and dual IFN-**γ**/IL-2 spots from duplicate wells was adjusted to 1×10^6^ PBMCs. The net single IFN-**γ**, single IL-2 and dual IFN-**γ**/IL-2 spots per million PBMCs were calculated by subtracting the number of sports formed in negative control medium wells from the number of spots formed in response to each peptide pool. Responses to the three Gag pools were summed to calculate total Gag response (denoted as Gag). Student’s t-test was used to assess differences in net spots/million PBMCs detected by ELISPOT assay. A value of *P*<.05 was considered statistically significant.

## Results

### Baseline Characteristics

**Figure 1:**
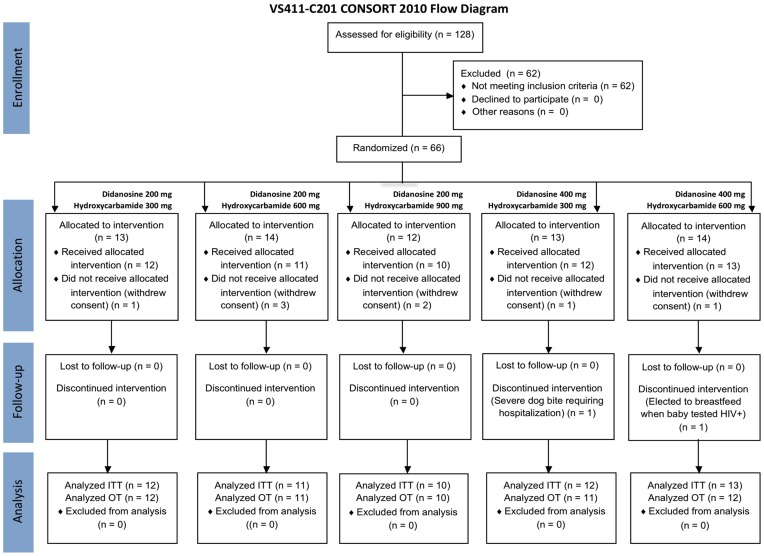
CONSORT Flow Diagram.

Eligible patients were chronically infected with HIV-1, at least 18 years old, weighed 60 kg or more, and HIV-1 antiretroviral treatment-naïve with plasma HIV-1 RNA levels >5,000 copies/mL and CD4^+^ T cell counts >250 cells/mm^3^. A total of 66 subjects were randomized into the study as seen in Figure 1. Eight randomized individuals withdrew consent prior to receiving study medication resulting in 58 subjects entering the trial and having received at least one dose of study medication (the modified Intent-to treat [mITT] population). Two subjects terminated their participation in the trial after receiving at least one dose of study medication – one following a severe dog bite, the other electing to breastfeed. Per protocol, these two subjects were not replaced, resulting in 56 subjects who completed the entire study on study medication. The results presented in this paper are those from the mITT analyses.

Subjects were well balanced between study arms and baseline characteristics are shown in Table 4. Fifty percent of the total population was female, 50% was Black, 43.1% was Caucasian and 6.9% was Hispanic. Median age was 33.0 years and median weight was 70.2 kg. Median Body Mass Index (BMI) was 25.1 kg/m^2^. Median CD4^+^ T cell count was 406.5 cells/mm^3^ and median HIV-1 RNA was 4.62 log_10_ copies/mL (44,668 copies/mL). Two subjects who entered the trial with protocol deviations, one despite having a CDC Classification of C while another had a thrombocyte count <100,000/mL at screening, were allowed to complete the study.

**Table 4:** Baseline characteristics.

	ddI^a^ 200 mgHC^b^ 300 mg	ddI 200 mg HC 600 mg	ddI 200 mg HC 900 mg	ddI 400 mg HC 300 mg	ddI 400 mg HC 600 mg	Total
**Subjects/Arm**	n = 12	n = 11	n = 10	n = 12	n = 13	**N = 58** c
**Age (years), median**	30.5	30.0	35.0	32.5	36.0	**33.0**
**Male, (%)**	7 (58.3)	4 (36.4)	5 (50.0)	5 (41.7)	8 (61.5)	**29 (50.0)**
**Female, (%)**	5 (41.7)	7 (63.6)	5 (50.0)	7 (58.3)	5 (38.5)	**29 (50.0)**
**Black, (%)**	5 (41.7)	6 (54.5)	6 (60.0)	6 (50.0)	6 (46.2)	**29 (50.0)**
**Caucasian, (%)**	5 (41.7)	5 (45.5)	4 (40.0)	5 (41.7)	6 (46.2)	**25 (43.1)**
**Hispanic, (%)**	2 (16.7)	0 (0.0)	0 (0.0)	1 (8.3)	1 (7.7)	**4 (6.9)**
**Weight (kg), median**	71.2	63.5	72.5	66.0	76.0	**70.2**
**Height (cm), median**	171.5	163.0	163.5	165.0	166.0	**166.0**
**BMI** d **(kg/m^2^), median**	25.3	24.1	26.0	24.2	27.0	**25.1**
**CD4^+^ T cells/mm^3^, median** **(range)**	455 (202–591)	468 (227–962)	407 (159–719)	349 (189–738)	456 (211–866)	**407 (159–962)**
**HIV-1 RNA log_10_ copies/mL,** **median (range)**	4.82 (3.53–5.15)	4.33 (3.62–5.04)	4.45 (3.75–5.20)	4.55 (3.35–4.96)	4.46 (3.88–5.67)	**4.62 (3.35–5.67)**

a ddI  =  didanosine.

b HC  =  hydroxycarbamide.

c modified ITT population.

d BMI  =  Body Mass Index.

### Safety

There were no deaths, serious adverse events (SAEs) or discontinuations due to study drug, nor were there major differences between arms in terms of clinical or laboratory safety. The number of subjects experiencing adverse events (AEs) is reported by system organ class and by study arm in Table 5. A total of 83 AEs were experienced by 36 of the 58 mITT subjects. Sixty-one of the 83 AEs were reported as “mild,” 20 as “moderate” and two as “severe.” The two “severe” AEs included one subject (in the didanosine 400 mg/HC 300 mg arm) who was severely bitten by a dog and discontinued from the trial. Another subject (in the didanosine 400 mg/HC 600 mg arm) experienced grade 3 neutropenia at Visit 5 (Day 8); however, the subject improved to grade 1 at Visit 6 (Day 15) with no change in therapy.

**Table 5:** Subjects with adverse events (AEs) by system organ class and treatment group.

MeDRA SOC^a^	ddI^b^ 200 mg HC^c^ 300 mg	ddI 200 mgHC 600 mg	ddI 200 mgHC 900 mg	ddI 400 mgHC 300 mg	ddI 400 mgHC 600 mg	Total
**Subjects/Arm**	n = 12	n = 11	n = 10	n = 12	n = 13	N = 58^d^
**Subjects with AE, (%)**	8 (66.7)	6 (54.5)	4 (40.0)	8 (66.7)	10 (76.9)	36 (62.1)
**Blood/Lymph, (%)**	4 (33.3)	1 (9.1)	2 (20.0)	2 (16.7)	4 (30.8)	13 (22.4)
**Gastrointestinal, (%)**	–	2 (18.2)	1 (10.0)	1 (8.3)	2 (15.4)	6 (10.3)
**General, (%)**	1 (8.3)	–	–	–	–	1 (1.7)
**Infections, (%)**	4 (33.3)	2 (18.2)	2 (20.0)	1 (8.3)	3 (23.1)	12 (20.7)
**Injury/Poisoning, (%)**	–	–	–	1 (8.3)	–	1 (1.7)
**Investigations, (%)**	2 (16.7)	3 (27.3)	2 (20.0)	6 (50.0)	4 (30.8)	17 (29.3)
**Musculoskeletal, (%)**	–	1 (9.1)	1 (10.0)	1 (8.3)	1 (7.7)	4 (6.9)
**Nervous System, (%)**	1 (8.3)	–	1 (10.0)	–	–	2 (3.4)
**Skin, (%)**	–	–	–	1 (8.3)	–	1 (1.7)

a SOC  =  System Organ Class.

b ddI  =  didanosine.

c HC  =  hydroxycarbamide.

d modified ITT population.

The total number of AEs was similar between subjects receiving full-dose didanosine (400 mg QD) and receiving half-dose didanosine (200 mg QD); however, due to study design the former group had only 25 subjects compared to the 33 subjects in the latter group. The arm with the highest reported percentage of subjects experiencing an AE was the didanosine 400 mg/HC 600 mg (76.9%) while the lowest percentage was reported for the didanosine 200 mg/HC 900 mg arm (40.0%).

The MeDRA System Organ Class with the highest number of reported adverse events was “Investigations” (consisting of abnormal laboratory values) with 17 of 58 subjects (29.3%) experiencing one or more events. The highest percentage occurred in the didanosine 400 mg/HC 300 mg arm (50.0%) while the lowest percentages were reported for the didanosine 200 mg/HC 900 mg (20.0%) and didanosine 200 mg/HC 300 mg (16.7%) arms. Within the entire cohort, the most commonly reported abnormalities were increases in blood bilirubin and lactic acid (each reported in four subjects, 6.9%) and increases in blood glucose (reported in three subjects, 5.2%) (data not shown).

Of the 83 reported AEs, the single event judged to be “related” to study medication was in a subject experiencing Division of AIDS grade 1 nausea in the didanosine 400 mg/HC 600 mg arm that began on Day 7 and resolved without dose interruption or sequelae.

There were no significant signals found in laboratory chemistry or hematological parameters. A trend was noted in which the highest HC dose was associated with an increase in neutrophil percentage in both the half- and full-dose didanosine dose arms (data not shown).

### Genotypic Resistance

No changes in nucleoside analogue sensitivity were detected utilizing genotypic testing performed at baseline and following 28 days of therapy with VS411. No subject entered into the study with mutations associated with didanosine resistance nor did any such mutation emerge during therapy. All subjects were sensitive to a panel of 19 drugs at both baseline and following 28 days of treatment with VS411.

### HIV-1 RNA

**Figure 2:**
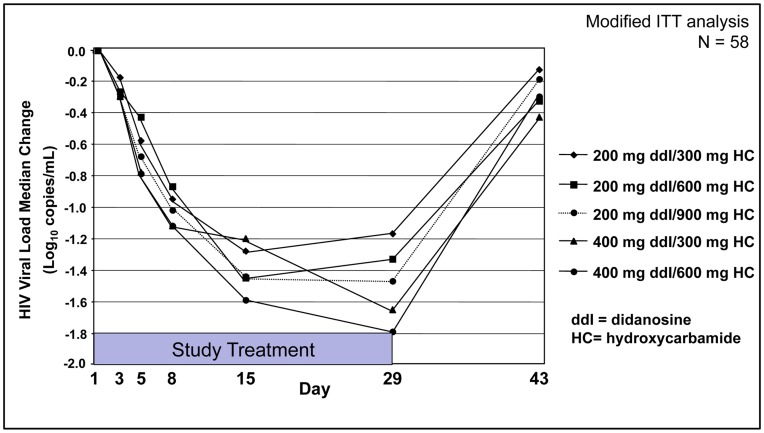
HIV-1 RNA (log_10_ copies/mL) median change from Day 1 to Day 29. Statistically significant (*P*<.001) HIV-1 RNA reductions were seen in each of the five VS411 dose arms with a median reduction in HIV-1 RNA across combined study arms at Day 29 of −1.47 log_10_. There was a trend toward greater reductions with increasing doses of both didanosine and hydroxycarbamide. Only two of 58 evaluable subjects (3%) achieved HIV-1 RNA reductions to <50 copies/mL by Day 29.

Evolution of the median change in HIV-1 RNA levels over time is reported per study arm in Figure 2. HIV-1 RNA levels reductions were seen in each of the five VS411 dose arms and the magnitude of each appeared to be dose dependent for both didanosine and HC. Only two of 58 mITT subjects (3%) achieved HIV-1 RNA reductions below the level of detectability (ie, <50 copies HIV-1 RNA/mL) by Day 29. There was a trend toward greater reductions with increasing doses of both didanosine and HC. In an mITT analysis of the half-dose didanosine (200 mg) arms, median HIV-1 RNA reductions at Day 29 ranged from −1.17 log_10_ copies/mL [range: −0.45 to −2.27 (*P* = .001)] in the didanosine 200 mg/HC 300 mg arm to −1.33 log_10_ [range: −0.66 to −2.26 (*P* = .001)] in the didanosine 200 mg/HC 600 mg arm and −1.47 log_10_ [range: −0.56 to −2.18 (*P* = .001)] in the didanosine 200 mg/HC 900 mg arm. In the full-dose didanosine arms, HIV-1 RNA reductions ranged from −1.65 log_10_ [range: −0.43 to −2.59 (*P* = .001)] in the didanosine 400 mg/HC 300 mg arm to −1.79 log_10_ [range: −0.93 to −2.33 (*P* = .001)] in the didanosine 400 mg/HC 600 mg arm.

Overall the magnitude of HIV-1 RNA reduction increased from Day 15 to Day 29 of the study. The median reduction across all combined study arms at Day 15 was −1.43 log_10_ copies/mL [range: −0.33 to −2.27 *(P*<.001)] and −1.46 log_10_ copies/mL [range: −0.43 to −2.59 *(P*<.001)] at Day 29. Following study medication termination at Day 29, HIV-1 RNA levels returned to near baseline after two weeks off therapy (Day 43) in all treatment arms.

### CD4^+^ T Cell Counts

**Figure 3:**
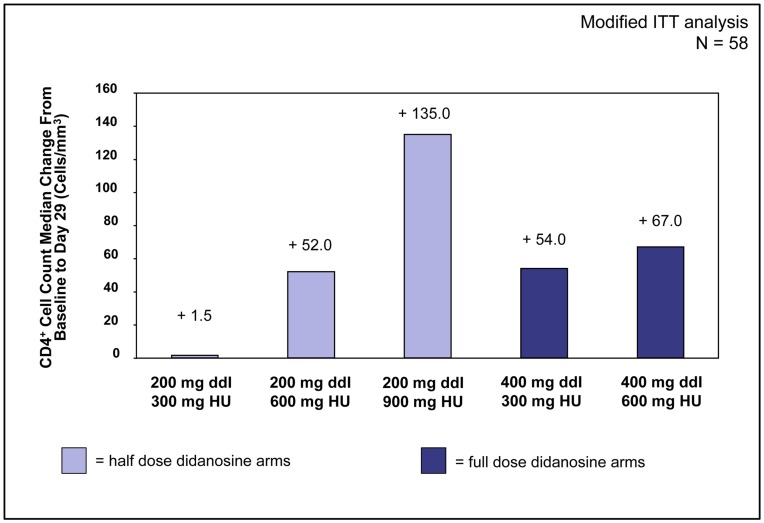
CD4^+^ T cell count (cells/mm^3^) median change from Day 1 to Day 29. The median change in CD4^+^ T cell count for the combined study arms after 28 days of treatment was +53 cells/mm^3^ in the mITT analysis (*P*<.002). There was a trend toward a dose-response relationship seen for HC in both the half-dose and the full-dose didanosine arms.

The median change in CD4^+^ T cell count for the entire cohort after 28 days of treatment was +53 cells/mm^3^ [range: −405 to +494 *(P*<.001)] in the mITT analysis. There was a trend toward a dose-response relationship seen for HC in both the half-dose and the full-dose didanosine arms (Figure 3). In the half-dose didanosine arms, median changes in CD4^+^ T cell count increases after 28 days of therapy ranged from +1.5 cells/mm^3^ [range: −88 to +368 (*P* = .27)] in the didanosine 200 mg/HC 300 mg arm to +52 cells/mm^3^ [range: −118 to +248 (*P* = .07)] in the didanosine 200 mg/HC 600 mg group and +135 cells/mm^3^ [range: −405 to +494 (*P* = .23)] in the didanosine 200 mg/HC 900 mg arm, the greatest median increase in CD4^+^ T cell count of the study. In the two full-dose didanosine arms, median increases in CD4^+^ T cell counts were +54 cells/mm^3^ [range: −113 to +468 (*P* = .20)] in the didanosine 400 mg/HC 300 mg arm and +67 cells/mm^3^ [range: −131 to +224 ( = .14)] in those receiving didanosine 400 mg/HC 600 mg.

### Immune Activation Markers

Changes in the expression of four surface markers on the CD3^+^ cell subset (CD4^+^ and CD8^+^ T cells) from baseline to Day 29 in a sub-study of 32 subjects are shown in Table 6. Rapid and significant reductions were seen in all four markers of excessive immune system activation. After 28 days of therapy with VS411, Ki-67 (a proliferation marker), PD-1 (programmed cell death-1, an exhaustion marker), CD38 (cluster of differentiation 38, an activation marker), and HLA-DR (human leukocyte antigen-D related, an activation marker) expression levels were significantly reduced by 29.0%, 26.1%, 9.7%, and 19.3%, respectively (*P*<.005 for all).

**Table 6:** Changes in markers of excessive immune system activation after 28 days of treatment with VS411.

CD3^+^ subset (n = 32)	Baseline (mean ± SD^a^)	Day 29 (mean ± SD)	% Reduction	*P* value
**Ki-67%**	3.1±1.7	2.2±0.9	29.0	<.005
**PD-1%**	15.3±6.1	11.3±5.0	26.1	<.005
**CD38%**	58.5±14.8	52.8±14.2	9.7	<.005
**HLA-DR %**	27.5±10.1	22.2±7.5	19.3	<.005

a SD  =  Standard Deviation.

**Figure 4:**
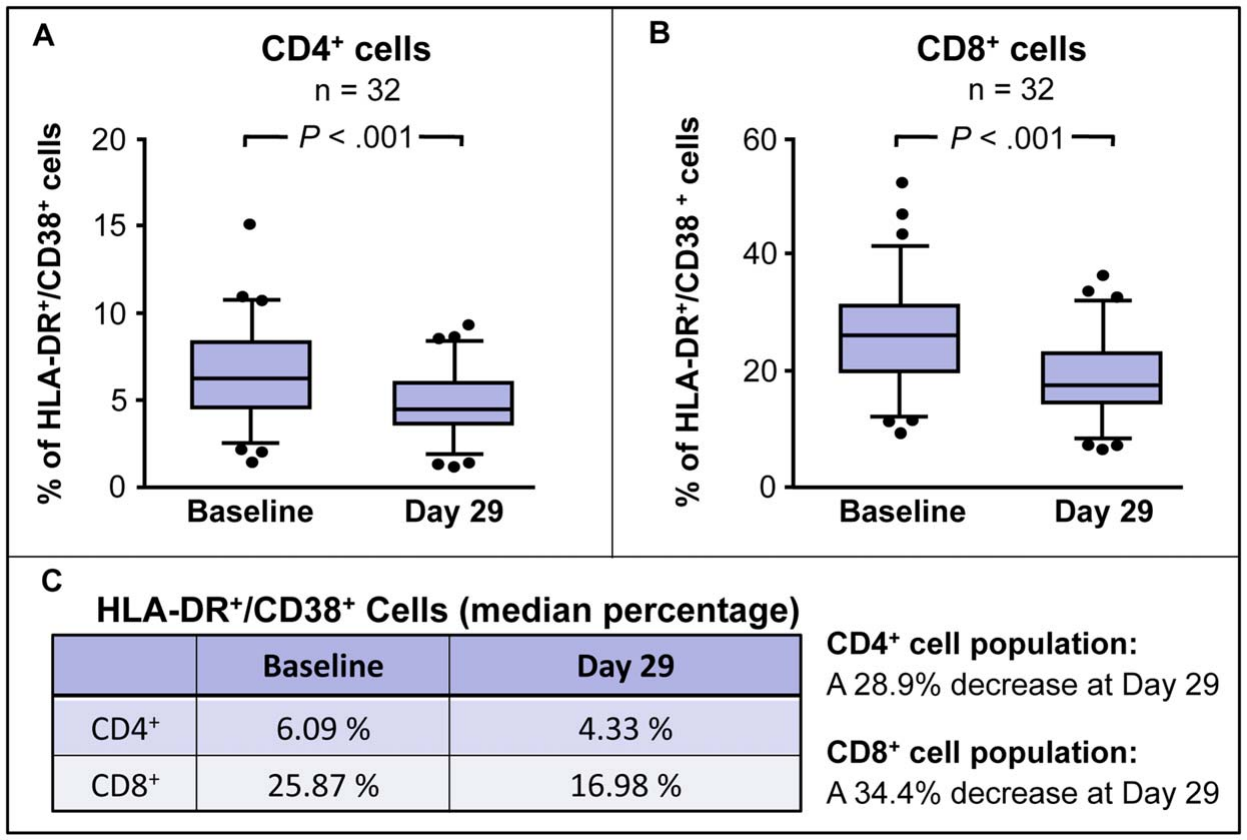
CD4^+^ and CD8^+^ T cells co-expressing HLA-DR and CD38. In a sub-study of 32 of the 56 subjects completing VS411 for 28 days, rapid and statistically significant reductions were seen in the percentage of CD4^+^ (A) and CD8^+^ (B) cells co-expressing the two markers of cellular activation, CD38 and HLA-DR. After 28 days of VS411 treatment, the median percentage of CD4^+^ and CD8^+^ T cells co-expressing the two markers (C) decreased by 28.9% (from 6.09% to 4.33%) and 34.4% (from 25.87% to 16.98%), respectively (*P*<.005). These statistically significant reductions were obtained despite the fact that VS411 did not attain maximal suppression of HIV-1 RNA, suggesting the effect was due to the HALT component of this AV-HALT combination. In (A) and (B) the grey boxes span the 25^th^ and 75^th^ percentile values. The error bars span the 10^th^ and the 90^th^ percentile values. Each midline represents the median value. The dots represent individual observations below the 10^th^ and above 90^th^ percentile values.

In the same sub-study, rapid and statistically significant reductions were seen in the number of CD4^+^ and CD8^+^ T cells co-expressing the two markers of cellular activation, CD38 and HLA-DR (Figure 4), a measurement conventionally used in several clinical trials [2], [42]. At Day 29, the median percentage of CD4^+^ and CD8^+^ T cells co-expressing the two markers decreased by 28.9% (from 6.09% to 4.33%) and 34.4% (from 25.87% to 16.98%), respectively (*P*<.001 for each).

### Immunosuppressive Effect on HIV-1-specific Immune Responses

**Figure 5:**
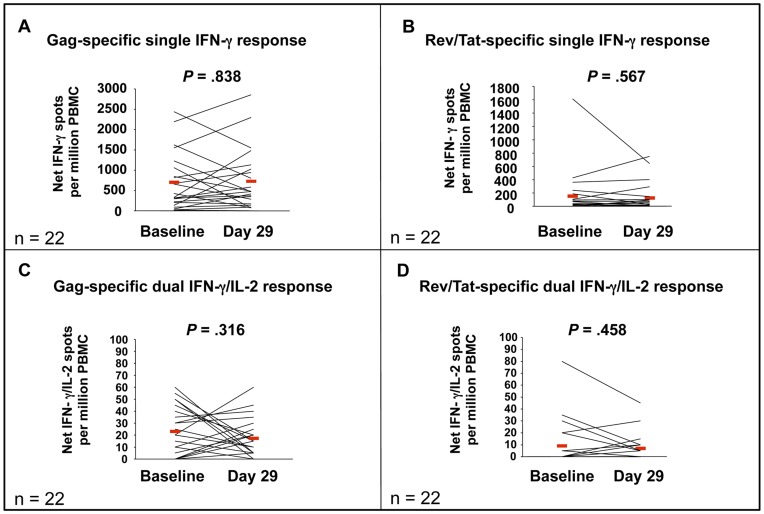
IFN-γ and dual IFN-γ/IL-2 measured by ELIPSOT at Baseline and Day 29. A sub-study of 22 individuals was conducted to evaluate whether VS411 induced an immunosuppressive effect on T lymphocytes. IFN-**γ** responses specific for Gag (A) and for Rev/Tat (B) and dual IFN-**γ**/IL-2 responses specific for Gag (C) and for Rev/Tat (D) at baseline and after 28 days treatment (Day 29) were measured by ELIPSOT (spots/million PBMCs). Horizontal red lines indicate mean values. There were no significant differences seen between pre- and post-therapy analyses suggesting that VS411 had no immunosuppressive effect on HIV-1-specific immune responses.

In Figure 5, single IFN-**γ** responses specific for Gag (A) and for Rev/Tat (B) and dual IFN-**γ**/IL-2 responses for Gag (C) and for Rev/Tat (D) in a sub-study of 22 individuals are shown. Higher responses were observed for Gag compared to Rev/Tat. Responses detected at Day 29 were similar to those at baseline, suggesting there was no immunosuppressive effect from the therapy over the course of the study.

## Discussion

Current antiretroviral treatments for HIV-1 infection have been successful in reducing HIV-1 RNA levels, in many cases to below the level of detection. However, recent discoveries in HIV disease pathogenesis, combined with long-term observations of individuals who experience increased morbidities and mortality while maintaining undetectable HIV-1 RNA levels on ART regimens, suggest that these therapies may not be adequately treating the entire disease state. To do so, future treatment strategies must be disease modifying, seeking to significantly reduce the state of chronic immune hyperactivation that exists in HIV disease and results in the loss of CD4^+^ T cells and the emergence of both AIDS-defining and non-AIDS complications such as cardiovascular disease, cancer, liver or kidney failure, and cognitive disorders reported in HIV-1-infected individuals receiving long-term ART.

VS411 represents the first entry in a new class known as AV-HALTs that seeks to combine traditional antiviral efficacy (AV) with novel, potentially beneficial reductions in the excessive immune system activation (HALT) associated with HIV-1 disease. Unlike VS411 that possesses both AV and HALT properties, other drugs under evaluation to address immune system hyperactivation, such as atorvastatin, leflunomide and chloroquine (CQ), are thought to possess only the HALT (or HALT-like) activity. As regulatory authorities require antiretrovirals to demonstrate significant reductions in HIV-1 RNA for approval, it will be difficult for agents such as these that lack direct antiviral activity to achieve an HIV indication.

The effects of statins (HMG-CoA reductase inhibitors) on HIV-1 infection have been studied [43], [44], [45]. In a Phase 2 clinical trial with HIV-1-infected, ART-naïve individuals receiving high dose atorvastatin for eight weeks, modest but significant reductions in immune activation markers (CD38 and HLA-DR) on T cells were observed although HIV-1 RNA levels were unaffected [46].

Another pilot study of the immunomodulatory agent leflunomide for 28 days was performed in HIV-1-infected persons not receiving antiretroviral therapy. Although there was no statistically significant decrease in plasma HIV-1 RNA, treatment with leflunomide reduced expression of activation markers (HLA-DR/CD38 co-expression) on CD8^+^ T cells, suggesting that targeting immune activation with immunomodulatory agents may be a feasible strategy, although the presence of the hyperactivation limiting component alone does not seem to be effective in suppressing HIV replication [47].

CQ significantly reduced the frequency of CD38^+^HLA-DR^+^CD8^+^ T cells as well as Ki-67 expression in CD8^+^ and CD4^+^ T cells after one month of therapy [48]. In another study, treatment with a related drug, hydroxychloroquine, lowered several parameters of immune activation in immunological non-responders [49].

The novelty of the AV-HALT approach is illustrated by the fact that VS411 was specifically designed as a combination of two drugs – one an “AV,” the other a “HALT” – to simultaneously and efficiently target both excessive immune activation and viral replication. In this Phase 2a study, VS411 successfully achieved Proof-of-Concept for the new AV-HALT class.

VS411 safely lowered HIV-1 replication (antiviral “AV” effect) over 28 days by a median of 1.5 log_10_ without selecting for antiretroviral resistance. The lack of selection for drug resistance was expected as, unlike traditional antiretrovirals, HC targets a highly conserved human cellular enzyme (ribonucleotide reductase) with little, if any, potential for selection of resistance [22]. Moreover, viruses genotypically resistant to didanosine regain phenotypic sensitivity in the presence of HC [36], [50].

VS411 significantly increased median CD4^+^ T cell counts over 28 days by as much as 135 cells/mm^3^. Reducing the doses of HC contained in VS411 from those traditionally administered in the treatment of HIV disease allowed the agent to act as a cytostatic, rather than cytotoxic, drug. As a result, the “blunting” of CD4^+^ T cell increases historically seen with the didanosine/HC combination was not seen with VS411. In fact, the largest CD4^+^ T cell count increase was seen in the didanosine 200 mg/HC 900 mg arm in which doses of both drugs were lowered from their historic dosing.

This 28-day Phase 2a study also incorporated pre- and post-therapy measurements of four accepted markers of immune system activation – Ki-67, PD-1, CD38, and HLA-DR whose expressions were rapidly and significantly reduced by 29.0%, 26.1%, 9.7% and 19.3%, respectively, despite incomplete suppression of HIV-1 RNA levels, suggesting that the effect was due to the HALT component of this AV-HALT combination. After only 28 days of treatment with VS411, the median percentages of CD4^+^ and CD8^+^ T cells co-expressing CD38 and HLA-DR were significantly decreased, reaching levels similar to those documented in HIV-1-infected subjects receiving 20 months of ART and with plasma HIV-1 RNA below 1000 copies/mL [2]. Interestingly, the didanosine 200 mg/HC 900 mg arm reduced T cell activation nearly to the level reported for Elite Controllers, for both CD4^+^ T cells (4.4% versus 3.8%, respectively) and CD8^+^ T cells (16.0% versus 15.5%, respectively) [42]. These results were achieved in a population mirroring the global pandemic: 50% female and 50% black from both the northern and southern hemispheres.

Two commercially available drugs, didanosine and HC, were combined into VS411 to be used in this study to test the concept that it is possible to rapidly reduce markers of cellular hyperactivation and proliferation with the new AV-HALT class. Although judged to be safe for short-term use as in this study, this two-drug combination is not, however, a candidate for further drug development or as a treatment for HIV-1 disease due to significant long-term toxicities now associated with didanosine including pancreatitis, lactic acidosis/severe hepatomegaly with steatosis, and non-cirrhotic portal hypertension [51].

The ultimate goal is to discover and develop single-molecule compounds that provide both the “AV” and “HALT” activities. Based upon this successful Proof-of-Concept study utilizing VS411, additional work is now underway to screen, identify and develop new agents that combine both attributes of AV-HALTs, a direct reduction of HIV-1 RNA with reductions of markers of excessive immune activation in a single molecule. By combining the clinical results obtained with VS411 with the in vitro profile of the two drugs comprising it, VS411 can now function as a comparator prototype for identifying additional AV-HALTs. Potential candidates for these ‘second-generation’ AV-HALTs include molecules targeting additional cellular factors involved in the cell cycle and cellular proliferation that are also known to play critical roles in the life cycle of HIV-1 and other viruses. By targeting human rather than viral enzymes, these single-molecule AV-HALTs could provide additional benefits such as a lower propensity to select for viral resistance and a lack of cross resistance and cross toxicities with currently marketed antivirals.

### Conclusion

In this Phase 2a study, the two-drug, first-generation AV-HALT VS411 successfully established the Proof-of-Concept that AV-HALTs can combine antiviral efficacy with rapid and potentially beneficial reductions in the excessive immune system activation associated with HIV-1 disease. The rapid reductions in markers of immune system hyperactivation and cellular proliferation were obtained despite the fact that VS411 did not attain maximal suppression of HIV-1 RNA levels, suggesting this effect was due to the HALT component. Although VS411 is not being developed further due to concerns over the long-term toxicities associated with didanosine, VS411 can serve as a comparator AV-HALT prototype reflecting both the desired in vitro and clinical attributes of the this new class. Work is now underway to screen, identify and develop single-molecule, second-generation AV-HALTs as disease-modifying therapies for addition to the armamentarium targeting the HIV and other viruses.

## Supporting Information

Checklist S1 **CONSORT Checklist.**
(DOC)

Protocol S1. **Trial Protocol**
(PDF)

Sub-Study Demographics S1 **Sub-Study Demographics Table.**
(DOCX)
