# Supplementary material for: VS411 Reduced Immune Activation and HIV-1 RNA Levels in 28 Days: Randomized Proof-of-Concept Study for AntiViral-HyperActivation Limiting Therapeutics
Source: PLoS One. 2012 Oct 19;7(10):e47485. doi: 10.1371/journal.pone.0047485 (PMC3477169; doi:10.1371/journal.pone.0047485)
Supplement: Protocol S1. — Trial Protocol (PDF) [file pone.0047485.s002.pdf]

SPONSOR	VIROSTATICS
**CLINICAL STUDY PROTOCOL**<br>(EudraCT number: 2007-002460-98)	
**SPONSOR PROTOCOL NUMBER**	VS411-C201
**IATEC STUDY NUMBER**	07-IAT-179
**PRODUCT/ COMPOUND**	VS411 / Hydroxyurea + Didanosine
**TITLE**	**A randomized double-blind dose-finding multi-centre phase IIa study with VS411 for HIV-1 infection.**
**CLINICAL PHASE**	Phase IIa
**INDICATION**	HIV-1
**PROTOCOL CHAIRS**	J. Lange, MD PhD<br>Center of Poverty Related and Communicable Diseases,<br>Academic Medical Centre<br>The Netherlands<br><br>F. Lori, MD<br>Director<br>Research Institute for Genetic and Human Therapy (RIGHT), Pavia, Italy
**IATEC PROJECT MANAGER**	A. S. Bergshoeff, PharmD, PhD
**PROTOCOL VERSION & DATE**	Final version, October 15th, 2007

This confidential document is the property of IATEC BV. No unpublished information contained in this document may be disclosed without prior written approval of IATEC BV and the study sponsor.

TABLE OF CONTENTS

1. SIGNATURES 5

INVESTIGATOR'S SIGNATURE 6

2. CONTACT DETAILS 7

3. ABBREVIATIONS AND DEFINITIONS OF TERMS 9

4. STUDY FLOW CHART AND SCHEDULE OF PK ASSESSMENTS 15

5. INTRODUCTION 18

5.1. Unique Mechanisms of Action to Address an Unmet Medical Need 18

5.2. A Better Understanding of the Factors that Contributed to the Toxicity of Hydroxyurea and Didanosine 19

5.2.1. Combination with Stavudine (Zerit® - d4T) 20

5.2.2. Lack of Weight-based Dosing Adjustments for Stavudine and Didanosine 20

5.2.3. Higher Didanosine Cmax as QD Tablets Replaced BID 21

5.2.4. Increasing Doses of Hydroxyurea 21

5.2.5. Didanosine Potentiation of Hydroxyurea 21

5.2.6. Prior History of Pancreatitis and Elevated Markers 22

5.2.7. VS411 – Dosing Based Upon New Understandings 22

6. STUDY OBJECTIVES AND PURPOSE 23

6.1. Objectives 23

7. STUDY POPULATION 24

7.1. Number and type of subjects 24

7.2. Inclusion criteria 24

7.3. Exclusion criteria 24

7.4. Withdrawal and replacement criteria 25

8. STUDY DESIGN 27

8.1. Overall design 27

8.2. Rationale for study design 28

8.2.1. Endpoints and study duration 28

8.2.2. Dosing rationale 29

8.2.3. Sample size rationale 30

8.3. Randomization, blinding and treatment allocation 30

9. INVESTIGATIONAL PRODUCT 31

9.1. Study Medication 31

9.2. Packaging and labeling 32
9.3. Handling and storage 32
9.4. Responsibilities 32
9.5. Accountability 33

10. STUDY CONDUCT **33**
10.1. Study procedures at each visit 33
10.1.1. Screening visit (visit 1) 33
10.1.2. Baseline and follow up visits 34
10.2. Assessment of safety 38
10.3. Assessments of efficacy 40
10.4. Concomitant medication 40

11. ADVERSE EVENTS AND OTHER SAFETY ASPECTS **40**
11.1. Definitions 40
11.2. Suspected unexpected serious adverse reactions (SUSAR) 42
11.3. Pregnancy notifications 43
11.4. Annual safety report 43
11.5. Expedited reporting of SAEs and SUSARs 43
11.6. Follow-up of SAEs 44
11.7. Emergency procedures and management of overdose 44
11.8. Emergency breaking of treatment code 45

12. STATISTICS **45**
12.1. Statistical methods 45
12.2. Planned interim analysis 46
12.3. Determination of sample size 46
12.4. Deviations from statistical analysis plan 46
12.5. Subject population to be analyzed 46

13. PHARMACOKINETICS **47**
13.1. Sample collection and handling 47
13.2. Bioanalysis 47
13.3. Noncompartmental Pharmacokinetic Analyses 48

14. DATA MANAGEMENT **49**
14.1. Direct access to source data 49
14.2. Data collection 49
14.3. Monitoring 50

14.4. Data management 50
14.5. Quality Assurance 51

15. ADMINISTRATIVE MATTERS **51**
15.1. Financing and Insurance 51
15.2. Investigator Indemnity 51
15.3. Regulatory and Ethics Committee approval 52
15.4. Subject information and informed consent 52
15.5. (Subject) Confidentiality 53
15.6. Amendments 54
15.7. Annual progress report 54
15.8. End of study report 54
15.9. Publication policy 55
15.10. Study documentation and record keeping 55
15.11.Sample handling 56

16. REFERENCES **58**

APPENDIX I: CDC CLASSIFICATION SYSTEM FOR HIV INFECTION (REVISION 1993) **59**

APPENDIX II: TABLE FOR GRADING ADVERSE EXPERIENCES **62**

APPENDIX III: LIST OF CONTRAINDICATED MEDICATION **82**

1. SIGNATURES

For the protocol entitled: "A randomized double-blind dose-finding multi-centre phase IIa study with VS411 for HIV-1 infection".

PROTOCOL AUTHORISED BY:

Sponsor or Legal Representative:	
S. Petrocchi	Signature: [signature]
CEO of Virostatics	Date: 22-oct-2007
Coordinating Principal Investigator:	
J. Lange, MD PhD	Signature: [signature]
Academic Medical Centre	Date: 29-10-07
CEO IATEC:	
F. Ex, Msc	Signature: [signature]
IATEC BV	Date: 10-OCT-2007
Manager Medical Affairs	
C.F.P. van Ede, Msc	Signature: [signature]
IATEC BV	Date: 19-oct-2007
Director Biometrics:	
F. Smout, Msc	Signature: [signature]
IATEC BV	Date: 24-okt-2007

PROTOCOL AUTHORS:

F. Wit, MD PhD; CPCD

K. Bol, PhD; Kinesis

E. Hassink, MSc PhD: IATEC BV

INVESTIGATOR'S SIGNATURE

For the protocol entitled: "A randomized double-blind dose-finding multi-centre phase IIa study with VS411 for HIV-1 infection".

I have read all pages of this clinical study protocol for which Virostatics is the sponsor. I agree that it contains all the information required to conduct this study. I agree to conduct the study as outlined in this protocol and to comply with all terms and conditions set out therein.

I confirm that I will conduct the study in accordance with ICH GCP guidelines (ICH topic E6 adopted 01-05-1996, implemented 17-01-1997) and the provisions of the Helsinki Declaration (version 1996), a copy of which has been given to me by the sponsor. I will also ensure that sub-investigators and other relevant members of my staff have access to this protocol and the Helsinki Declaration to enable them to work in accordance with the provisions of these documents.

Principal Investigator:

Signature: .... Date: ....

Name:..

Institution:.......................................

Country:...

2. CONTACT DETAILS

**Sponsor:**	Virostatics, S.R.L.<br>Viale Umberto I, 46<br>07100 Sassari (SS)<br>Italy
**Protocol chairs:**	J. Lange, MD PhD<br>Department of Internal Medicine,<br>Division of Infectious Diseases, Tropical<br>Medicine and AIDS<br>Academic Medical Center<br>Meibergdreef 9 1105 AZ Amsterdam<br>The Netherlands<br><br>F. Lori, MD<br>Director<br>Research Institute for Genetic and Human<br>Therapy (RIGHT), Pavia, Italy

**CRO for monitoring**	IATEC BV<br>Pietersbergweg 9<br>1105 BM Amsterdam<br>The Netherlands
**Medical Monitoring & Pharmacovigilance**	C. F.P. van Ede<br>Manager Medical Affairs<br>IATEC BV<br>Phone. +31 20 3149 324 or<br>Fax +31 20 3149 399<br>E-mail: c.vanede@iatec.com
**Fax number for reporting SAEs**<br>**Telephone number for reporting SAEs**	**+31 20 3149 394**<br><br>**+31 20 3149 375**
**Project Manager**	A. S. Bergshoeff<br>IATEC BV<br>Phone. +31 20 3149 300 (secr.) or<br>+31 20 3149 348 (direct)<br>Fax +31 20 3149 399
**Study Monitor**	Clinical Research Associate<br>IATEC BV<br>Phone. +31 20 3149 300<br>Fax +31 20 3149 399

3. ABBREVIATIONS AND DEFINITIONS OF TERMS

ACTG	AIDS Clinical Trials Group
AE	Adverse Event
AIDS	Acquired Immunodeficiency Syndrome
ALAT	Alanine Aminotransferase
ASAT	Aspartate Aminotransferase
AUC	Area under the curve
BID	Twice daily
BP	Blood pressure
CDC	Centers for Disease Control and Prevention
CIOMS	Council for International Organization of Medical Sciences
C_{max}	maximum concentration
C_{min}	minimum concentration (trough concentration)
CPK	Creatinine Phosphate Kinase
CRF	Case Report Form
CV	Coefficient of variation
DAIDS	Division of AIDS
d4T	Stavudine
dATP	Deoxyadenosine triphosphate
ddATP	Dideoxyadenosine triphosphate
ddI	Didanosine
DNA	Deoxyribonucleic acid
dNTP	Deoxyribonucleotide triphosphate
DSMB	Data Safety and Monitoring Board
EC	Enteric coated
EDTA	Ethylenediaminetetraacetic acid
ELISA	Enzyme-Linked ImmunoSorbent Assay
EMEA	European Medicines Agency
GCP	Good Clinical Practice
FDC	Fixed Dose Combination
GMP	Good Manufacturing Practice
γ-GT	Gamma-Glutamyl Transferase
HAART	Highly Active Antiretroviral Therapy

hCG	Human chorionic gonadotropin
HCV	Hepatitis C Virus
HIV	Human Immunodeficiency Virus
HR	Heart Rate
HU	Hydroxyurea
IB	Investigator's Brochure
ICH	International Conference on Harmonization
IC_{xx}	Inhibitory concentration (where xx equals the percent inhibition)
IEC	Independent Ethics Committee
IM	Intramuscular
IMP	Investigational Medicinal Product
ITT	Intent-to-treat
MedDRA	Medical Dictionary for Regulatory Activities
MCV	Mean corpuscular volume
NRTI	Nucleoside Reverse Transcriptase Inhibitor
PBMC	Peripheral Blood Mononuclear Cells
PCR	Polymerase Chain Reaction
PK	Pharmacokinetic(s)
PD	Pharmacodynamic(s)
QA	Quality Assurance
QD	Once daily
RNA	Ribonucleic acid
SAE	Serious Adverse Event
SD	Standard deviation
SmPC	Summary of Product Characteristics
SOP	Standard Operating Procedure
SUSAR	Suspected Unexpected Seriuos Adverse Reaction
$t_{1/2}$	Half-life
$t_{1/2term}$	Terminal half-life
ULN	Upper Limit of Normal
Vd	Volume of distribution
Vd/F	Volume [of drug] distribution per fraction of bioavailable
WBC	White Blood Cells
WHO	World Health Organization

STUDY SYNOPSIS

**Name of Sponsor:**	VIROSTATICS
**Name of finished product:**	VS411: A Fixed Dose Combination (FDC) of hydroxyurea and didanosine
**Title of study:**	A randomized double-blind dose-finding multi-centre phase IIa study of VS411 for HIV-1 infection.
**Protocol Chair:**<br>**Affiliation:**	Prof. J.M.A. Lange<br>Department of Internal Medicine,<br>Division of Infectious Diseases, Tropical Medicine and AIDS<br>Academic Medical Center<br>Meibergdreef 9 1105 AZ Amsterdam<br>The Netherlands
**Planned study period:**	Enrolment mid January 2008 – mid May 2008
**Clinical Phase:**	Phase IIa
**Objectives:**	To characterize five different dose combinations of hydroxyurea plus didanosine in the form of VS411 for further evaluation in phase IIb studies with respect to:<br>- HIV-1 antiviral activity;<br>- Pharmacokinetics (PK);<br>- Pharmacokinetic and pharmacodynamic (PD) relationships;<br>- Quantification of intracellular ddATP / dATP;<br>- Immunological parameters;<br>- Safety / tolerability;<br>- Evaluation of genotypic resistance to nucleoside/nucleotide analogues at baseline and after 4 weeks treatment.
**Methodology and treatment arms:**	This is a randomized, five-arm, double-blind, multi-centre, Phase IIa study to identify the optimal dosing schedule(s) of hydroxyurea plus didanosine in the treatment of HIV-1 infection. Subjects will be treated with the study regimen for four weeks, after which they will discontinue study treatment and continue without antiretroviral therapy or start standard-of-care antiretroviral therapy. Follow-up will continue for

	another two weeks to gather safety data.<br>Subjects are randomized in a 1:1:1:1:1 ratio to one of the following five study arms:<br>- Didanosine 400 mg QD plus hydroxyurea 600 mg QD;<br>- Didanosine 200 mg QD plus hydroxyurea 600 mg QD;<br>- Didanosine 200 mg QD plus hydroxyurea 300 mg QD;<br>- Didanosine 400 mg QD plus hydroxyurea 300 mg QD;<br>- Didanosine 200 mg QD plus hydroxyurea 900 mg QD.
**Number of subjects planned:**	60 subjects will be recruited for this study (12 per treatment arm)
**Main criteria for inclusion:**	- Provide written informed consent;<br>- Chronically HIV-1 infected patients;<br>- At least 18 years of age;<br>- Males or non-pregnant, non-lactating females;<br>- HIV-1 antiretroviral treatment naive;<br>- Plasma HIV-1 RNA levels > 5000 copies/mL;<br>- $CD4^+$ T-cell count > 250 cells/mm^3;<br>- CDC AIDS Surveillance Case Definition classification Stage A or B;<br>- Use of adequate and reliable forms of contraception during the study (males and females of childbearing potential) and starting at least one month prior to study drug administration for females and continuing for 30 days after discontinuation of study medication.
**Main criteria for exclusion:**	- HIV-2 co-infection;<br>- Primary (acute) HIV-1 infection;<br>- < 60 kg bodyweight;<br>- Clinically relevant laboratory abnormalities: anemia (Hb <10g/dL, thrombocytopenia (platelets < 100,000 per mm^3), leucopenia (WBC< 3,000 per mm^3), elevated triglycerides (> 2 times the ULN), elevated liver transaminases (> 2 times the ULN), elevated bilirubin (> 2 times the ULN), elevated amylase (> 2 times the ULN), elevated lipase (> 2 times the ULN), decreased estimated creatinine clearance (< 60 mL/min) using the

	Cockcroft-Gault formula;<br>- Use of co-medication with a known clinically significant pharmacological interaction with one or more of the study drugs (use of proton pump inhibitors are not allowed);<br>- Active alcohol or drug abuse (methadone use is allowed);<br>- Anticipated non-compliance with the protocol;<br>- Anticipated need to start HAART within the study period;<br>- Presence of a newly (within 30 days) diagnosed HIV-related opportunistic infection or condition requiring acute therapy at the time of enrolment;<br>- Patients who have taken any investigational drug 30 days prior to the start of the study;<br>- Chronic active viral hepatitis;<br>- Pregnancy;<br>- Current or former pancreatitis;<br>- History of, or currently receiving, interferon therapy;<br>- Presence of didanosine-associated resistance mutations;<br>- Current or recent (within the prior three months) use of immunomodulatory agents (including vaccines);<br>- Current or recent (within the prior three months) use of ribavarin;<br>- Known allergy to the active or inactive components of VS411.
**Duration of treatment:**	The total duration of the study will be six weeks plus a screening period of maximally four weeks. Duration of treatment will be four weeks plus two weeks of safety follow-up.
**Main criteria for evaluation:**	- Changes in plasma HIV-1 RNA concentrations<br>- Plasma PK/PD of VS411<br>- Quantification of intracellular ddATP / dATP<br>- Changes in $CD4^+$ T-cell counts<br>- Incidence and severity of adverse events

	- Evaluation of genotypic resistance to nucleoside/nucleotide analogues at baseline and after four weeks of treatment
**Statistical methods:**	Data will be described by means and standard deviations or median and interquartile ranges, whatever is applicable.<br>The changes from baseline in log-transformed plasma HIV-1 RNA levels and immunological parameters will be analyzed by a repeated measurements procedure using a generalized linear model (PROC MIXED of SAS software, SAS version 9.1), which provides a valid statistical estimate of the mean effect. The incidence of adverse events and laboratory abnormalities will be tabulated. All tests of hypotheses will be two-sided and will be at the 5% level of significance. The above described analyses will be performed on the intent-to-treat population and on the on-treatment population (all subjects who took more than 75% of the study's doses).<br>In addition, an integrated dose-response and exposure-response analysis will be performed on the intent-to-treat population to characterize the relationships between different dose combinations of hydroxyurea and didanosine, their plasma concentrations and their effects on HIV-1 viral load in time (using a non-linear mixed effect modeling procedure).
**Pharmacokinetics**	Determination of PK parameters of hydroxyurea and ddI by non-compartmental analysis.

Visit	V1 Screening	V2 Baseline	V2 + 1 day group A patients only	V3	V4	V5	V6	V6 + 1 day group B patients only	V7	V8
**Day ± window**	**-28 to -7**	**1**	**2**	**3**	**5**	**8**	**15 ± 1**	**16 ± 1**	**29 - 1**	**43± 1**
Safety laboratory - haematology	4.5 mL	4.5 mL		4.5 mL	4.5 mL	4.5 mL	4.5 mL		4.5 mL	4.5 mL
- chemistry	4.5 mL	4.5 mL		4.5 mL	4.5 mL	4.5 mL	4.5 mL		4.5 mL	4.5 mL
- lactate[2]		2 mL							2 mL	
Genotypic resistance: plasma (EDTA) storage [3]	4.5 mL	4.5 mL							4.5 mL	
HIV-1 RNA: plasma storage [4]	7 mL	7 mL		7 mL	7 mL	7 mL	7 mL		7 mL	
PBMC - intracellular concentrations ddI		40 mL					40 mL			
Viable PBMC - extensive immunology	30 mL								30 mL	
Full PK plasma day curves [1] - Group A (A)		9 mL					24 mL	3 mL		
- Group B (B)		24 mL	3 mL				9 mL			
Single sample for PK									3 mL	
Plasma (EDTA) for general storage	4.5 mL	4.5 mL		4.5 mL	4.5 mL	4.5 mL	4.5 mL		4.5 mL	4.5 mL
Serum (Chronic active viral hepatitis B and chronic hepatitis C)	4.5 mL									
Amount of blood = 361 mL/ subject) (A)/(B)	59.5 mL	76/91 mL	3 mL	20.5 mL	20.5 mL	20.5 mL	84.5/69.5 mL	3 mL	60 mL	13.5 mL

[1] Subjects will be divided in group A (A) and group B (B) for the PK assessments, see detailed schedule on page 17.

[2] Sample should preferably be taken from patient in rest and not earlier than 30 minutes after heavy physical exercise, if the latter is applicable.

[3] At screening the genotypic resistance will be assessed locally if required. At other visits, plasma will be stored for central assessment of genotypic resistance.

[4] At screening the HIV-RNA will be assessed locally. At other visits plasma will be stored for central assessment of plasma HIV-RNA.

4. STUDY FLOW CHART AND SCHEDULE OF PK ASSESSMENTS

Visit (Group [1])	V1 Screening	V2 Baseline	V2 + 1 day group A patients only	V3	V4	V5	V6	V6 + 1 day group B patients only	V7	V8
**Day ± window**	**-28 to -7**	**1**	**2**	**3**	**5**	**8**	**15 ± 1**	**16 ± 1**	**29 - 1**	**43± 1**
Study medication[1]	None	As randomized, last study medication intake at day 28							None	
Informed consent	x									
Pregnancy test	x	x							x	
Demographic data	x									
Inclusion and exclusion criteria	x									
Weight	x	x					x		x	
Height	x									
Full physical examination		x				x	x		x	x
Targeted physical examination[2]	x			x	x					
Medical / medication history	x									
Randomization		x								
Concomitant medication		x		x	x	x	x		x	x
CDC events and AEs		x		x	x	x	x		X	x
$CD4^+$ and $CD8^+$ T-cells[3]	x	x		x	x	x	x		x	X

[1] Study medication will be distributed during V2, V3, V4, V5 and V6

[2] A targeted physical exam will only be performed in case of adverse events and/or clinically relevant signs and symptoms, upon discretion of the physician

[3] Immunology assessments are done on haematology samples and therefore require no additional blood sampling

Schedule of PK assessments

Group A will have sparse PK assessments at V2 and full PK assessments at V6 and V6.+ 1 day. Group B will have full PK assessments for ddI and HU at V2 and V2 + 1 day and sparse PK assessments at V6. In both groups a single PK sample will be taken at V7. Samples to determine intracellular PK of ddI and HU will be taken at V2 and V6. The first 30 randomized patients will be assigned to Group A, the second 30 randomized patients will be assigned to Group B. (Of note, Based on the results of the bioavailability study (VS411-C101) the sampling schedule may be adjusted without changing the number of samples and the total blood volume to be drawn).

Group A

**Hours after drug intake (morning)**<br>**Actual time (h:mm)**	**Pre-dose**<br>**8:00**	**0.5**<br>**8:30**	**1.0**<br>**9:00**	**2.0**<br>**10:00**	**3.0**<br>**11:00**	**4.0**<br>**12.00**	**6.0**<br>**14:00**	**9.0**<br>**17:00**	**24.0**<br>**(pre-dose)**<br>**8:00**
Day 1 plasma PK			3 mL	3 mL		3 mL			
Day 15 and Day 16 (± 1) plasma PK	3 mL	3 mL	3 mL	3 mL	3 mL	3 mL	3 mL	3 mL	3 mL
Day 29 (-1) plasma PK	3 mL								
Day 1 and Day 15 (± 1) intracellular PK	40 mL								

Group B

**Hours after drug intake (morning)**<br>**Actual time (h:mm)**	**Pre-dose**<br>**8:00**	**0.5**<br>**8:30**	**1.0**<br>**9:00**	**2.0**<br>**10:00**	**3.0**<br>**11:00**	**4.0**<br>**12.00**	**6.0**<br>**14:00**	**9.0**<br>**17:00**	**24.0**<br>**(pre-dose)**<br>**8:00**
Day 1 and Day 2 plasma PK	3 mL	3 mL	3 mL	3 mL	3 mL	3 mL	3 mL	3 mL	3 mL
Day 15 (± 1) plasma PK			3 mL	3 mL		3 mL			
Day 29 (-1) plasma PK	3 mL								
Day 1 and Day 15 (± 1) intracellular PK	40 mL								

5. INTRODUCTION

Initial enthusiasm concerning the use of hydroxyurea and didanosine in the treatment of HIV disease centered upon the action of hydroxyurea to potentiate didanosine's antiviral activity and the ability of the combination to restore didanosine sensitivity in resistant viral populations. However, toxicities also emerged that resulted in a significant decline in the combination's use. Recent discoveries concerning unique mechanisms of action associated with the two-drug combination of didanosine and hydroxyurea (e.g., preventing immune system hyper-activation and blocking the CCR5 co-receptor needed for cellular entry with naturally-occurring β-chemokines), a better understanding of the factors that contributed to the combination's toxicity in early trials, and new insights into the dosing of each compound to reduce toxicity and increase efficacy have rekindled interest in this combination as part of a new generation of HIV treatment strategies.

5.1. Unique Mechanisms of Action to Address an Unmet Medical Need

Despite the advent of over twenty commercial agents in multiple classes, current anti-HIV regimens continue to target the virus only. Problems with long-term tolerability and the potential selection of resistant virus populations often necessitate numerous changes over the course of a patient's therapy. In order to effectively stop or slow the progression of HIV to acquired immunodeficiency syndrome (AIDS), therapies must go beyond simply reducing viral load, as antiretroviral drugs currently do, to protecting the immune system by reducing the chronic over-activation of the system associated with HIV-1 infection that is believed to result in the continuous decline in functioning CD4 cells over time.

"Virostatic" approaches to HIV therapy seek to combine the action of direct antivirals (e.g., nucleoside analogues) to reduce viral load with agents that are cytostatic – preventing the over-activation of the immune system postulated to be the cause of the progressive loss of both the number and functionality of CD4 cells that occurs over the natural course of the infection.

Current therapeutic approaches to reduce the replicative capacity of HIV-1 attempt to drive down viral replication by acting upon multiple steps in the natural life-cycle of the virus. A more complete understanding of the influence of chronic immune activation in the presence of HIV disease has opened another route for controlling

viral replication. Elucidation of the co-receptors necessary for HIV-1 viral entry – CCR5 and CXCR4 – have also resulted in new strategies and agents to block this critical first step in the continuation of HIV infection. In addition to these mechanistic considerations, therapeutic approaches that incorporate multiple mechanisms of action while reducing pill burden, cost of therapy and toxicity are needed to address the global pandemic.

The virostatic combination of hydroxyurea (HU) and didanosine (ddI) has been identified as a candidate virostatic regimen to address this unmet medical need. The two agents, each of which have been employed in the clinical setting for many years, have been formulated together in a fixed dose combination (FDC) product known as VS411. Beneficial synergies between the drugs' mechanisms of action, combined with HU's immunomodulating properties, the activity of hydroxyurea to induce a natural blockade of the CCR5 co-receptor, and the combination's favorable resistance profile, allow the investigation of lower doses of each drug than were previously prescribed, potentially minimizing the complexity, toxicity and cost of treatment while maintaining, or even improving, therapeutic efficacy.

5.2. A Better Understanding of the Factors that Contributed to the Toxicity of Hydroxyurea and Didanosine

Based upon observations in clinical trials conducted in the late 1990s, the combination of hydroxyurea and didanosine is perceived by many to be too toxic for clinical use. Indeed, when used in combination with stavudine (Zerit® - d4T) and with the relatively high doses of hydroxyurea and didanosine used at that time, the combination appeared to increase both the incidence and severity of nucleoside toxicity. In fact, the reason most often cited for discontinuing the combination, both in clinical trials and in practice, was peripheral toxicity. More serious toxicities, including several cases of fatal pancreatitis, were also seen when high doses of hydroxyurea were administered with full-dose didanosine and stavudine.

There were two cases of fatal pancreatitis in the ACTG 5025 trial. In this study, subjects who were maximally suppressed on an AZT-3TC-based HAART regimen were randomized to continue the regimen or to switch to stavudine-didanosine-indinavir with or without high-dose hydroxyurea (1,200 mg). Although two cases of fatal pancreatitis occurred on the high-dose hydroxyurea arm, the overall incidence of

treatment-related pancreatic toxicity was the same for both stavudine-didanosine-indinavir arms, with or without high-dose hydroxyurea. In a subsequent meta-analysis of ACTG trials, the regimen with the highest risk of pancreatitis was stavudine-didanosine-indinavir and the risk was independent of the addition of hydroxyurea. The authors concluded that hydroxyurea does not increase the risk of pancreatitis compared to didanosine alone.

Hematological toxicities were also reported in trials investigating high doses of hydroxyurea (> 1,000 mg/day). In the ACTG 307 trial, cases of anemia, thrombocytopenia and neutropenia were reported, but almost exclusively in the arm receiving the highest dose of hydroxyurea (1,500 mg).

Today, there is a better understanding of the factors that led to the high rates of toxicity reported in early clinical trials of the hydroxyurea-didanosine combination. These factors, each of which is avoidable, include:

5.2.1. Combination with Stavudine (Zerit® - d4T)

The toxicity of the stavudine-didanosine dual nucleoside combination is well appreciated and the combination is no longer recommended for the treatment of HIV disease. The addition of hydroxyurea (most often at high doses of >1,000 mg/day) was observed to potentiate both the antiviral activity and the toxicity of the combination. It is now known that intracellular concentrations of d4T-triphosphate are elevated in the presence of hydroxyurea due to increased activity of thymidine kinase – a fact not widely recognized at the time. Increased intracellular d4T-TP concentrations would be expected to yield both increased antiviral activity and increased toxicity.

5.2.2. Lack of Weight-based Dosing Adjustments for Stavudine and Didanosine

The labeling for both stavudine and didanosine calls for reducing the dose of each agent in patients weighing less than 60 kg. The recommended daily dose of didanosine is reduced to 250 mg (from 400 mg) and the dose of stavudine is reduced to 30 mg b.i.d. (from 40 mg b.i.d.) in such patients. Case reviews of patients developing pancreatitis toxicity revealed that these dose reductions were not always

made in patients weighing less than 60 kg. The potentiation of nucleoside toxicity by high dose hydroxyurea may have further compounded this situation.

5.2.3. Higher Didanosine Cmax as QD Tablets Replaced BID

In an attempt to increase patient convenience, the dosing of didanosine underwent a change from 200 mg b.i.d. to 400 mg q.d. (in patients weighing > 60 kg), including its use in regimens containing hydroxyurea. Prior to the introduction of enterically-coated didanosine, this was accomplished through the use of Videx® chewable tablets. As these tablets were immediate release, the resulting Cmax when taking 400 mg q.d. was in excess of that seen when the agent was administered in two divided doses. To avoid this effect, VIROSTATICS has developed a proprietary enteric-coated didanosine for inclusion in the VS411 FDC product to give a lower Cmax while maintaining total drug exposure over the full 24-hour dosing period.

5.2.4. Increasing Doses of Hydroxyurea

During the late 1990s, the dose of hydroxyurea was being raised in an attempt to further enhance the antiviral activity of the HU-ddI combination. Initially dosed at 500 mg b.i.d., doses were increased to 1,000 mg q.d. (with higher a resulting Cmax), 1,200 mg q.d., and 1,500 mg (or higher) q.d. Paradoxically, the efficacy of the combination was reduced at higher doses of hydroxyurea due to an increase in toxicity (decreased tolerability) and the fact that the agent becomes cytotoxic (i.e., no longer cytostatic) at higher doses.

5.2.5. Didanosine Potentiation of Hydroxyurea

Findings both in vitro and in vivo now suggest that, through an as yet unidentified mechanism of action, didanosine actually potentiates the ability of hydroxyurea to decrease the intracellular concentration of dATP – the natural deoxynucleotide (dNTP) with which didanosine's active metabolite (ddATP) competes. Although not widely recognized at the time, this potentiation would have been occurring in addition to the higher doses of hydroxyurea that were being investigated, potentially adding to the toxicity of the combination.

5.2.6. Prior History of Pancreatitis and Elevated Markers

In reviewing reported cases of pancreatic toxicity in patients receiving hydroxyurea and didanosine, it has been observed that a number of the patients had a previous history of pancreatitis or elevated pancreatic markers during therapy. In the development of VS411, subjects will be screened for prior pancreatic disease and stringently monitored while on therapy.

5.2.7. VS411 – Dosing Based Upon New Understandings

Historically, the clinical experience and research with of the hydroxyurea-didanosine combination can be divided into two phases. In the first phase, the addition of HU to ddI monotherapy, mostly in antiretroviral naïve (or not heavily pre-exposed to drugs) patients, resulted in increased HIV suppression without increased toxicity. When stavudine was added to the regimen, greater efficacy was seen, but this was accompanied by increased toxicity as well.

In the second phase, hydroxyurea was added to three or four drug HAART regimens, often in the salvage situation and almost exclusively containing stavudine. The dose of hydroxyurea was increased up to 1,500 mg total daily dose, and didanosine was often used once instead of twice a day (thus increasing ddI Cmax). Hydroxyurea was often added to fully suppressive regimens, but little additional efficacy was seen (most regimens were already maximally suppressive) while toxicities often became apparent as most studies were performed with relatively high doses of 1000 to 1500 mg of HU and 400 mg of ddI per day.

In the RIGHT 702 study it was shown that a lower hydroxyurea dose of 600 mg q.d. achieved better antiretroviral activity than did higher doses, together with better CD4+ cell count increases and fewer adverse effects. The 600 mg q.d. dose is thought to represent the maximum cytostatic effect of hydroxyurea with higher doses becoming cytotoxic. These results form the basis of a new phase in the clinical development of the hydroxyurea-didanosine combination. By taking advantage of the synergistic interaction between hydroxyurea and didanosine, the development program of VS411 will explore combinations of even lower dose levels of didanosine and hydroxyurea (range: 200 to 400 mg ddI and 300 to 600 mg HU) in an attempt to lower toxicity while maintaining or improving clinical efficacy.

VS411 has been developed by RIGHT – the parent company of VIROSTATICS S.R.L. – to modify the release profile and, thereby, the plasma concentration-time profile of didanosine to potentially reduce the toxicity of the drug. The dosage of hydroxyurea has also been lowered to reduce toxicity while maintaining its cytostatic properties and maximizing its antiviral activity. VS411 incorporates small tablets of HU (150 mg each) and ddI (100 mg each) into capsules, allowing investigation of numerous dosage combinations of the two agents. VS411 will be developed with a contraindication to use with stavudine and in patients with a previous history of pancreatitis.

A review of the literature revealed the earliest reported appearance of the L74V mutation at five months. No reference was found that directly addressed the question of short-term exposure to didanosine. Researchers at RIGHT subsequently identified properly stored samples representing baseline and Week 4 from 17 HIV-1-infected subjects (both naïve and nucleoside experienced) receiving either didanosine monotherapy (n = 7) or hydroxyurea/didanosine (n = 10). A Full Mutations Report was generated by Virco for paired baseline and Week 3 (n = 1) or Week 4 (n = 16) samples for each subject. The results were analyzed with respect to changes occuring between baseline and Week 4. It appeared that the exposure of subjects to a four-week course of either didanosine monotherapy or hydroxyurea/didanosine combination therapy was not associated with the selection of mutations known to confer clinically-significant resistance to didanosine.

6. STUDY OBJECTIVES AND PURPOSE

6.1. Objectives

To characterize different dose combinations of hydroxyurea plus didanosine in the form of VS411 for further evaluation in phase IIb studies with respect to:

- HIV-1 antiviral activity;
- Pharmacokinetics;
- Pharmacokinetic and pharmacodynamic relationships;
- Quantification of intracellular ddATP/ dATP;
- Immunological parameters;
- Safety / tolerability;

- Evaluation of genotypic resistance to nucleoside/ nucleotide analogues at baseline and after four weeks of treatment.

7. STUDY POPULATION

7.1. Number and type of subjects

A total of 60 subjects will be enrolled in five study arms (i.e., 12 subjects per study arm). All patients that satisfy the following inclusion and exclusion criteria are eligible.

7.2. Inclusion criteria

1. Provide written informed consent;
2. Chronical HIV-1 infection, documented by licensed HIV antibody ELISA and confirmed either by Western blot, positive HIV blood culture, positive HIV serum antigen or plasma HIV-1 viremia;
3. HIV-1 antiretroviral treatment-naïve;
4. Plasma HIV-1 RNA levels greater than 5000 copies/mL;
5. $CD4^+$ T-cell counts greater than 250 cells/mm^3;
6. CDC AIDS Surveillance Case Definition classification stage A or B;
7. At least 18 years of age;
8. Males or non-pregnant, non-lactating females;
9. Use of adequate and reliable forms of contraception during the study (males and females of childbearing potential) and starting at least one month prior to study drug administration for females and for 30 days after discontinuation of study medication.

7.3. Exclusion criteria

1. HIV-2 co-infection;
2. Primary (acute) HIV-1 infection;
3. Less than 60 kg body weight;
4. Clinically relevant laboratory abnormalities: anemia (Hb < 10 g/dL, thrombocytopenia (platelets < 100,000 per mm^3), leucopenia (WBC < 3,000 per mm^3), elevated triglycerides (> 2 times the ULN), elevated liver transaminases (> 2 times the ULN), elevated bilirubin (> 2 times the ULN), elevated amylase (> 2 times the ULN), elevated lipase (> 2 times the ULN), decreased estimated creatinine clearance (< 60 mL/min) using the Cockcroft-Gault formula;

5. Use of co-medication with a known clinically significant pharmacological interaction with one or more of the study drugs. Patients using H2 blockers should agree <u>not</u> to use these drugs within 24 hours prior to and during PK sampling. Patients using oral antacids should agree <u>not</u> to use these drugs within 12 hours prior to and during PK sampling. Use of proton pump inhibitors are not allowed;
6. Active alcohol or drug abuse (methadone use is allowed);
7. Anticipated non-compliance with the protocol;
8. Anticipated need to start HAART within the study period;
9. Presence of a newly (within 30 days) diagnosed HIV-related opportunistic infection or condition requiring acute therapy at the time of enrolment;
10. Patients who have taken any investigational drug 30 days prior to the start of the study;
11. Chronic active viral hepatitis B or hepatitis C, confirmed by positive serology at screening determined by enzyme-linked immunosorbent assay (ELISA). Chronic active hepatitis B is defined as the presence of HBsurface-antigen and/or HBe-antigen. Chronic hepatitis C is defined as the presence of antibodies against HCV.
12. Current or former pancreatitis;
13. Pregnancy;
14. History of, or currently receiving interferon therapy;
15. Presence of didanosine-associated resistance mutations;
16. Current or recent (within the prior 3 months) use of immunodulatory agents (including vaccines);
17. Current or recent (within the prior 3 months) use of ribavarin.
18. Known allergy to the active or inactive components of VS411.

7.4. Withdrawal and replacement criteria

A subject will prematurely discontinue **from the study** if:

- The subject withdraws informed consent;
- The subject is lost to follow up;
- The subject dies.

Subjects can voluntary leave the study at any time for any reason if they wish to do so without any consequences.

A subject will prematurely **stop study treatment but continue in the study if**:

- The subject experiences a grade 4 adverse event (AE)/ toxicity;
- The subject experiences a grade 3 or higher acute systemic allergic reaction;
- The subject develops clinical pancreatitis;
- The subject develops clinical hepatotoxicity;
- The subject develops grade 2 or higher peripheral neuropathy;
- The subject shows a significant decrease in creatinine clearance (Cockcroft-Gault) which in the opinion of the investigator is clinically relevant;
- The subject experiences treatment-related AEs, which in the opinion of the treating physician are an indication to discontinue any of the study drugs;
- If there is a need to start concomitant treatment with medication that is not compatible with the use of the study medications (didanosine and/or hydroxyurea);
- The $CD4^+$ T cell percentage of a subject decreases 20% or more measured at least two weeks apart and the subject has to start HAART based upon the local guidelines for start of anti retroviral treatment;
- A female participant becomes pregnant.

All subjects who permanently discontinue study medication prior to completion will have a complete set of clinical and laboratory evaluations in line with the evaluation schedule until Week 6 (see Section 4) to follow up safety and to perform the intention-to-treat analysis. PK assessments do not have to be performed after the patient discontinues study drug. Patients are allowed to start HAART after discontinuation of study medication whenever needed.

The only reasons for not performing any of the assessments according to the flow chart in Section 4 are subjects' withdrawal of informed consent, loss to follow up, or death of the subject. In all other circumstances, the utmost should be done to perform the assessments according to the flow chart in Section 4. However, after permanent discontinuation of study medication, further PK assessments will not be made.

Twelve subjects will be included per study arm. Only subjects who drop out after screening but before randomisation will be replaced. Subjects who drop out after randomisation but before intake of the first study drug will not be replaced. In case five subjects or more drop out after randomisation but before intake of the first study drug, five new subjects will be included and randomized. It is important to minimize the time between randomisation and the baseline visit (in particular, intake of study medication) as much as possible. The goal is to have at least 10 evaluable subjects per treatment arm.

8. STUDY DESIGN

8.1. Overall design

This is a randomized, 5-arm, double-blind phase IIa multicentre study to identify the optimal dosing of hydroxyurea plus enterically-coated didanosine in the treatment of HIV infection.

Subjects will be treated with the study medication for 28 days. Post-dosing follow-up will continue for another 14 days to gather data on viral rebound and additional safety data.

Subjects are randomized in a 1:1:1:1:1 ratio to one of 5 study arms:
- Didanosine 400 mg QD plus hydroxyurea 600 mg QD
- Didanosine 200 mg QD plus hydroxyurea 600 mg QD
- Didanosine 200 mg QD plus hydroxyurea 300 mg QD
- Didanosine 400 mg QD plus hydroxyurea 300 mg QD
- Didanosine 200 mg QD plus hydroxyurea 900 mg QD

An integrated dose-response and exposure-response analysis applying advanced pharmacokinetic/pharmacodynamic (PK/PD) modelling will be employed to select the optimal dose combination of didanosine and hydroxyurea for use in future long-term studies.

8.2. Rationale for study design

8.2.1. Endpoints and study duration

HIV viral load is generally accepted as the objective parameter best utilized to measure antiviral activity. The study is blinded with regard to this and other parameters to avoid any potential bias in activity assessment. As shown in many similar phase IIa dose-ranging HIV trials and due to its accuracy, selectivity and sensitivity, the anti-viral activity as measured by reduction of HIV viral load over time has proven to be a reliable quantitative parameter for the accurate selection of doses and patient numbers in both pilot and long-term dose-finding trials. Since a wide range of doses and combinations of hydroxyurea and didanosine are being studied, the planned analyses and the application of advanced PK/PD modelling techniques will further support dose selection for future studies.

The 4-week study duration and subsequent post-dosing follow-up allows for an optimal assessment of the short-term safety and tolerability profile of the investigated regimens. This period of 'virtual' monotherapy (i.e., one approved anti-HIV medication) is considered necessary for adequate differentiation of antiviral activity between the five dosage combinations while minimizing the risk of emergent didanosine resistance. A recent analysis of subject samples from previous trials did not reveal any cases in which resistance emerged when the hydroxyurea/didanosine combination, or didanosine alone, was administered for four weeks (see section 5.2.7 and/or the IB). Post-dosing follow-up will continue for another 14 days to gather data on viral rebound and additional safety data.

The antiviral activity will be related to the plasma concentrations of the two components of VS411. Therefore extensive plasma sampling is required.

Endpoints are:

- Changes from baseline in the log-transformed plasma HIV-1 RNA concentrations;
- Plasma PK/PD of didanosine and hydroxyurea (didanosine also intracellularly);
- Quantification of intracellular ddATP/ dATP
- Changes in $CD4^{+}$ T-cell counts;
- Incidence and severity of adverse events;

- Evaluation of genotypic resistance to nucleoside/ nucleotide analogues at baseline and after 28 days treatment

8.2.2. Dosing rationale

From a patient-management perspective, it is preferable to dose anti-HIV medications once-daily. The RIGHT 702 study has shown that QD dosing of hydroxyurea in combination with didanosine provides efficacy equivalent to administering the same daily doses given twice daily. The RIGHT 702 study also demonstrated that a 600 mg daily dose of hydroxyurea (combined with a 400 mg daily dose of didanosine) demonstrated better antiviral activity than did higher HU doses, together with a greater CD4+ T-cell count increase and fewer adverse events. Therefore, 600 mg hydroxyurea was selected as the highest HU dose to be studied in combination with 400 mg ddI.

In vitro, it has been shown that hydroxyurea can decrease the IC90 of didanosine by up to six-fold. As it is accepted that tolerability/toxicity is often associated with increasing drug exposure (i.e., drug dose), the goal of pharmaceutical product development is to establish the lowest effective dose in order to minimize toxicity. Therefore it is prudent to also test lower doses of didanosine when used in combination with HU. This study will test didanosine at 200 mg -- one-half of the traditional daily dose yet still expected to exert sufficient anti-HIV activity in light of the HU-induced six-fold reduction in IC90. Two hundred milligrams of didanosine will be paired with 300, 600, or 900 mgs of hydroxyurea. The 900 mg HU dose is being studied to determine if there is an anti-viral effect when providing additional HU exposure in combination with the reduction in didanosine exposure. These three hydroxyurea doses are all less than the 1,000 mg per day often administered in early trials of the combination.

Historically, an increase in didanosine tolerability issues was associated with the implementation of QD dosing using the immediate release, chewable tablets. Originally approved as a twice-a-day drug based upon didanosine's plasma half-life, the drug was administered as 200 mg given twice daily (125 mg BID for patients weighing less than 60 kg). As the intracellular half-life of dideoxyadenosine triphosphate (the active didanosine moiety) would support once-daily dosing, practitioners began utilizing didanosine at 400 mg once-daily (250 mg QD for

subjects weighing less than 60 kg) using the immediate release chewable tablets. As it has been suggested that greater Cmax plasma concentrations of didanosine may have been related to increased side effects, the originator company developed an enterically-coated didanosine product to alter the PK profile and decrease toxicities associated with the high level of "buffer" contained in four 100 mg didanosine tablets. VS411 has been formulated with a novel proprietary enterically-coated didanosine designed to further decrease the peak (C_{max}) exposure while still maintaining the overall exposure (AUC) as the originator product when given once-a-day. With a lower Cmax, the proprietary didanosine formulation incorporated into VS411 would be expected to result in improved tolerability.

8.2.3. Sample size rationale

Due to the exploratory nature and short duration of the study, no measurable clinical benefit is to be expected. As the trial then need not be powered to detect such a clinical effect, no formal sample size calculation was performed. A sample size of 12 subjects per arm (total n = 60) was chosen for this study as sample sizes in this range are commonly selected for these types of studies in HIV. These sample sizes are considered adequate for exploratory pharmacokinetic and first-in-man trials, in which minimal volunteer numbers are selected for safety reasons, while the outcomes provide sufficient guidance for future development steps. Results from similarly sized phase IIa studies have been used to design pivotal confirmatory trials for which formal sample size calculations are required to demonstrate adequate safety and efficacy for regulatory approvals. These early exploratory studies serve as input for sample size calculations for pivotal studies while the outcomes themselves only serve as supportive evidence in future regulatory filings.

8.3. Randomization, blinding and treatment allocation

Assignment to treatment arm will be done using the randomization scheme prepared by the Biometrics department of IATEC. Randomization will ensure a random and equal distribution of the subjects over the treatment arms. Subjects will be assigned to a treatment arm and PK-sequence prior to first dose intake. The time period between randomization and intake of first study drug should be kept as short as possible. Subjects will only be assigned to a treatment once the subject has passed the screening. At randomization subjects will be assigned to a randomization number and to PK-sequence A or B (see flow chart in section 4) by the Biometrics

department of IATEC. Both subjects and observers are blinded for the dosage of VS411 supplied to the subjects. The PK-sequence will not be blinded

Both subjects and observers are blinded for the dosage of VS411 supplied to the subjects. Therefore, all study medication drugs will be packed in an identical way. The packages will contain identical capsules containing different doses of VS411. In addition, an emergency unblinding of the treatment code is available (See Paragraph 11.8). Deblinding of all patients will occur after database lock. However, in case an adverse event occurs for which it is necessary in the opinion of the investigator to unblind this individual subject an Internal Safety Board, consisting of medical representatives of Virostatics and IATEC and the Principal Investigator from the site where the patient is treated, will review the blinded data of the affected subject and give an advice whether to unblind the data. This Internal Safety Board can be contacted via the Medical Monitor of the study (Phone +31 (0)20 3149375). Based upon this advice, the investigator decides whether it is necessary to unblind this individual subject. In case unblinding is necessary the investigator may open the randomisation envelope according to the procedure as described in section section 11.8.

9. Investigational product

The study medication is VS411, a capsule containing different dose combinations of hydroxyurea and didanosine. Each capsule, made of hard gelatin, contains 4 tablets: 2 tablets of 100 mg ddI or matching placebo and 2 tablets of 150 mg HU or matching placebo. The inactive ingredients of the HU tablet are microcrystalline cellulose, anhydrous lactose, croscarmellose sodium, pregelatinized corn starch, and glyceryl behenate. The inactive ingredients of the ddI tablet are anhydrous lactose, pregelatinized corn starch, croscarmellose sodium, talc, hypromellose, glyceryl behenate, opadry, and acryl-eze.

9.1. Study Medication

Subjects will receive three (3) capsules containing different combinations of hydroxyurea, didanosine and matching placebo in QD doses according to randomization. From Visit 2 through Visit 6 the amount of study medication needed until the next visit will be distributed to subjects. The study medication has to be

taken on an empty stomach in the morning, orally, without food, and at least 2 hours before breakfast. For further information on product characteristics, preclinical or clinical data of the combination of ddI and HU, please consult the Investigator's Brochure (IB).

9.2. *Packaging and labeling*

The study medication will be prepared, packed and labeled by its manufacturer in accordance with Good Manufacturing Practice (GMP) and applicable local laws and regulations.

The study medication will be packed per day in small PE containers, labeled in compliance with GMP, Annex 13.

9.3. *Handling and storage*

The study medication will be in capsule form and must be stored in a dry environment at ambient temperature below 25°C. Temporary temperature excursions are allowed. The study medication will be shipped to the study site under controlled conditions and will be stored as described above. The responsible pharmacist/ investigator will ensure a temperature surveillance system and study medication inventory.

9.4. *Responsibilities*

In compliance with ICH GCP, the responsibility for the study medication at the study site lies with the investigator and includes the following:

- Documented receipt of the study medication;
- Deliveries are documented;
- Study medication is adequately stored;
- Records should include dates, quantities and the unique code numbers assigned to the study medication and trial subjects;
- Documentation should include dispensing of the study medication to subjects as specified by the protocol;
- Reconciliation of the study medication

The following guidelines apply:

- The investigator agrees not to supply any study medication to any person except to the subjects included in the study;
- The investigator/ pharmacist will keep the study medication in a locked secure storage facility, only accessible to authorized staff;
- A study medication inventory will be maintained, including materials received and dispensed to subjects;
- At the conclusion or termination of the study, the investigator/pharmacist agrees to conduct a final drug supply inventory and to record this on a Drug Accountability Form.

9.5. Accountability

Drug accountability includes records of the study medication dispensed by the investigator to the subjects, and records of the unused study medication/ empty blisters that have to be returned to the investigator by the subject. The Drug Accountability Form will be verified by the monitor on a regular basis. Drug reconciliation check will be done by the monitor after visit 8.

10. STUDY CONDUCT

10.1. Study procedures at each visit

An overview of all study procedures can be found in the overviews at page 15

10.1.1. Screening visit (visit 1)

A screening assessment for eligibility will be performed between Day -28 and Day -7. Written informed consent must be obtained prior to the initiation of any study related intervention. Screening procedures include:

- Demographic information: date of birth, gender, ethnic group;
- History of HIV-infection: mode of infection, date of last negative and first positive HIV-1 test, nadir $CD4^+$ T-cell count , CDC classification);
- Medical history: all current medical conditions, relevant medical history;
- Medication history: all medication up to three months prior to inclusion, all current medication;

- A targeted physical examination;
- Height;
- Body weight;
- Beta-HCG: female subjects of childbearing potential only;
- Laboratory assessments: hematology and clinical chemistry (see paragraph 10.2), lymphocyte subsets (absolute and percentage $CD4^+$ and $CD8^+$ T-cell counts), plasma HIV-1 RNA (local assessment), hepatitis serology[*]
- Two blood samples of 4.5 mL will be drawn for genotypic resistance and for general storage of plasma (Two EDTA samples);
- One blood sample of 30 mL will be drawn for storage of viable PBMC for extensive immunology assessments;

Total amount of blood drawn: 59.5 mL

10.1.2. Baseline and follow up visits

Baseline visit (Visit 2)

Patients will be randomized between screening and Day 1. At Day 1, the subjects will come to after an overnight fast (no food intake since midnight) **and before intake of VS411**. The following baseline values will be assessed:

- A full physical examination;
- Body weight;
- Beta-hCG: female subjects of childbearing potential;
- Assessment of possible HIV associated conditions and the occurrence and severity of clinical adverse events: these events include severity, start dates, stop dates and relation to study medication;
- Use of concomitant medication;
- Laboratory assessments: hematology and clinical chemistry, lactate (sample should preferably be taken from patient in rest and not earlier than 30 minutes after heavy physical exercise, if the latter is applicable), lymphocyte subsets, storage of 7.0 mL EDTA blood for central assessment of plasma HIV-1 RNA;
- Two blood samples of 4.5 mL will be drawn one for genotypic resistance and one for general storage of plasma (Two EDTA samples);

[*] HBs antigen, HBs antibodies, HBe antigen, HBe antibodies, HBc antibodies, HCV antibodies

- In the 30 subjects of group A, three blood draws of 3 mL will be performed (1, 2 and 4 hours after intake of the study drug) (See detailed schedule at page 17); In 30 subjects of group B, nine blood draws of 3 mL will be performed at set time points for PK/PD analyses. The first blood sample will be taken after the baseline assessments, within 0.5 hours before intake of the first dose of the study drug. The intake of study medication will be observed by study staff. The last blood draw will be **before intake of the next dose of VS411**.
- One blood sample of 40 mL will be drawn for storage of PBMCs to assess intracellular concentrations of ddI **within 0.5 h before** intake of the study medication in all subjects;
- Distribution of study medication;
- Subjects start VS411 at the doses as randomized.

Total amount of blood drawn: 76 mL (Group A) or 91 mL (Group B).

Day 16 (group A) and Day 2 (group B) (Visit 6 + 1 day and 2 + 1 day):

At Day 16 (visit 6 + 1 day), subjects of group A (the subjects who had a PK evaluation at visit 6 (day 15)) have to come back early in the morning, after an overnight fast for a bloodsample of 3 mL for the 24 hours PK assessment **before intake of the next dose of VS411**.

Likewise, at Day 2 (visit 2 + 1 day), subjects of group B (the subjects who had a PK evaluation at Day 1 (visit 2)) have to come back early in the morning for a blood sample of 3 mL for the 24 hours PK assessment **but before intake of the next dose of VS411**.

Total amount of blood drawn per visit: 3 mL

Day 3 and Day 5 (Visit 3 and 4)

At Day 3 and Day 5 the following procedures will be performed:

- Assessment of possible HIV associated conditions and the occurrence and severity of clinical adverse events: these events include severity, start dates, stop dates and relation to study medication;
- Use of concomitant medication;
- A targeted physical exam will only be performed in case of adverse events and/or clinically relevant signs and symptoms, upon discretion of the physician;

- Laboratory assessments: hematology and clinical chemistry, lymphocyte subsets, storage of 7.0 mL EDTA blood for central assessment of plasma HIV-1 RNA;
- One blood sample of 4.5 mL will be drawn for general storage of plasma (one EDTA);
- Distribution of study medication

Total amount of blood drawn per visit: 20.5 mL.

<u>Day 8 (Visit 5)</u>

At Day 8 the following procedures will be performed:

- Full physical examination;
- Assessment of possible HIV associated conditions and the occurrence and severity of clinical adverse events: these events include severity, start dates, stop dates and relation to study medication;
- Use of concomitant medication;
- Laboratory assessments: hematology and clinical chemistry, lymphocyte subsets, storage of 7.0 mL EDTA blood for central assessment of plasma HIV-1 RNA;
- One blood sample of 4.5 mL will be drawn for general storage of plasma (one EDTA);
- Distribution of study medication

Total amount of blood drawn: 20.5 mL.

<u>Day 15 (Visit 6)</u>

At Day 15 the subjects need to come to the clinic after an overnight fast, **<u>and before taking the dose of VS411</u>** for that day. The following procedures will be performed:

- Body weight;
- Full physical examination;
- Assessment of possible HIV associated conditions and the occurrence and severity of clinical adverse events: these events include severity, start dates, stop dates and relation to study medication;
- Use of concomitant medication;
- Laboratory assessments: hematology and clinical chemistry, lymphocyte subsets, storage of 7.0 mL EDTA blood for central assessment of plasma HIV-1 RNA;

- One blood sample of 4.5 mL will be drawn for general storage of plasma (one EDTA).
- In 30 subjects of Group A, nine blood draws of 3 mL will be performed at set time points for PK/PD analyses. The first blood sample will be taken after the baseline assessments, within 0.5 hours before intake of the first dose of the study drug. The intake of study medication will be observed by study staff. The last blood draw will be **before intake of the next dose of VS411**; in the 30 subjects of Group B, three blood draws of 3 mL will be performed (1, 2 and 4 hours after intake of the study drug) (See detailed schedule at page 17);
- One blood sample of 40 mL will be drawn for storage of PBMCs to assess intracellular concentrations of ddl **within 0.5 h before** intake of the study medication in all subjects;
- Distribution of study medication

Total amount of blood drawn: 84.5 mL (group A) or 69.5 mL (group B).

Day 29 (Visit 7)

At Day 29 the subjects will come to the clinic **before taking the dose of VS411 for that day** where the following procedures will be performed:
- Beta-hCG: female subjects of childbearing portential;
- Body weight;
- Full physical examination;
- Assessment of possible HIV associated conditions and the occurrence and severity of clinical adverse events: these events include severity, start dates, stop dates and relation to study medication;
- Use of concomitant medication;
- Laboratory assessments: hematology and clinical chemistry, lactate (sample should preferably be taken from patient in rest and not earlier than 30 minutes after heavy physical exercise, if the latter is applicable), lymphocyte subsets, storage of 7.0 mL EDTA blood for central assessment of plasma HIV-1 RNA;
- Two blood samples of 4.5 mL will be drawn one for genotypic resistance and one for general storage of plasma (two EDTA samples).
- One blood sample of 30 mL will be drawn for storage of viable PBMC for extensive immunology assessments;

- A bloodsample of 3 mL will be taken for the 24 hours PK assessment **before intake of the next dose of VS411**.

Total amount of blood drawn: 60 mL.

Day 43 (Visit 8)

At Day 43 the following procedures will be performed:

- Full physical examination;
- Assessment of possible HIV associated conditions and the occurrence and severity of clinical adverse events: these events include severity, start dates, stop dates and relation to study medication;
- Use of concomitant medication;
- Laboratory assessments: hematology and clinical chemistry, lymphocyte subsets;
- One blood sample of 4.5 mL will be drawn for general storage of plasma (one EDTA)

Total amount of blood drawn: 13.5 mL.

10.2. Assessment of safety

- **Body weight:** (Visit 1, 2, 6, and 7).

- **Physical examination:** general appearance, eyes, ears/ nose/ throat, cardiovascular, respiratory, gastrointestinal, neurological, musculoskeletal, skin, other (Visit 2, 5, 6, 7, 8, during the other visits a targeted physical exam will be performed only in case of adverse events and/or clinically relevant signs and symptoms, upon discretion of the physician).

- **Clinical symptoms**: current medical conditions, HIV-associated conditions, (S)AEs and SUSARs at all visits, including concominant medication reporting.

- **Safety laboratory:** Safety laboratory assessments will be executed locally.
  - Hematology: hemoglobin, MCV, leukocytes + differential, thrombocytes (4.5 mL EDTA) (Visit 1, 2, 3, 4, 5, 6, 7, 8);

- Chemistry: creatinine, ASAT, ALAT, alkaline phosphatase, γGT, total and direct bilirubin, amylase, (in case amylase > 2 times the ULN then pancreatic-amylase needs to be tested), lipase, CPK, glucose, cholesterol, triglycerides (4.5 mL heparine) (Visit 1, 2, 3, 4, 5, 6, 7, 8);
- Lactate (2 mL sodium fluoride tube) (Visit 2 and 7).

▪ **Beta-hCG:** in urine, female subjects of childbearing potential only (Visit 1, 2 and 7).

▪ **Immunology**:
  - Lymphocyte subsets, $CD4^+$ and $CD8^+$ T- cell counts and percentages. These data will be retrieved from the samples taken for the hematology assessments (Visit 1, 2, 3, 4, 5, 6, 7, 8);
  - Viable PBMC's will be stored for more extensive and special immunology tests in particular cytostatic markers by using "state of the art" methods (30 mL EDTA) (Visit 1, 7).

▪ **Virology**:
  - Plasma HIV-1 RNA. Except for the screening visit where HIV-1 RNA will be assessed immediately, plasma will be stored for central assessment of plasma HIV-1 RNA. It might be considered to perform also HIV-1 subtyping/genotyping on stored plasma. (7.0 mL EDTA) (Visit 1, 2, 3, 4, 5, 6, 7).

▪ **Resistance testing**:
  - Plasma will be stored for central resistance testing (4.5 mL EDTA) (Visit 1, 2, 7).

The procedures for eliciting reports of and for recording of AEs are described in Paragraph 11.

In case of an AE subjects will be followed until the adverse event has been resolved or stabilized (up to two months after last study visit). Safety data of subjects who have withdrawn informed consent will be collected according to the protocol where possible.

10.3. Assessments of efficacy

- **Virology**:
  - Plasma HIV-1 RNA levels to be done centrally on stored plasma (7.0 mL EDTA) (Visit 1, 2, 3, 4, 5, 6, 7);

- **Pharmacokinetics**:
  - PBMCs for assessment of intracellular ddATP and dATP (40 mL EDTA) (Visit 2 and 6).

10.4. Concomitant medication

Concomitant medication, including drugs used for the primary or secondary prophylaxis of opportunistic infections, may be continued for the duration of the study (excluding drugs with a known interaction with hydroxyurea or didanosine). All used medications must be documented.

11. ADVERSE EVENTS AND OTHER SAFETY ASPECTS

11.1. Definitions

ADVERSE EVENT (AE): An AE is any untoward medical occurrence in a patient or clinical investigation subject administered a pharmaceutical product and which does not necessarily have a causal relationship with this treatment. An AE can therefore be any unfavorable and unintended sign, symptom, or disease temporally associated with the use of a medicinal product, whether or not considered related to the medicinal product.

Pre-existing events, which increase in frequency or severity, or change in nature during or as a consequence of use of a drug in human clinical trials, will also be considered as AEs. Clinically significant laboratory abnormalities, as judged by the investigator, are considered Adverse Events and should be documented in the CRF. Adverse events will be classified according to the Division of AIDS (DAIDS) Table for Grading the Severity of Adult and Pediatric Adverse Events, Version 1.0, December, 2004 (see Appendix II).

Any overdose, whether or not associated with an adverse experience, is considered as an AE. An overdose is defined as any dose exceeding the maximum recommended dose according to the prescribing information.

ADVERSE EVENTS DO NOT INCLUDE:

- Medical or surgical procedures (e.g., surgery, endoscopy, tooth extraction, transfusion). The condition that leads to the procedure is an AE;
- Pre-existing diseases or conditions present or detected prior to start of study drug administration that do not worsen;
- Situations where an untoward medical occurrence has not occurred (e.g. hospitalization for elective surgery if known prior to start study, social and/or convenience admissions).

SERIOUS ADVERSE EVENT (SAE): An SAE is defined as any adverse experience regardless of causality that results in any of the following outcomes:

- Death;
- Life-threatening situation (patient is at **immediate** risk of death);
- Inpatient hospitalization or prolongation of existing hospitalization (excluding those for study therapy);
- Persistent or significant disability/incapacity;
- Congenital anomaly/birth defect in the offspring of a subject who received study drug;
- Other: emergency admissions and other medically significant events that may not result in death, be immediately life-threatening, or require hospitalization, may be considered a SAE when, based upon appropriate medical judgment, they may jeopardize the patient and may require medical or surgical intervention to prevent one of the outcomes listed in this definition.

CLARIFICATION OF SERIOUS ADVERSE EVENTS

Death is an outcome of an adverse event, and not an AE in itself. In reports of death due to "Disease Progression", where no other information is provided, the death will be assumed to have resulted from progression of the disease being treated with the study drug(s). Deaths must be reported for subjects on study and for deaths occurring within 6 months of last study evaluation, being the responsibility of the investigator.

"Occurring at any dose" does not imply that the subject is receiving study drug at the time of the event. Dosing may have been given as treatment cycles or interrupted temporarily prior to the onset of the SAE, but may have contributed to the event.
"Life-threatening," means that the subject was **at immediate risk** of death from the event as it occurred. This does not include an event that might have led to death, if it had occurred with greater severity.
Complications that occur during hospitalizations are AE's, but are not SAEs. If a complication prolongs hospitalization, it is a SAE.
"Inpatient hospitalization" means the subject has been formally admitted to a hospital for medical reasons, for any length of time. This may or may not be overnight. It does not include presentation and care within an emergency department, unless the event is judged as an "other important medical event" as per the definition of serious described above.
The investigator should attempt to establish a diagnosis of the event based on signs, symptoms and/or other clinical information. In such cases, the diagnosis should be documented as the AE and/or SAE and not the individual signs/symptoms.
Adverse events will be classified according to the table in Appendix II.

11.2. Suspected unexpected serious adverse reactions (SUSAR)

Adverse reactions are all untoward and unintended responses to an investigational product related to any dose administered (see definitions in Paragraph 11.1).
Unexpected adverse reactions are adverse reactions, of which the nature, or severity, is not consistent with the applicable product information (e.g. Investigator's Brochure for an unapproved IMP or Summary of Product Characteristics (SmPC) for an authorized medicinal product).

The SUSARs will be reported via a CIOMS form to the accredited METCs and the investigators involved in this study. The sponsor (or representatives) will also report (expedited) all Dutch domestic SUSARs to the CCMO. SUSARs that occur in other countries will be reported to the competent authority of the concerned country. The expedited reporting will occur not later than 15 days after the sponsor has first knowledge of the adverse reactions. For fatal or life threatening cases the term will be maximal 7 days for a preliminary report with another 8 days for completion of the report.

The non-domestic SUSARs will be reported quarterly to the competent authority and the accredited METC, as a line listing accompanied by a brief report highlighting the main points of concern.

11.3. Pregnancy notifications

The pregnancy notification form must be filled out and faxed to IATEC (+31 20 3149 394). This form will be part of the study documentation that the sites will receive before the first patient will be screened.

11.4. Annual safety report

The annual safety report may be combined with the annual progress report (see Paragraph 16.7).
In addition to the expedited reporting of SUSARs, the sponsor will submit, once a year throughout the clinical trial, a safety report to the accredited METC, competent authority, Medicine Evaluation Board and competent authorities of the concerned Member States.

11.5. Expedited reporting of SAEs and SUSARs

Initial notification

If the investigator identifies the occurrence of an SAE, an SAE report form must be completed and sent by fax to IATEC (+31 20 3149 394) within 24 hours of the investigator's knowledge of the event. This form will be part of the study documentation that the sites will receive before the first patient will be screened. Also fax copies of hospital case reports should be sent within 24 hours (i.e., hospital progress notes, results of applicable diagnostic tests, lab results, and biopsy results), autopsy reports, and other documents, when requested and applicable. See Section 2, for a listing of IATEC fax numbers.

Any fatal or life-threatening event should be reported immediately to IATEC by telephone. These preliminary reports will be followed within 24 hours by detailed descriptions that will include a completed SAE form, copies of hospital case reports, autopsy reports, and other documents, when requested and applicable. See Section 2, for a listing of IATEC telephone numbers.

Minimal information should include:

- **An identifiable subject or patient**
- **An identifiable reporting source**
- **All related adverse events**
- **All medications used including the suspect medicinal product**

The IATEC drug safety team will assess if the SAE should be regarded as a SUSAR. If so, they will report this on a CIOMS form to the relevant IEC and the competent authorities in the countries of the participating sites.

11.6. Follow-up of SAEs

Follow-up of SAEs and SUSARs that occur during the study will continue until satisfactory resolution or stabilization, with a maximum of six months, upon judgment of the investigator. The sponsor may request that certain adverse events to be followed until resolution.

If/ when supplementary information is available, a follow-up SAE Report Form must be completed by the site and faxed within 24 hours to IATEC.

Once faxed, the SAE form and accompanying documentation should be placed in the SAE section of the investigator's file. If supplementary information on a SAE has to be sent, the SAE form has to be used marked as "follow-up report".

Reporting Method

Faxing is the preferred method of reporting for SAEs. The SAE Report Form should be completed by the investigator or designee, and a copy faxed to IATEC within 24 hours. If a facsimile is not available, the investigator or designee should telephone or email IATEC to report the SAE and follow-up with a written report as soon as possible.

11.7. Emergency procedures and management of overdose

If a participating subject would receive more than one dosage at a certain time point, or more dosages than prescribed by the protocol, this will have to be documented as an overdose. Standard safety procedures should be applied.

11.8. Emergency breaking of treatment code

This study is blinded for the dosage of VS411 and therefore an emergency breaking of the treatment code is applicable. The principal investigator will be supplied with sealed envelopes per subject, containing the treatment allocation. Receipt of these envelopes will be confirmed in writing.The principal investigator informs the whole study team about the plan of storage of, and 24-hour access to these envelopes (hospital's pharmacy is preferable location). These agreements will be confirmed in writing in the initation visit report by the monitor, at the beginning of the trial. Only a medical emergency will allow the investigator to open the envelope and breaking the code only of the subject concerned. When an envelope is opened, date, time, and reason will be documented. Before the randomization of a subject is unblinded by the investigator, the investigator must contact the IATEC Study Medical Monitor by telephone at +31 (0)20 3149375.

12. STATISTICS

12.1. Statistical methods

Data will be described by means and standard deviations or median and interquartile ranges, whichever is applicable. The changes from baseline in log-transformed plasma HIV-1 RNA levels and immunological parameters will be analyzed by a repeated measurements procedure using a generalized linear model (PROC MIXED of SAS software, SAS version 9.1), which provides a valid statistical estimate of the mean effect. In addition, this methods implies that missing data at specific time points are estimated by the model, based on the observed correlation of the data present and assuming that they are 'missing at random'. The above described analyses will be performed on the intent-to-treat population and on the on-treatment population (all subjects who took more than 75% of the study's doses).

In addition, using a nonlinear mixed effects modeling procedure, an integrated dose-response and exposure-response analysis will be performed on the intent-to-treat population to characterize the relationships between different dose combinations of hydroxyurea and didanosine, their plasma concentrations and their effect on HIV-1 viral load in time, including the associated inter- and intra-individual variabilities. The effects of potential covariates such as subject age, weight, gender, race, creatinine clearance, concomitant diseases, etc. may be included in the model. The model will

be used to simulate different dose combinations of hydroxyurea and didanosine to optimize dose selection for subsequent studies. A detailed description of the analysis will be included in a population PK/PD analysis plan and provided as appendix to the final report.

The incidence of AEs, laboratory abnormalities and the newly developed genotypic resistances will be tabulated per study arm. A detailed statistical analysis plan will be made before database lock and unblinding of the data.

All tests of hypotheses will be two-sided and will be at the 5% level of significance.

12.2. Planned interim analysis

There will be no interim analysis.

12.3. Determination of sample size

As discussed in section 8, no formal sample size calculations are required for this study. However, a total of 12 subjects per study arm is believed to be sufficient for evaluation of the anti-viral activity as measured by viral load. Nonetheless, with 12 evaluable subjects in each study arm, an alpha of 5%, 80% power, and a standard deviation of 0.5 log10 copies HIV-1 RNA, a change from baseline of greater than -0.44 and more than 0.44 log10 copies HIV-1 RNA can be detected per arm. The most promising dose regimens will be taken forward in a properly powered phase IIb/III study. This selection will be based upon an integrated assessment of safety, tolerability, viral load, immunological parameters, and the outcome of the PK/PD analysis.

12.4. Deviations from statistical analysis plan

Before the final analysis, a detailed statistical analysis plan will be written. Any deviations of the statistical plan outlined above will be reported in the final study report.

12.5. Subject population to be analyzed

The analyses will be performed on the intent to treat population, i.e., all randomized subjects, who took at least one dose of study medication. The endpoints defined in paragraph 8.2 will also be analyzed in the on treatment group, i.e., all subjects who

took 75% or more of the study's doses. Furthermore, the PK population is defined as all subjects with PK evaluations and the safety population as all subjects who took at least one dose of the study medication.

13. PHARMACOKINETICS

13.1. Sample collection and handling

For the PK sample collection the patients will be divided in two groups. The first 30 randomized patients will be assigned to Group A and the second 30 randomized patients to Group B. See the PK sampling schedule in Section 4
During Visit 2 (Group A) and 6 (Group B) blood samples for the bioanalysis of didanosine and hydroxyurea will be collected from the subject pre-dose (within 30 minutes before intake of the trial medication) and at 0.5, 1, 2, 3, 4, 6, 9, 12 and 24 hours post-dose (full sampling) or at 1, 2 and 4 hours post dose (sparse sampling).. Samples to determine intracellular PK of didanosine and hydroxyurea will also be taken at 24 hours predose (Visit 2 and 6). At Visit 7, a blood sample of 3 mL will be drawn 24 hours after the last dose of the study drug.

Plasma will be divided into two tubes. One tube will be used for bioanalysis and the second will serve as a back-up sample. Back-up samples will be kept on site until completion of the bioanalytical study reports.

All blood samples will be processed, handled and identified according to the laboratory manual, which will be provided before the start of the study. Exact times of blood sampling will be recorded on the pharmacokinetic blood sampling sections of the CRF. Samples will be stored at ≤ -18°C until assayed. Plasma samples will be transferred on dry ice to ABL B.V., Assen, The Netherlands, accompanied by a sample accountability form.

13.2. Bioanalysis

Bioanalysis will be performed by ABL B.V., Assen, The Netherlands. Plasma concentrations of hydroxyurea and didanosine will be determined using a validated liquid chromatography mass spectrometry/mass spectrometry (LC-MS/MS) method. A description of the bioanalysis will be included in the final report.

13.3. Noncompartmental Pharmacokinetic Analyses

Pharmacokinetic analysis will be performed by Kinesis Pharma B.V., Breda, The Netherlands.

Based on the individual plasma concentration-time data, using the actual sampling times, the following pharmacokinetic parameters of didanosine and hydroxyurea will be derived from the bioanalytical results for the full PK sampling schemes:

Day 1:

t_{max}, C_{max}, $AUC_{24h,}$ λ_z, $t_{1/2term}$

Day 29 (Week 4):

t_{max}, C_{0h}, C_{min}, C_{max}, AUC_{24h}, λ_z, $t_{1/2term}$

For the pharmacokinetic parameters, definitions and methods of calculations are:

t_{max}	Time to reach the maximal plasma concentration.
C_{max}	Maximal plasma concentration.
C_{0h}	Predose plasma concentration
C_{min}	Minimal plasma concentration between 0 hour and 24 h (=dosing interval)
AUC_{24h}	Area under the plasma concentration-time curve from time of administration up to 24 hours post dosing, calculated by linear up-logarithmic down trapezoidal summation.
λ_z	Elimination rate constant, determined by linear regression of the terminal points of the ln-linear plasma concentration-time curve.
$t_{1/2term}$	Terminal elimination half-life, defined as $0.693/\lambda_z$.

Pharmacokinetic and statistical analyses will be done using WinNonlin Professional™ (Pharsight Corporation, Mountain View, California, U.S.A.) and/or Microsoft Excel® (Microsoft Redmond, Washington, U.S.A.) and/or SAS (SAS Institute Inc., Cary, NC, U.S.A). A noncompartmental model with extra vascular input will be used for the pharmacokinetic analysis.

For each treatment descriptive statistics will be calculated for the plasma concentrations of didanosine and hydroxyurea at each time point, the intracellular concentrations of didanosine and hydroxyurea, and for the derived pharmacokinetic parameters. Statistics include, Number of subjects (N), mean, standard deviation

(SD), percentage coefficient of variation (%CV), geometric mean, median, minimum, and maximum.

For each subject, plasma concentration-time data of didanosine and hydroxyurea will be graphically presented. Similarly, graphs of the mean plasma concentration-time profiles and graphs with combined individual plasma concentration-time profiles will be produced per treatment. Pharmacokinetic parameters and the intracellular concentrations of didanosine and hydroxyurea will be subjected to an exploratory graphical analysis to investigate dose-proportionality of didanosine and hydroxyurea in the investigated dosing range and to compare the pharmacokinetics between Day 1 and Day 15.

14. DATA MANAGEMENT

14.1. Direct access to source data

The investigator will permit trial-related monitoring, audits IRB/IEC review and regulatory inspection, providing direct access to source data/documents, for which the subject has given written consent.

14.2. Data collection

Source data must be available to document the existence of the study subjects and should substantiate integrity of study data collected. Source data must include the original documents relating to the study, the medical treatment and medical history of the subject.

The following information should be included in the source medical records:

- Demographic data (date of birth, sex, ethnic group);
- Details related to the inclusion criteria;
- Date of signing informed consent form;
- Medical history and physical examination details;
- Adverse events and concomitant treatment;
- Results of relevant examinations;
- Laboratory print-outs;
- Visit dates and dispensing of study medication.

Data collected on each study subject will be recorded in a case report form (CRF). The investigator is responsible for ensuring that all sections in the CRF are completed correctly and that entries can be verified against source data. If data have been entered incorrectly, a single line should be drawn through them and the correct data should be entered together with the initials of the person making the correction and date. CRFs should be completed using a black or blue ball-point pen.

All data are recorded (electronically, written) in the patient's medical record (or other source document) before transcription to the CRF, direct CRF entries are not allowed. The monitor will perform a 100% source data verification for all subjects.

14.3. Monitoring

The study will be monitored by IATEC. The monitor will visit the study site at regular intervals. These visits will be announced and confirmed in writing. The investigator will provide all source data and CRFs to the monitor for source data verification of the CRF data. The monitor will visit the laboratory and/or the pharmacy on a regular basis to ensure that all study procedures are followed.
The investigator will reserve adequate time to discuss the study progress with the monitor, as well as the results from the source data verification. The investigator agrees that the monitor will have access to all required documents and facilities.

14.4. Data management

Data management of the study data will be handled by IATEC.
All completed CRFs are collected from the study site at regular intervals. The CRF data will be entered in eDM, a web-based data management system, which is fully validated and compliant to 21 CFR, part 11. Medical history and adverse events will be coded by using MedDRA. Medication history and (concomitant) medication will be coded by using the WHO Drug dictionary.
Data validation checks are performed at regular intervals and will result in data queries. The monitor will discuss the queries with the investigator. The resolved queries will be signed for confirmation by the investigator. The completed queries are returned to the Data Management Department and the database is updated subsequently. A number of data changes will be made directly by IATEC. These

changes are documented in the study specific Data Management Plan, which will be signed by the external party and IATEC.

The study database is considered clean and can be locked only after all data have been received, all data problems are solved, all data checks and quality control have been performed, and a data review meeting has been held.

14.5. Quality Assurance

The QA manager or a qualified delegate may periodically perfom audits of the study by reviewing the data obtained as well as the procedural aspects. This may include on-site inspections and source data checks. Direct access to source documents is required for the purpose of these periodical inspections.

15. ADMINISTRATIVE MATTERS

15.1. Financing and Insurance

VS411-C201 is financed by Virostatics, S.R.L.

A Clinical Trial Agreement between all parties involved in the study will be in place prior the start of the study.

The sponsor should arrange adequate subject insurance for the period of the study.

15.2. Investigator Indemnity

The sponsor agrees to, and does hereby, indemnify, defend and hold the investigator harmless from and against all claims, demands, actions and proceedings, which may be brought or asserted against the investigator to recover damages and losses for or attributable to bodily injury, sickness, disease, or death, arising from or alleged to arise from or be reasonable attributable to the above study.

Further details of the indemnity are specified in the agreement between the investigator and the sponsor.

15.3. Regulatory and Ethics Committee approval

Before the initiation of a study regulatory approval from the national authorities and local ethical committees or Institutional Review Boards will be obtained for conduct of the study.

The study will only start after written approval from an Independent Ethics Committee (IEC), which operates according to ICH GCP guidelines. The written IEC approval and the names and qualifications of members of the IEC must be made available to the sponsor before the study can start. The investigator is, together with the sponsor, responsible for submission to and communication with the IEC.

The investigator should conduct the study in accordance with this protocol, the declaration of Helsinki and the ICH GCP guidelines (CPMP/ICH/135/95). The investigator and the sponsor will sign the protocol and study agreement to confirm this. The investigator will not implement any amendment of the protocol without written agreement by the sponsor, and IEC approval, except when necessary to eliminate immediate hazard to study subjects.

15.4. Subject information and informed consent

The investigator is responsible for ensuring that the subject fully understands the nature and purpose of the study. Information should be given in both oral and written form. No subject should be obliged to participate in the study. Subjects, their relatives, guardians or, if applicable, legal representatives, must be given ample opportunity to enquire about details of the study. The information must make clear that refusal to participate or withdrawal from the study at any stage is without any prejudice to the subject's subsequent care. Subjects must be allowed sufficient time to decide whether or not they wish to participate.

The subject must be made aware of (and give consent to) the fact that monitors, auditors, the IEC and regulatory authorities will be granted direct access to the subjects medical records without violating subject confidentiality, and to the extent permitted by applicable regulations. The subject should be informed that by signing the informed consent form, the subject authorizes such access.

The subject needs to give written informed consent before start of any study related procedure. The signed informed consents will be retained by the investigator and made available (for review only) to the study monitor, auditor and inspector, upon request.

The subject must be informed that biological samples taken within the framework of the study will be stored for a maximum of 15 years. These samples will be anonymised and used only for the scientific objectives described in this protocol, excluding any commercial exploitation. Samples may be used by the sponsor for further disease related exploratory work e.g. on pharmacokinetics, biomarkers, biochemistry, viral DNA analysis, etc. No human DNA analysis will be performed unless it provides information to investigate underlying factors of the metabolism of VS411 or the disease under investigation. Use of these samples for any other scientific purpose will be the object of a new specific information and informed consent.

15.5. (Subject) Confidentiality

All information concerning the product and the sponsor's operations (such as patent applications, formulae, manufacturing processes, basic scientific data, or formulation information supplied to the investigator by the sponsor and not previously published) is considered confidential by the sponsor and should not be disclosed by the investigator to any third party without the sponsor's prior written approval. The investigator agrees to use this information only in accomplishing the trial and will not use it for other purposes. In addition, individual subject medical information, obtained as result of this study is considered confidential and disclosure to third parties is prohibited. Such medical information may be given to the subject's physician or to other appropriate medical personnel responsible for the subject's well being.

Data generated as a result of this study are to be available for inspection on request by the participating physicians, the sponsor's monitor, the IEC and the regulatory health authorities, including external site audits and inspections.

15.6. Amendments

An amendment is a written descripton of a change(s) to or formal clarification of a protocol. All amendments will be provided to the IEC that gave a favorable opinion.

A 'substantial amendment' is defined as an amendment to the terms of the IEC application, or to the protocol or any other supporting documentation, that is likely to affect to a significant degree:

- The safety or physical or mental integrity of the subjects of the trial;
- The scientific value of the trial;
- The conduct or management of the trial; or
- The quality or safety of any intervention used in the trial.

All substantial amendments will be provided to the IEC that gave a favorable opinion.

Non-substantial amendments will be sent to the accredited IEC for notification. Examples of non-substantial amendments are typing errors and administrative changes such as changes in names, telephone numbers and other contact details of involved persons mentioned in the submitted study documentation.

15.7. Annual progress report

The investigator will submit a summary of the progress of the trial to the accredited IEC once a year. Information will be provided on the date of inclusion of the first subject, numbers of subjects included and numbers of subjects that have completed the trial, SAEs/SUSARs, amendments and any other relevant issues concerning the execution of the trial

15.8. End of study report

The investigator will notify (by themselves or by the sponsor or the sponsors' designee) the accredited IEC [and the competent authority] of the end of the study within a period of 12 weeks [90 days]. The end of the study is defined as the last subject's last visit.

In case the study is ended prematurely, the investigator (or by themselves or by the sponsor or the sponsors' designee) will notify the accredited IEC [and the competent authority within 15 days], including the reasons for the premature termination.

Within one year after the end of the study, the investigator will submit a final study report with the results of the study, including any publications/abstracts of the study, to the accredited IEC [and the Competent Authority].

15.9. Publication policy

All information produced and/or transferred between the sponsor and the investigator and subinvestigators in the course of the clinical trial is subject to the hereabove reported confidentiality clause (see Section 15.5).
The results of this trial will be published if applicable. Any presentation, abstract, or manuscript will be made available for review by the site investigator prior to submission. Any presentation of the study data by the investigator or his/her designee, in writing or as an oral presentation, must be approved by Virostatics as owner of the data."

15.10. Study documentation and record keeping

The study documentation includes the following documents:

- Study protocol (and amendments, if applicable);
- IB/ Investigational Medicinal Product Dossier;
- Subject information documents (including Informed Consent);
- CRFs and SAE report forms;
- Investigator's Site File;
- Study medication documentation;
- Study contract.

In order to start the study, the investigator is required to have the following documentation available:

- Signed confidentiality agreement;
- Signed investigator statement for this protocol;
- Signed study contract;
- IEC approval letter, stating the sponsor's name, study number and investigational drug, as well as all documents reviewed and should include a list of members present at the meeting;

- Recent curriculum vitaes of investigators and sub investigators (signed and dated);
- Signature sheet, documenting delegation of tasks and names and initials of the study team;
- Laboratory normal ranges and quality certificates and/or accreditations.

During the study, the sponsor should have collected:

- All study medication related completed documentation;
- All left-over study medication.

During the study IATEC should have collected all completed CRFs.

The investigator will archive all study data (source data, CRF copies and Investigator Site File) and relevant correspondence. These documents are to be kept on file for at least fifteen years after completion of the study. The sponsor will archive and retain all documents pertaining to the study for the lifetime of the product.

15.11. Sample handling

Blood samples will be collected at the study site. The study site will be responsible for cytopreservation, separation and storage of all samples collected at the study site as prescribed by the protocol.

The remainder of the samples which may need to be transported to other laboratories will be stored at the study site for three years, which might be extended if necessary.The study site will provide yearly reports of the use and fate of the blood samples to the Steering Committee, including the availability of any unused material.

All laboratories involved shall use the human materials sampled in the study only in accordance with the agreed Protocol, the international Good Clinical Practice principles as laid down by ICH GCP guidelines, the EU Clinical Trial Directive and other relevant legislation or regulatory requirements as may be required in the Territory.

Upon first request by the VS411-C201 Coordinating Committee, the study site shall supply guidance on handling and storage of the Materials to the VS411-C201 Coordinating Committee and shall provide the VS411-C201 Coordinating Committee with all other related relevant information.

16. REFERENCES

For further information on product characteristics, preclinical or clinical data of (the combination of) ddI and HU, please consult the Investigator's Brochure.

APPENDIX I: CDC classification system for HIV infection (revision 1993)

Reference: 1993 revised classification system for HIV infection and expanded surveillance case definition for AIDS among adolescents and adults.
MMWR 1992; 41 (No. RR-17): 1-19.

The clinical categories of HIV infection are defined as follows:

Category A

Category A consists of one or more of the conditions listed below in an adolescent or adult (≥ 13 years) with documented HIV infection. Conditions listed in Categories B and C must not have occurred.

- Asymptomatic HIV infection
- Persistent generalized lymphadenopathy
- Acute (primary) HIV infection with accompanying illness or history of acute HIV infection

***Category B* (Symptomatic non-AIDS conditions)**

Category B consists of symptomatic conditions in an HIV-infected adolescent or adult that are not included among conditions listed in clinical Category C and that meet at least one of the following criteria: a) the conditions are attributed to HIV infection or are indicative of a defect in cell-mediated immunity; or b) the conditions are considered by physicians to have a clinical course or to require management that is complicated by HIV infection. **Examples** of conditions in clinical category B include, **but are not limited to:**

- Bacillary angiomatosis
- Candidiasis, oropharyngeal (thrush)
- Candidiasis, vulvovaginal; persistent, frequent, or poorly responsive to therapy
- Cervical dysplasia (moderate or severe)/cervical carcinoma in situ
- Constitutional symptoms, such as fever (38.5 C) or diarrhoea lasting > 1 month
- Hairy leukoplakia, oral
- Herpes zoster (shingles), involving at least 2 distinct episodes or more than one dermatome
- Idiopathic thrombocytopenic purpura
- Listeriosis

- Pelvic inflammatory disease, particularly if complicated by tubo-ovarian abscess
- Peripheral neuropathy

For classification purposes, Category B conditions take precedence over those in Category A. For example, someone previously treated for oral or persistent vaginal candidiasis (and who has not developed a Category C disease) but who is now asymptomatic should be classified in clinical Category B.

Category C **(AIDS indicator conditions as defined by diagnostic or presumptive** measures)

Category C includes the clinical conditions listed in the AIDS surveillance case definition. For classification purposes, once a Category C condition has occurred, the person will remain in Category C.

Conditions in Category C include:

- Candidiasis of bronchi, trachea, or lungs
- Candidiasis, oesophageal
- Cervical cancer, invasive
- Coccidiomycosis, disseminated or extrapulmonary
- Cryptococcosis, extrapulmonary
- Cryptosporidiosis, chronic intestinal (> 1 month's duration)
- Cytomegalovirus disease (other than liver, spleen or nodes)
- Cytomegalovirus retinitis (with loss of vision)
- Encephalopathy, HIV-related
- Herpes simplex: chronic ulcer(s) (> I month's duration); or bronchitis, pneumonitis, or oesophagitis
- Histoplasmosis, disseminated or extrapulmonary
- Isosporiasis, chronic intestinal (> 1 month's duration)
- Kaposi's sarcoma
- Lymphoma, Burkitt's (or equivalent term)
- Lymphoma, immunoblastic (or equivalent term)
- Lymphoma, primary, or brain
- *Mycobacterium avium* complex or *M. kansaii*, disseminated or extrapulmonary
- *Mycobacterium tuberculosis*, any site (pulmonary or extrapulmonary)
- *Mycobacterium*, other species or unidentified species, disseminated or extrapulmonary

- *Pneumocystis carinii* pneumonia
- Pneumonia, current
- Progressive multifocal leukoencephalopathy
- *Salmonella* septicaemia, recurrent
- Toxoplasmosis of brain
- Wasting syndrome due to HIV

The following list of other non-CDC HIV-associated conditions was compiled, as the CDC notes that Category B is not limited to events listed in the CDC 1993 definition.

- Aspergillosis
- Leishmaniasis
- Microsporidiosis
- Molluscum contagiosum
- Nocardiasis
- Thrombotic microangiopathy (hemolytic uremic syndrome [HUS]/ thrombotic thrombocytopenia purpura [TTP]

Appendix II: table for grading adverse experiences

DIVISION OF AIDS TABLE FOR GRADING THE SEVERITY OF ADULT AND PEDIATRIC ADVERSE EVENTS. PUBLISH DATE: DECEMBER, 2004

Quick Reference

The Division of AIDS Table for Grading the Severity of Adult and Pediatric Adverse Events ("DAIDS grading table") is a descriptive terminology to be utilized for adverse event reporting in this trial. A grading (severity) scale is provided for each adverse event term.

General Instructions

Estimating Severity Grade

If the need arises to grade a clinical adverse event that is not identified in the DAIDS grading table, use the category "Estimating Severity Grade" located at the top of the table on the following page.

Grading Adult and Pediatric Adverse Events

The DAIDS grading table includes parameters for grading both adult and pediatric adverse events. When a single set of parameters is not appropriate for grading specific types of adverse events for both adult and pediatric populations, separate sets of parameters for adult and/or pediatric populations (with specified respective age ranges) are provided. If there is no distinction in the table between adult and pediatric values for a type of adverse event, then the single set of parameters listed is to be used for grading the severity of both adult and pediatric events of that type.

Determining Severity Grade

If the severity of an adverse event could fall under either one of two grades (e.g., the severity of an adverse event could be either grade 2 or grade 3), select the higher of the two grades for the adverse event.

Note: The laboratory normal ranges should be taken into consideration to assign gradings to a laboratory value.

Definitions

Basic self-care functions	Adult: activities such as bathing, dressing, toileting, transfer/movement, continence, and feeding.<br>Young children: activities that are age and culturally appropriate (e.g., feeding self with culturally appropriate eating implement).
Usual social &	Adult: adaptive tasks and desirable activities, such as going to work,

functional activities	shopping, cooking, use of transportation, pursuing a hobby, etc.<br>Young Children: activities that are age and culturally appropriate (e.g., social interactions, play activities, learning tasks, etc.).
Medical intervention	Use of pharmacologic or biologic agent(s) for treatment of an adverse event.
Operative intervention	Surgical OR other invasive mechanical procedures.

PARAMETER	GRADE 1<br>MILD	GRADE 2<br>MODERATE	GRADE 3<br>SEVERE	GRADE 4<br>POTENTIALLY LIFE-THREATENING
**ESTIMATING SEVERITY GRADE**				
Clinical adverse event NOT identified elsewhere in this DAIDS grading table	Symptoms causing no or minimal interference with usual social & functional activities	Symptoms causing greater than minimal interference with usual social & functional activities	Symptoms causing inability to perform usual social & functional activities	Symptoms causing inability to perform basic self-care functions OR Medical or operative intervention indicated to prevent permanent impairment, persistent disability, or death
**SYSTEMIC**				
Acute systemic allergic reaction	Localized urticaria (wheals) with no medical intervention indicated	Localized urticaria with medical intervention indicated OR Mild angioedema with no medical intervention indicated	Generalized urticaria OR Angioedema with medical intervention indicated OR Symptomatic mild bronchospasm	Acute anaphylaxis OR Life-threatening bronchospasm OR laryngeal edema
Chills	Symptoms causing no or minimal interference with usual social & functional activities	Symptoms causing greater than minimal interference with usual social & functional activities	Symptoms causing inability to perform usual social & functional activities	NA
Fatigue<br>Malaise	Symptoms causing no or minimal interference with usual social & functional activities	Symptoms causing greater than minimal interference with usual social & functional activities	Symptoms causing inability to perform usual social & functional activities	Incapacitating fatigue/ malaise symptoms causing inability to perform basic self-care functions
Fever (nonaxillary)	37.7°C – 38.6°C	38.7°C – 39.3°C	39.4°C – 40.5°C	> 40.5°C
Pain (indicate body site)<br>DO NOT use for pain due to injection (See Injection site reactions: Injection site pain)<br>See also Headache, Arthralgia, and Myalgia	Pain causing no or minimal interference with usual social & functional activities	Pain causing greater than minimal interference with usual social & functional activities	Pain causing inability to perform usual social & functional activities	Disabling pain causing inability to perform basic self-care functions OR Hospitalization (other than emergency room visit) indicated

CLINICAL				
**PARAMETER**	**GRADE 1**<br>**MILD**	**GRADE 2**<br>**MODERATE**	**GRADE 3**<br>**SEVERE**	**GRADE 4**<br>**POTENTIALLY LIFE-THREATENING**
Unintentional weight loss	NA	5% – 9% loss in body weight from baseline	10% – 19% loss in body weight from baseline	≥ 20% loss in body weight from baseline OR Aggressive intervention indicated [e.g., tube feeding or total parenteral nutrition (TPN)]
**INFECTION**				
Infection (any other than HIV infection)	Localized, no systemic antimicrobial treatment indicated AND Symptoms causing no or minimal interference with usual social & functional activities	Systemic antimicrobial treatment indicated OR Symptoms causing greater than minimal interference with usual social & functional activities	Systemic antimicrobial treatment indicated AND Symptoms causing inability to perform usual social & functional activities OR Operative intervention (other than simple incision and drainage) indicated	Life-threatening consequences (e.g., septic shock)
**INJECTION SITE REACTIONS**				
Injection site pain (pain without touching)<br>Or<br>Tenderness (pain when area is touched)	Pain/tenderness causing no or minimal limitation of use of limb	Pain/tenderness limiting use of limb OR Pain/tenderness causing greater than minimal interference with usual social & functional activities	Pain/tenderness causing inability to perform usual social & functional activities	Pain/tenderness causing inability to perform basic self-care function OR Hospitalization (other than emergency room visit) indicated for management of pain/tenderness
Injection site reaction (localized)				
**Adult > 15 years**	Erythema OR Induration<br>of 5 x 5 cm – 9 x 9 cm (or 25 cm² – 81cm²)	Erythema OR Induration OR Edema<br>> 9 cm any diameter<br>(or > 81 cm²)	Ulceration OR Secondary infection OR Phlebitis OR Sterile abscess OR Drainage	Necrosis (involving dermis and deeper tissue)
**Pediatric ≤ 15 years**	Erythema OR Induration OR Edema present but ≤ 2.5 cm diameter	Erythema OR Induration OR Edema > 2.5 cm diameter but < 50% surface area of the extremity segment (e.g., upper arm/thigh)	Erythema OR Induration OR Edema involving ≥ 50% surface area of the extremity segment (e.g., upper arm/thigh) OR Ulceration OR Secondary infection OR Phlebitis OR Sterile abscess OR Drainage	Necrosis (involving dermis and deeper tissue)

CLINICAL				
PARAMETER	GRADE 1<br>MILD	GRADE 2<br>MODERATE	GRADE 3<br>SEVERE	GRADE 4<br>POTENTIALLY LIFE-THREATENING
Pruritis associated with injection<br>See also Skin: Pruritis (itching - no skin lesions)	Itching localized to injection site AND Relieved spontaneously or with < 48 hours treatment	Itching beyond the injection site but not generalized OR Itching localized to injection site requiring ≥ 48 hours treatment	Generalized itching causing inability to perform usual social & functional activities	NA
**SKIN – DERMATOLOGICAL**				
Alopecia	Thinning detectable by study participant (or by caregiver for young children and disabled adults)	Thinning or patchy hair loss detectable by health care provider	Complete hair loss	NA
Cutaneous reaction/rash	Localized macular rash	Diffuse macular, maculopapular, or morbilliform rash OR Target lesions	Diffuse macular, maculopapular, or morbilliform rash with vesicles or limited number of bullae OR Cutaneous reaction /rash with superficial ulcerations of mucous membrane limited to 1 site[a] OR Cuaneous reaction/rash with at least 1 of the following[a]: elevation of AST and/or ALT > 2 x baseline but at least > 5 x ULN[a]; fever (> 38°C or 100°F)[a]; eosinophils > 1000/mm[3a]; serum sickness-like reaction[a]	Extensive or generalized bullous lesions OR Stevens-Johnson syndrome (SJS) OR Ulceration of mucous membrane involving 2 or more distinct mucosal sites OR Toxic epidermal necrolysis (TEN)
Hyperpigmentation	Slight or localized	Marked or generalized	NA	NA
Hypopigmentation	Slight or localized	Marked or generalized	NA	NA
Pruritis (itching – no skin lesions)<br>(See also Injection site reactions: Pruritis associated with injection)	Itching causing no or minimal interference with usual social & functional activities	Itching causing greater than minimal interference with usual social & functional activities	Itching causing inability to perform usual social & functional activities	NA
**CARDIOVASCULAR**				
Cardiac arrhythmia (general) (By ECG or physical exam)	Asymptomatic AND No intervention indicated	Asymptomatic AND Nonurgent medical intervention indicated	Symptomatic, non-life threatening AND Nonurgent medical intervention indicated	Life-threatening arrhythmia OR Urgent intervention indicated

[a] Revised.

CLINICAL				
PARAMETER	GRADE 1<br>MILD	GRADE 2<br>MODERATE	GRADE 3<br>SEVERE	GRADE 4<br>POTENTIALLY LIFE-THREATENING
Cardiac ischemia/ infarction	NA	NA	Symptomatic ischemia (stable angina) OR Testing consistent with ischemia	Unstable angina OR Acute myocardial Infarction
Hemorrhage (significant acute blood loss)	NA	Symptomatic AND No transfusion indicated	Symptomatic AND Transfusion of ≤ 2 units packed RBCs (for children ≤ 10 cc/kg) indicated	Life-threatening hypotension OR Transfusion of > 2 units packed RBCs (for children > 10 cc/kg) indicated
Hypertension[a]				
**Adult > 17 years** (with repeat testing at same visit)	> 140 to ≤ 160 mmHg systolic<br>OR<br>> 90 to ≤ 100 mmHg diastolic	> 160 to ≤ 180 mmHg systolic<br>OR<br>> 100 to ≤ 110 mmHg diastolic	> 180 mmHg systolic<br>OR<br>> 110 mmHg diastolic	Life-threatening consequences (e.g., malignant hypertension) OR Hospitalization indicated (other than emergency room visit)
**Pediatric ≤ 17 years** (with repeat testing at same visit)	NA	91st – 94th percentile adjusted for age, height, and gender (systolic and/or diastolic)	≥ 95th percentile adjusted for age, height, and gender (systolic and/or diastolic)	Life-threatening consequences (e.g., malignant hypertension) OR Hospitalization indicated (other than emergency room visit)
Hypotension	NA	Symptomatic, corrected with oral fluid replacement	Symptomatic, i.v. fluids indicated	Shock requiring use of vasopressors or mechanical assistance to maintain blood pressure
Pericardial effusion	Asymptomatic, small effusion requiring no intervention	Asymptomatic, moderate or larger effusion requiring no intervention	Effusion with non-life threatening physiologic consequences OR Effusion with nonurgent intervention indicated	Life-threatening consequences (e.g., tamponade) OR Urgent intervention indicated
Prolonged PR interval				
**Adult > 16 years**	PR interval 0.21 – 0.25 s	PR interval > 0.25 s	Type II 2nd degree AV block OR Ventricular pause > 3.0 s	Complete AV block
**Pediatric ≤ 16 years**	1st degree AV block (PR > normal for age and rate)	Type I 2nd degree AV block	Type II 2nd degree AV block	Complete AV block

[a] Revised.

PARAMETER	GRADE 1<br>MILD	GRADE 2<br>MODERATE	GRADE 3<br>SEVERE	GRADE 4<br>POTENTIALLY LIFE-THREATENING
**CLINICAL**				
Prolonged QTc				
**Adult > 16 years**	Asymptomatic, QTc interval 0.45 – 0.47 s OR Increase in interval < 0.03 s above baseline	Asymptomatic, QTc interval 0.48 – 0.49 s OR Increase in interval 0.03 – 0.05 s above baseline	Asymptomatic, QTc interval ≥ 0.50 s OR Increase in interval ≥ 0.06 s above baseline	Life-threatening consequences, e.g., Torsade de pointes or other associated serious ventricular dysrhythmia
**Pediatric ≤ 16 years**	Asymptomatic, QTc interval 0.450 – 0.464 s	Asymptomatic, QTc interval 0.465 – 0.479 s	Asymptomatic, QTc interval ≥ 0.480 s	Life-threatening consequences, e.g., Torsade de pointes or other associated serious ventricular dysrhythmia
Thrombosis/embolism	NA	Deep vein thrombosis AND No intervention indicated (e.g., anticoagulation, lysis filter, invasive procedure)	Deep vein thrombosis AND Intervention indicated (e.g., anticoagulation, lysis filter, invasive procedure)	Embolic event (e.g., pulmonary embolism, life-threatening thrombus)
Vasovagal episode (associated with a procedure of any kind)	Present without loss of consciousness	Present with transient loss of consciousness	NA	NA
Ventricular dysfunction (congestive heart failure)	NA	Asymptomatic diagnostic finding AND intervention indicated	New onset with symptoms OR Worsening symptomatic congestive heart failure	Life-threatening congestive heart failure
**GASTROINTESTINAL**				
Anorexia	Loss of appetite without decreased oral intake	Loss of appetite associated with decreased oral intake without significant weight loss	Loss of appetite associated with significant weight loss	Life-threatening consequences OR Aggressive intervention indicated (e.g., tube feeding or total parenteral nutrition [TPN])
Ascites	Asymptomatic	Symptomatic AND Intervention indicated (e.g., diuretics or therapeutic paracentesis)	Symptomatic despite intervention	Life-threatening consequences

CLINICAL				
**PARAMETER**	**GRADE 1**<br>**MILD**	**GRADE 2**<br>**MODERATE**	**GRADE 3**<br>**SEVERE**	**GRADE 4**<br>**POTENTIALLY LIFE-THREATENING**
Cholecystitis	NA	Symptomatic AND Medical intervention indicated	Radiologic, endoscopic, or operative intervention indicated	Life-threatening consequences (e.g., sepsis or perforation)
Constipation	NA	Persistent constipation requiring regular use of dietary modifications, laxatives, or enemas	Obstipation with manual evacuation indicated	Life-threatening consequences (e.g., obstruction)
Diarrhea				
**Adult and Pediatric ≥ 1 year**	Transient or intermittent episodes of unformed stools OR Increase of ≤ 3 stools over baseline per 24-hour period	Persistent episodes of unformed to watery stools OR Increase of 4 – 6 stools over baseline per 24-hour period	Bloody diarrhea OR Increase of ≥ 7 stools per 24-hour period OR i.v. fluid replacement indicated	Life-threatening consequences (e.g., hypotensive shock)
**Pediatric < 1 year**	Liquid stools (more unformed than usual) but usual number of stools	Liquid stools with increased number of stools OR Mild dehydration	Liquid stools with moderate dehydration	Liquid stools resulting in severe dehydration with aggressive rehydration indicated OR Hypotensive shock
Dysphagia-Odynophagia	Symptomatic but able to eat usual diet	Symptoms causing altered dietary intake without medical intervention indicated	Symptoms causing severely altered dietary intake with medical intervention indicated	Life-threatening reduction in oral intake
Mucositis/stomatitis (clinical exam)<br>Indicate site (e.g., larynx, oral)<br>See Genitourinary for Vulvovaginitis<br>See also Dysphagia-Odynophagia and Proctitis	Erythema of the Mucosa	Patchy pseudomembranes or ulcerations	Confluent pseudomembranes or ulcerations OR Mucosal bleeding with minor trauma	Tissue necrosis OR Diffuse spontaneous mucosal bleeding OR Life-threatening consequences (e.g., aspiration, choking)
Nausea	Transient (< 24 hours) or intermittent nausea with no or minimal interference with oral intake	Persistent nausea resulting in decreased oral intake for 24 – 48 hours	Persistent nausea resulting in minimal oral intake for > 48 hours OR Aggressive rehydration indicated (e.g., i.v. fluids)	Life-threatening consequences (e.g., hypotensive shock)

PARAMETER	GRADE 1<br>MILD	GRADE 2<br>MODERATE	GRADE 3<br>SEVERE	GRADE 4<br>POTENTIALLY LIFE-THREATENING
**CLINICAL**				
Pancreatitis	NA	Symptomatic AND Hospitalization not indicated (other than emergency room visit)	Symptomatic AND Hospitalization indicated (other than emergency room visit)	Life-threatening consequences (e.g., circulatory failure, hemorrhage, sepsis)
Proctitis (<u>functional-symptomatic</u>)<br>Also see Mucositis/stomatitis for clinical exam	Rectal discomfort AND No intervention Indicated	Symptoms causing greater than minimal interference with usual social & functional activities OR Medical intervention indicated	Symptoms causing inability to perform usual social & functional activities OR Operative intervention indicated	Life-threatening consequences (e.g., perforation)
Vomiting	Transient or intermittent vomiting with no or minimal interference with oral intake	Frequent episodes of vomiting with no or mild dehydration	Persistent vomiting resulting in orthostatic hypotension OR Aggressive rehydration indicated (e.g., i.v. fluids)	Life-threatening consequences (e.g., hypotensive shock)
**NEUROLOGIC**				
Alteration in personality-behavior or in mood (e.g., agitation, anxiety, depression, mania, psychosis)	Alteration causing no or minimal interference with usual social & functional activities	Alteration causing greater than minimal interference with usual social & functional activities	Alteration causing inability to perform usual social & functional activities	Behavior potentially harmful to self or others (e.g., suicidal and homicidal ideation or attempt, acute psychosis) OR Causing inability to perform basic self-care functions
Altered Mental Status<br>For Dementia, see Cognitive and behavioral/attentional disturbance (including dementia and attention deficit disorder)	Changes causing no or minimal interference with usual social & functional activities	Mild lethargy or somnolence causing greater than minimal interference with usual social & functional activities	Confusion, memory impairment, lethargy, or somnolence causing inability to perform usual social & functional activities	Delirium OR obtundation, OR coma
Ataxia	Asymptomatic ataxia detectable on exam OR Minimal ataxia causing no or minimal interference with usual social & functional activities	Symptomatic ataxia causing greater than minimal interference with usual social & functional activities	Symptomatic ataxia causing inability to perform usual social & functional activities	Disabling ataxia causing inability to perform basic self-care functions

CLINICAL				
**PARAMETER**	**GRADE 1**<br>**MILD**	**GRADE 2**<br>**MODERATE**	**GRADE 3**<br>**SEVERE**	**GRADE 4**<br>**POTENTIALLY LIFE-THREATENING**
Cognitive and behavioral/attentional disturbance (including dementia and attention deficit disorder)	Disability causing no or minimal interference with usual social & functional activities OR Specialized resources not indicated	Disability causing greater than minimal interference with usual social & functional activities OR Specialized resources on part-time basis indicated	Disability causing inability to perform usual social & functional activities OR Specialized resources on a full-time basis indicated	Disability causing inability to perform basic self-care functions OR Institutionalization indicated
CNS ischemia (acute)	NA	NA	Transient ischemic Attack	Cerebral vascular accident (CVA, stroke) with neurological deficit
Developmental delay<br>**Pediatric ≤ 16 years**	Mild developmental delay, either motor or cognitive, as determined by comparison with a developmental screening tool appropriate for the setting	Moderate developmental delay, either motor or cognitive, as determined by comparison with a developmental screening tool appropriate for the setting	Severe developmental delay, either motor or cognitive, as determined by comparison with a developmental screening tool appropriate for the setting	Developmental regression, either motor or cognitive, as determined by comparison with a developmental screening tool appropriate for the setting
Headache	Symptoms causing no or minimal interference with usual social & functional activities	Symptoms causing greater than minimal interference with usual social & functional activities	Symptoms causing inability to perform usual social & functional activities	Symptoms causing inability to perform basic self-care functions OR Hospitalization indicated (other than emergency room visit) OR Headache with significant impairment of alertness or other neurologic function
Insomnia	NA	Difficulty sleeping causing greater than minimal interference with usual social & functional activities	Difficulty sleeping causing inability to perform usual social & functional activities	Disabling insomnia causing inability to perform basic self-care functions
Neuromuscular weakness (including myopathy & neuropathy)	Asymptomatic with decreased strength on exam OR Minimal muscle weakness causing no or minimal interference with usual social & functional activities	Muscle weakness causing greater than minimal interference with usual social & functional activities	Muscle weakness causing inability to perform usual social & functional activities	Disabling muscle weakness causing inability to perform basic self-care functions OR Respiratory muscle weakness impairing ventilation

CLINICAL				
**PARAMETER**	**GRADE 1**<br>**MILD**	**GRADE 2**<br>**MODERATE**	**GRADE 3**<br>**SEVERE**	**GRADE 4**<br>**POTENTIALLY LIFE-THREATENING**
Neurosensory alteration (including paresthesia and painful neuropathy)	Asymptomatic with sensory alteration on exam or minimal paresthesia causing no or minimal interference with usual social & functional activities	Sensory alteration or paresthesia causing greater than minimal interference with usual social & functional activities	Sensory alteration or paresthesia causing inability to perform usual social & functional activities	Disabling sensory alteration or paresthesia causing inability to perform basic self-care functions
Seizure: (new onset)<br>**Adult ≥ 18 years**<br>See also Seizure: (known pre-existing seizure disorder)	NA	1 seizure	2 – 4 seizures	Seizures of any kind which are prolonged, repetitive (e.g., status epilepticus), or difficult to control (e.g., refractory epilepsy)
Seizure: (known pre-existing seizure disorder)<br>**Adult ≥ 18 years**<br>For worsening of existing epilepsy the grades should be based on an increase from previous level of control to any of these levels.	NA	Increased frequency of pre-existing seizures (nonrepetitive) without change in seizure character OR Infrequent breakthrough seizures while on stable medication in a previously controlled seizure disorder	Change in seizure character from baseline either in duration or quality (e.g., severity or focality)	Seizures of any kind which are prolonged, repetitive (e.g., status epilepticus), or difficult to control (e.g., refractory epilepsy)
Seizure<br>**Pediatric < 18 years**	Seizure, generalized onset with or without secondary generalization, lasting < 5 minutes with < 24 hours postictal state	Seizure, generalized onset with or without secondary generalization, lasting 5 – 20 minutes with < 24 hours post ictal state	Seizure, generalized onset with or without secondary generalization, lasting > 20 minutes	Seizure, generalized onset with or without secondary generalization, requiring intubation and sedation
Syncope (not associated with a procedure)	NA	Present	NA	NA
Vertigo	Vertigo causing no or minimal interference with usual social & functional activities	Vertigo causing greater than minimal interference with usual social & functional activities	Vertigo causing inability to perform usual social & functional activities	Disabling vertigo causing inability to perform basic self-care functions

CLINICAL				
**PARAMETER**	**GRADE 1**<br>**MILD**	**GRADE 2**<br>**MODERATE**	**GRADE 3**<br>**SEVERE**	**GRADE 4**<br>**POTENTIALLY LIFE-THREATENING**
**RESPIRATORY**				
Bronchospasm (acute)	FEV1 or peak flow reduced to 70% – 80%	FEV1 or peak flow 50% – 69%	FEV1 or peak flow 25% – 49%	Cyanosis OR FEV1 or peak flow < 25% OR Intubation
Dyspnea or respiratory distress				
**Adult ≥ 14 years**	Dyspnea on exertion with no or minimal interference with usual social & functional activities	Dyspnea on exertion causing greater than minimal interference with usual social & functional activities	Dyspnea at rest causing inability to perform usual social & functional activities	Respiratory failure with ventilatory support indicated
**Pediatric < 14 years**	Wheezing OR minimal increase in respiratory rate for age	Nasal flaring OR Intercostal retractions OR Pulse oximetry 90% – 95%	Dyspnea at rest causing inability to perform usual social & functional activities OR Pulse oximetry < 90%	Respiratory failure with ventilatory support indicated
**MUSCULOSKELETAL**				
Arthralgia<br>See also Arthritis	Joint pain causing no or minimal interference with usual social & functional activities	Joint pain causing greater than minimal interference with usual social & functional activities	Joint pain causing inability to perform usual social & functional activities	Disabling joint pain causing inability to perform basic self-care functions
Arthritis<br>See also Arthralgia	Stiffness or joint swelling causing no or minimal interference with usual social & functional activities	Stiffness or joint swelling causing greater than minimal interference with usual social & functional activities	Stiffness or joint swelling causing inability to perform usual social & functional activities	Disabling joint stiffness or swelling causing inability to perform basic self-care functions
Bone Mineral Loss				
**Adult ≥ 21 years**	BMD t-score<br>-2.5 to -1.0	BMD t-score < -2.5	Pathological fracture (including loss of vertebral height)	Pathologic fracture causing life-threatening consequences
**Pediatric < 21 years**	BMD z-score<br>-2.5 to -1.0	BMD z-score < -2.5	Pathological fracture (including loss of vertebral height)	Pathologic fracture causing life-threatening consequences

CLINICAL				
**PARAMETER**	**GRADE 1**<br>**MILD**	**GRADE 2**<br>**MODERATE**	**GRADE 3**<br>**SEVERE**	**GRADE 4**<br>**POTENTIALLY LIFE-THREATENING**
Myalgia (noninjection site)	Muscle pain causing no or minimal interference with usual social & functional activities	Muscle pain causing greater than minimal interference with usual social & functional activities	Muscle pain causing inability to perform usual social & functional activities	Disabling muscle pain causing inability to perform basic self-care functions
Osteonecrosis	NA	Asymptomatic with radiographic findings AND No operative intervention indicated	Symptomatic bone pain with radiographic findings OR Operative intervention indicated	Disabling bone pain with radiographic findings causing inability to perform basic self-care functions
**GENITOURINARY**				
Cervicitis (symptoms)<br>(For use in studies evaluating topical study agents)<br>For other cervicitis see Infection: Infection (any other than HIV infection)	Symptoms causing no or minimal interference with usual social & functional activities	Symptoms causing greater than minimal interference with usual social & functional activities	Symptoms causing inability to perform usual social & functional activities	Symptoms causing inability to perform basic self-care functions
Cervicitis (clinical exam)<br>(For use in studies evaluating topical study agents)<br>For other cervicitis, see Infection: Infection (any other than HIV infection)	Minimal cervical abnormalities on examination (erythema, mucopurulent discharge, or friability) OR Epithelial disruption < 25% of total surface	Moderate cervical abnormalities on examination (erythema, mucopurulent discharge, or friability) OR Epithelial disruption of 25% – 49% total surface	Severe cervical abnormalities on examination (erythema, mucopurulent discharge, or friability) OR Epithelial disruption 50% – 75% total surface	Epithelial disruption > 75% total surface
Intermenstrual bleeding (IMB)	Spotting observed by participant OR Minimal blood observed during clinical or colposcopic examination	Inter-menstrual bleeding not greater in duration or amount than usual menstrual cycle	Inter-menstrual bleeding greater in duration or amount than usual menstrual cycle	Hemorrhage with lifethreatening hypotension OR Operative intervention indicated
Urinary tract obstruction (e.g., stone)	NA	Signs or symptoms of urinary tract obstruction without hydronephrosis or renal dysfunction	Signs or symptoms of urinary tract obstruction with hydronephrosis or renal dysfunction	Obstruction causing lifethreatening Consequences

CLINICAL				
**PARAMETER**	**GRADE 1**<br>**MILD**	**GRADE 2**<br>**MODERATE**	**GRADE 3**<br>**SEVERE**	**GRADE 4**<br>**POTENTIALLY LIFE-THREATENING**
Vulvovaginitis (symptoms)<br>(Use in studies evaluating topical study agents)<br>For other vulvovaginitis see Infection: Infection (any other than HIV infection)	Symptoms causing no or minimal interference with usual social & functional activities	Symptoms causing greater than minimal interference with usual social & functional activities	Symptoms causing inability to perform usual social & functional activities	Symptoms causing inability to perform basic self-care functions
Vulvovaginitis (clinical exam)<br>(Use in studies evaluating topical study agents)<br>For other vulvovaginitis see Infection: Infection (any other than HIV infection)	Minimal vaginal abnormalities on examination OR Epithelial disruption<br>< 25% of total surface	Moderate vaginal abnormalities on examination OR Epithelial disruption of 25% – 49% total surface	Severe vaginal abnormalities on examination OR Epithelial disruption 50% – 75% total surface	Vaginal perforation OR Epithelial disruption<br>> 75% total surface
**OCULAR/VISUAL**				
Uveitis	Asymptomatic but detectable on exam	Symptomatic anterior uveitis OR Medical intervention indicated	Posterior or pan-uveitis OR Operative intervention indicated	Disabling visual loss in affected eye(s)
Visual changes (from baseline)	Visual changes causing no or minimal interference with usual social & functional activities	Visual changes causing greater than minimal interference with usual social & functional activities	Visual changes causing inability to perform usual social & functional activities	Disabling visual loss in affected eye(s)
**ENDOCRINE/METABOLIC**				
Abnormal fat accumulation (e.g., back of neck, breasts, abdomen)	Detectable by study participant (or by caregiver for young children and disabled adults)	Detectable on physical exam by health care provider	Disfiguring OR Obvious changes on casual visual inspection	NA

CLINICAL				
**PARAMETER**	**GRADE 1**<br>**MILD**	**GRADE 2**<br>**MODERATE**	**GRADE 3**<br>**SEVERE**	**GRADE 4**<br>**POTENTIALLY LIFE-THREATENING**
Diabetes mellitus	NA	New onset without need to initiate medication OR Modification of current medications to regain glucose control	New onset with initiation of medication indicated OR Diabetes uncontrolled despite treatment modification	Life-threatening consequences (e.g., ketoacidosis, hyperosmolar nonketotic coma)
Gynecomastia	Detectable by study participant or caregiver (for young children and disabled adults)	Detectable on physical exam by health care provider	Disfiguring OR Obvious on casual visual inspection	NA
Hyperthyroidism	Asymptomatic	Symptomatic causing greater than minimal interference with usual social & functional activities OR Thyroid suppression therapy indicated	Symptoms causing inability to perform usual social & functional activities OR Uncontrolled despite treatment modification	Life-threatening consequences (e.g., thyroid storm)
Hypothyroidism	Asymptomatic	Symptomatic causing greater than minimal interference with usual social & functional activities OR Thyroid replacement therapy indicated	Symptoms causing inability to perform usual social & functional activities OR Uncontrolled despite treatment modification	Life-threatening consequences (e.g., myxedema coma)
Lipoatrophy (e.g., fat loss from the face, extremities, buttocks)	Detectable by study participant (or by caregiver for young children and disabled adults)	Detectable on physical exam by health care provider	Disfiguring OR Obvious on casual visual inspection	NA

LABORATORY				
**PARAMETER**	**GRADE 1**<br>**MILD**	**GRADE 2**<br>**MODERATE**	**GRADE 3**<br>**SEVERE**	**GRADE 4**<br>**POTENTIALLY LIFE-THREATENING**
**HEMATOLOGY** ***Standard International Units are listed in italics***				
Absolute CD4+ count<br>**Adult and Pediatric**<br>**> 13 years**<br>(HIV negative only)	300 – 400/mm³<br>*300 – 400/µL*	200 – 299/mm³<br>*200 – 299/µL*	100 – 199/mm³<br>*100 – 199/µL*	< 100/mm³<br>*< 100/µL*
Absolute lymphocyte count<br>**Adult and Pediatric**<br>**> 13 years**<br>(HIV negative only)	600 – 650/mm³<br>*0.600 x 10^9 –*<br>*0.650 x 10^9/L*	500 – 599/mm³<br>*0.500 x 10^9 –*<br>*0.599 x 10^9/L*	350 – 499/mm³<br>*0.350 x 10^9 –*<br>*0.499 x 10^9/L*	< 350/mm³<br>*< 0.350 x 10^9/L*
Absolute neutrophil count (ANC)				
**Adult and Pediatric**<br>**> 7 days**	1,000 – 1,300/mm³<br>*1.000 x 10^9 –*<br>*1.300 x 10^9/L*	750 – 999/mm³<br>*0.750 x 10^9 –*<br>*0.999 x 10^9/L*	500 – 749/mm³<br>*0.500 x 10^9 –*<br>*0.749 x 10^9/L*	< 500/mm³<br>*< 0.500 x 10^9/L*
**Infant**[a,b]<br>**2 – ≤ 7 days**	1,250 – 1,500/mm³<br>*1.250 x 10^9 –*<br>*1.500 x 10^9/L*	1,000 – 1,249/mm³<br>*1.000 x 10^9 –*<br>*1.249 x 10^9/L*	750 – 999/mm³<br>*0.750 x 10^9 –*<br>*0.999 x 10^9/L*	< 750/mm³<br>*< 0.750 x 10^9/L*
**Infant**[a,b] **1 day**	4,000 – 5,000/mm³<br>*4.000 x 10^9 –*<br>*5.000 x 10^9/L*	3,000 – 3,999/mm³<br>*3.000 x 10^9 –*<br>*3.999 x10^9/L*	1,500 – 2,999/mm³<br>*1.500 x 10^9 –*<br>*2.999 x 10^9/L*	< 1,500/mm³<br>*< 1.500 x 10^9/L*
Fibrinogen, decreased[d]	100 – 200 mg/dL<br>*1.00 – 2.00 g/L*<br>OR<br>≥ 0.75 to < 1.00 x LLN	75 – 99 mg/dL<br>*0.75 – 0.99 g/L*<br>OR<br>≥ 0.50 to < 0.75 x LLN	50 – 74 mg/dL<br>*0.50 – 0.74 g/L*<br>OR<br>≥ 0.25 to < 0.50 x LLN	< 50 mg/dL<br>*< 0.50 g/L*<br>OR<br>< 0.25 x LLN<br>OR Associated with gross bleeding
Hemoglobin (Hgb)[c]				
**Adult and Pediatric**<br>**≥ 57 days**<br>(HIV positive only)	8.5 – 10.0 g/dL<br>*5.2 – 6.1 mmol/L*	7.5 – 8.4 g/dL<br>*4.6 – 5.1 mmol/L*	6.5 – 7.4 g/dL<br>*3.9 – 4.5 mmol/L*	< 6.5 g/dL<br>*< 3.9 mmol/L*
**Adult and Pediatric**<br>**≥ 57 days**<br>(HIV negative only)	10.0 – 10.9 g/dL<br>*6.1 – 6.6 mmol/L*<br>OR<br>Any decrease<br>2.5 – 3.4 g/dL<br>*1.5 – 2.0 mmol/L*	9.0 – 9.9 g/dL<br>*5.5 – 6.0 mmol/L*<br>OR<br>Any decrease<br>3.5 – 4.4 g/dL<br>*2.1 – 2.6 mmol/L*	7.0 – 8.9 g/dL<br>*4.2 – 5.4 mmol/L*<br>OR<br>Any decrease<br>≥ 4.5 g/dL<br>*≥ 2.7 mmol/L*	< 7.0 g/dL<br>*< 4.2 mmol/L*
**Infant**[a,b]<br>**36 – 56 days**<br>(HIV positive or negative)	8.5 – 9.4 g/dL<br>*5.2 – 5.7 mmol/L*	7.0 – 8.4 g/dL<br>*4.2 – 5.1 mmol/L*	6.0 – 6.9 g/dL<br>*3.6 – 4.1 mmol/L*	< 6.0 g/dL<br>*< 3.6 mmol/L*

[a] Values are for term infants.

[b] Use age and sex appropriate values (e.g., bilirubin), including preterm infants.

[c] Revised; monomer conversion factor used for conversion from g/dL to mmol/L

[d] Revised.

LABORATORY				
**PARAMETER**	**GRADE 1**<br>**MILD**	**GRADE 2**<br>**MODERATE**	**GRADE 3**<br>**SEVERE**	**GRADE 4**<br>**POTENTIALLY LIFE-THREATENING**
**Infant**[a, b]<br>**22 – 35 days**<br>(HIV positive or negative)	9.5 – 10.5 g/dL<br>*5.8 – 6.4 mmol/L*	8.0 – 9.4 g/dL<br>*4.8 – 5.7 mmol/L*	7.0 – 7.9 g/dL<br>*4.2 – 4.7 mmol/L*	< 7.00 g/dL<br>*< 4.2 mmol/L*
**Infant**[a, b]<br>**1 – 21 days**<br>(HIV positive or negative)	12.0 – 13.0 g/dL<br>*7.3 – 7.9 mmol/L*	10.0 – 11.9 g/dL<br>*6.1 – 7.2 mmol/L*	9.0 – 9.9 g/dL<br>*5.5 – 6.0 mmol/L*	< 9.0 g/dL<br>*< 5.5 mmol/L*
International normalized ratio of prothrombin time (INR)[d]	≥ 1.1 to ≤ 1.5 x ULN	> 1.5 to ≤ 2.0 x ULN	> 2.0 to ≤ 3.0 x ULN	> 3.0 x ULN
Methemoglobin	5.0% – 10.0%	10.1% – 15.0%	15.1% – 20.0%	> 20.0%
Prothrombin time (PT)[c,d]	≥ 1.1 to ≤ 1.25 x ULN	> 1.25 to ≤ 1.50 x ULN	> 1.50 to ≤ 3.00 x ULN	> 3.00 x ULN
Partial thromboplastin time (PTT)[d]	≥ 1.1 to ≤ 1.66 x ULN	> 1.66 to ≤ 2.33 x ULN	> 2.33 to ≤ 3.00 x ULN	> 3.00 x ULN
Platelets, decreased	100,000 – 124,999/mm³<br>*100.000 x 10^9 – 124.999 x 10^9/L*	50,000 – 99,999/mm³<br>*50.000 x 10^9– 99.999 x 10^9/L*	25,000 – 49,999/mm³<br>*25.000 x 10^9– 49.999 x 10^9/L*	< 25,000/mm³<br>*< 25.000 x 10^9/L*
WBC, decreased	2,000 – 2,500/mm³<br>*2.000 x 10^9 – 2.500 x 10^9/L*	1,500 – 1,999/mm³<br>*1.500 x 10^9– 1.999 x 10^9/L*	1,000 – 1,499/mm³<br>*1.000 x 10^9– 1.499 x 10^9/L*	< 1,000/mm³<br>*< 1.000 x 10^9/L*
**CHEMISTRIES** ***Standard International Units are listed in italics***				
Acidosis	NA	pH < normal, but ≥ 7.3	pH < 7.3 without life-threatening consequences	pH < 7.3 with life-threatening consequences
Albumin, serum, low	3.0 g/dL – < LLN<br>*30 g/L – < LLN*	2.0 – 2.9 g/dL<br>*20 – 29 g/L*	< 2.0 g/dL<br>*< 20 g/L*	NA
Alkaline phosphatase[d]	≥ 1.25 to ≤ 2.5 x ULN[b]	> 2.5 to ≤ 5.0 x ULN[b]	> 5.0 to ≤ 10.0 x ULN[b]	> 10.0 x ULN[b]
Alkalosis	NA	pH > normal, but ≤ 7.5	pH > 7.5 without lifethreatening consequences	pH > 7.5 with lifethreatening consequences
ALT (SGPT)[d]	≥ 1.25 to ≤ 2.5 x ULN	> 2.5 to ≤ 5.0 x ULN	> 5.0 to ≤ 10.0 x ULN	> 10.0 x ULN
AST (SGOT)[d]	≥ 1.25 to ≤ 2.5 x ULN	> 2.5 to ≤ 5.0 x ULN	> 5.0 to ≤ 10.0 x ULN	> 10.0 x ULN
Bicarbonate, serum, low	16.0 mEq/L – < LLN<br>*16.0 mmol/L – < LLN*	11.0 – 15.9 mEq/L<br>*11.0 – 15.9 mmol/L*	8.0 – 10.9 mEq/L<br>*8.0 – 10.9 mmol/L*	< 8.0 mEq/L<br>*< 8.0 mmol/L*

[a] Values are for term infants.

[b] Use age- and sex-appropriate values (e.g., bilirubin), including preterm infants.

[c] If the local laboratory is reporting PT as percentage, only INR value will be considered for reporting PT related abnormalities and adverse events.

[d] Revised.

LABORATORY				
**PARAMETER**	**GRADE 1**<br>**MILD**	**GRADE 2**<br>**MODERATE**	**GRADE 3**<br>**SEVERE**	**GRADE 4**<br>**POTENTIALLY LIFE-THREATENING**
Bilirubin (Total)[c]				
**Adult and Pediatric >14 days**	≥ 1.1 to ≤ 1.5 x ULN	> 1.5 to ≤ 2.5 x ULN	> 2.5 to ≤ 5.0 x ULN	> 5.0 x ULN
**Infant[a, b] ≤ 14 days** (non-hemolytic)	NA	20.0 – 25.0 mg/dL<br>*342 – 428 µmol/L*	25.1 – 30.0 mg/dL<br>*429 – 513 µmol/L*	> 30.0 mg/dL<br>*> 513.0 µmol/L*
**Infant[a, b] ≤ 14 days** (hemolytic)	NA	NA	20.0 – 25.0 mg/dL<br>*342 – 428 µmol/L*	> 25.0 mg/dL<br>*> 428 µmol/L*
Calcium, serum, high (corrected for albumin)				
**Adult and Pediatric ≥ 7 days**	10.6 – 11.5 mg/dL<br>*2.65 – 2.88 mmol/L*	11.6 – 12.5 mg/dL<br>*2.89 – 3.13 mmol/L*	12.6 – 13.5 mg/dL<br>*3.14 – 3.38 mmol/L*	> 13.5 mg/dL<br>*> 3.38 mmol/L*
**Infant[a, b] < 7 days**	11.5 – 12.4 mg/dL<br>*2.88 – 3.10 mmol/L*	12.5 – 12.9 mg/dL<br>*3.11 – 3.23 mmol/L*	13.0 – 13.5 mg/dL<br>*3.24 – 3.38 mmol/L*	> 13.5 mg/dL<br>*> 3.38 mmol/L*
Calcium, serum, low (corrected for albumin)				
**Adult and Pediatric ≥ 7 days**	7.8 – 8.4 mg/dL<br>*1.95 – 2.10 mmol/L*	7.0 – 7.7 mg/dL<br>*1.75 – 1.94 mmol/L*	6.1 – 6.9 mg/dL<br>*1.53 – 1.74 mmol/L*	< 6.1 mg/dL<br>*< 1.53 mmol/L*
**Infant[a, b] < 7 days**	6.5 – 7.5 mg/dL<br>*1.63 – 1.88 mmol/L*	6.0 – 6.4 mg/dL<br>*1.50 – 1.62 mmol/L*	5.50 – 5.90 mg/dL<br>*1.38 – 1.49 mmol/L*	< 5.50 mg/dL<br>*< 1.38 mmol/L*
Cardiac troponin I (cTnI)	NA	NA	NA	Levels consistent with myocardial infarction or unstable angina as defined by the manufacturer
Cardiac troponin T (cTnT)	NA	NA	NA	≥ 0.20 ng/ mL OR<br>Levels consistent with myocardial infarction or unstable angina as defined by the manufacturer
Cholesterol (fasting)				
**Adult ≥ 18 years**	200 – 239 mg/dL<br>*5.18 – 6.19 mmol/L*	240 – 300 mg/dL<br>*6.20 – 7.77 mmol/L*	> 300 mg/dL<br>*> 7.77 mmol/L*	NA
**Pediatric < 18 years**	170 – 199 mg/dL<br>*4.40 – 5.15 mmol/L*	200 – 300 mg/dL<br>*5.16 – 7.77 mmol/L*	> 300 mg/dL<br>*> 7.77 mmol/L*	NA
Creatine kinase[c]	≥ 3.0 to ≤ 5.9 x ULN[b]	> 5.9 to ≤ 9.9 x ULN[b]	> 9.9 to ≤ 19.9 x ULN[b]	> 19.9 x ULN[b]
Creatinine[c]	≥ 1.1 to ≤ 1.3 x ULN[b]	> 1.3 to ≤ 1.8 x ULN[b]	> 1.8 to ≤ 3.4 x ULN[b]	> 3.4 x ULN[b]
Glucose, serum, high				
Nonfasting	116 – 160 mg/dL<br>*6.44 – 8.88 mmol/L*	161 – 250 mg/dL<br>*8.89 – 13.88 mmol/L*	251 – 500 mg/dL<br>*13.89 – 27.75 mmol/L*	> 500 mg/dL<br>*> 27.75 mmol/L*
Fasting	110 – 125 mg/dL<br>*6.11 – 6.94 mmol/L*	126 – 250 mg/dL<br>*6.95 – 13.88 mmol/L*	251 – 500 mg/dL<br>*13.89 – 27.75 mmol/L*	> 500 mg/dL<br>*> 27.75 mmol/L*

[a] Values are for term infants.

[b] Use age- and sex-appropriate values (e.g., bilirubin), including preterm infants.

[c] Revised.

LABORATORY				
**PARAMETER**	**GRADE 1**<br>**MILD**	**GRADE 2**<br>**MODERATE**	**GRADE 3**<br>**SEVERE**	**GRADE 4**<br>**POTENTIALLY LIFE-THREATENING**
Glucose, serum, low				
**Adult and Pediatric ≥ 1 month**	55 – 64 mg/dL<br>*3.05 – 3.55 mmol/L*	40 – 54 mg/dL<br>*2.22 – 3.00 mmol/L*	30 – 39 mg/dL<br>*1.67 – 2.21 mmol/L*	< 30 mg/dL<br>*< 1.67 mmol/L*
**Infant[a,b] < 1 month**	50 – 54 mg/dL<br>*2.78 – 3.00 mmol/L*	40 – 49 mg/dL<br>*2.22 – 2.77 mmol/L*	30 – 39 mg/dL<br>*1.67 – 2.21 mmol/L*	< 30 mg/dL<br>*< 1.67 mmol/L*
Lactate	< 2.0 x ULN without acidosis	≥ 2.0 x ULN without acidosis	Increased lactate with pH < 7.3 without life-threatening consequences	Increased lactate with pH < 7.3 with life-threatening consequences
LDL cholesterol (fasting)				
**Adult ≥ 18 years**	130 – 159 mg/dL<br>*3.37 – 4.12 mmol/L*	160 – 190 mg/dL<br>*4.13 – 4.90 mmol/L*	≥ 191 mg/dL<br>*≥ 4.91 mmol/L*	NA
**Pediatric > 2 – < 18 Years**	110 – 129 mg/dL<br>*2.85 – 3.34 mmol/L*	130 – 189 mg/dL<br>*3.35 – 4.90 mmol/L*	≥ 190 mg/dL<br>*≥ 4.91 mmol/L*	NA
Lipase[c]	≥ 1.1 to ≤ 1.5 x ULN	> 1.5 to ≤ 3.0 x ULN	> 3.0 to ≤ 5.0 x ULN	> 5.0 x ULN
Magnesium, serum, low	1.2 – 1.4 mEq/L<br>*0.60 – 0.70 mmol/L*	0.9 – 1.1 mEq/L<br>*0.45 – 0.59 mmol/L*	0.6 – 0.8 mEq/L<br>*0.30 – 0.44 mmol/L*	< 0.60 mEq/L<br>*< 0.30 mmol/L*
Pancreatic amylase[c]	≥ 1.1 to ≤ 1.5 x ULN	> 1.5 to ≤ 2.0 x ULN	> 2.0 to ≤ 5.0 x ULN	> 5.0 x ULN
Phosphate, serum, low				
**Adult and Pediatric > 14 years**	2.5 mg/dL – < LLN<br>*0.81 mmol/L – < LLN*	2.0 – 2.4 mg/dL<br>*0.65 – 0.80 mmol/L*	1.0 – 1.9 mg/dL<br>*0.32 – 0.64 mmol/L*	< 1.00 mg/dL<br>*< 0.32 mmol/L*
**Pediatric 1 – 14 years**	3.0 – 3.5 mg/dL<br>*0.97 – 1.13 mmol/L*	2.5 – 2.9 mg/dL<br>*0.81 – 0.96 mmol/L*	1.5 – 2.4 mg/dL<br>*0.48 – 0.80 mmol/L*	< 1.50 mg/dL<br>*< 0.48 mmol/L*
**Pediatric < 1 year**	3.5 – 4.5 mg/dL<br>*1.13 – 1.45 mmol/L*	2.5 – 3.4 mg/dL<br>*0.81 – 1.12 mmol/L*	1.5 – 2.4 mg/dL<br>*0.48 – 0.80 mmol/L*	< 1.50 mg/dL<br>*< 0.48 mmol/L*
Potassium, serum, high	5.6 – 6.0 mEq/L<br>*5.6 – 6.0 mmol/L*	6.1 – 6.5 mEq/L<br>*6.1 – 6.5 mmol/L*	6.6 – 7.0 mEq/L<br>*6.6 – 7.0 mmol/L*	> 7.0 mEq/L<br>*> 7.0 mmol/L*
Potassium, serum, low	3.0 – 3.4 mEq/L<br>*3.0 – 3.4 mmol/L*	2.5 – 2.9 mEq/L<br>*2.5 – 2.9 mmol/L*	2.0 – 2.4 mEq/L<br>*2.0 – 2.4 mmol/L*	< 2.0 mEq/L<br>*< 2.0 mmol/L*
Sodium, serum, high	146 – 150 mEq/L<br>*146 – 150 mmol/L*	151 – 154 mEq/L<br>*151 – 154 mmol/L*	155 – 159 mEq/L<br>*155 – 159 mmol/L*	≥ 160 mEq/L<br>*≥ 160 mmol/L*
Sodium, serum, low	130 – 135 mEq/L<br>*130 – 135 mmol/L*	125 – 129 mEq/L<br>*125 – 129 mmol/L*	121 – 124 mEq/L<br>*121 – 124 mmol/L*	≤ 120 mEq/L<br>*≤ 120 mmol/L*
Triglycerides (fasting)	NA	500 – 750 mg/dL<br>*5.65 – 8.48 mmol/L*	751 – 1,200 mg/dL<br>*8.49 – 13.56 mmol/L*	> 1,200 mg/dL<br>*> 13.56 mmol/L*
Uric acid	7.5 – 10.0 mg/dL<br>*0.45 – 0.59 mmol/L*	10.1 – 12.0 mg/dL<br>*0.60 – 0.71 mmol/L*	12.1 – 15.0 mg/dL<br>*0.72 – 0.89 mmol/L*	> 15.0 mg/dL<br>*> 0.89 mmol/L*

[a] Values are for term infants.

[b] Use age- and sex-appropriate values (e.g., bilirubin), including preterm infants.

[c] Revised.

LABORATORY				
**PARAMETER**	**GRADE 1**<br>**MILD**	**GRADE 2**<br>**MODERATE**	**GRADE 3**<br>**SEVERE**	**GRADE 4**<br>**POTENTIALLY LIFE-THREATENING**
**URINALYSIS** ***Standard International Units are listed in italics***				
Hematuria (microscopic)	6 – 10 RBC/HPF	> 10 RBC/HPF	Gross, with or without clots OR with RBC casts	Transfusion indicated
Proteinuria, random collection	1 +	2 – 3 +	4 +	NA
Proteinuria, 24 hour collection				
**Adult and Pediatric ≥ 10 years**	200 – 999 mg/24 h<br>*0.200 – 0.999 g/d*	1,000 – 1,999 mg/24 h<br>*1.000 – 1.999 g/d*	2,000 – 3,500 mg/24 h<br>*2.000 – 3.500 g/d*	> 3,500 mg/24 h<br>*> 3.500 g/d*
**Pediatric > 3 months – < 10 years**	201 – 499 mg/m2/24 h<br>*0.201 – 0.499 g/d*	500 – 799 mg/m2/24 h<br>*0.500 – 0.799 g/d*	800– 1,000 mg/m2/24 h<br>*0.800 – 1.000 g/d*	> 1,000 mg/m2/24 h<br>*> 1.000 sg/d*

Appendix III: List of contraindicated medication

- Agents suspected to cause **pancreatitis** (potential additive/synergist toxicity):

    e.g. azathioprine

    6-mercaptopurine

    furosemide

    pentamidine

    sulfonamides

    tetracycline

    thiazide diuretics

    valproic acid

- Agents suspected to cause **neuropathy** (potential additive/synergist toxicity):

    - Heart or blood pressure medications:

        amiodarone,

        hydralazine

        perhexiline

    - Drugs used to fight cancer:

        vincristine

        cisplatin

    - Drugs used to fight infections:

        metronidazole (Flagyl)

        nitrofurantoin,

        thalidomide (used to fight leprosy)

        INH (isoniazid) -- used against tuberculosis

    - dapsone

    - Anticonvulsants:

        phenytoin

    - Anti-alcohol drugs:

        disulfiram

    - Drugs to fight HIV:

        zidovudine (Retrovir, formerly AZT)

        didanosine (Videx)

        stavudine (Zerit)

        zalcitabine (Hivid)

        ritonavir (Norvir)

amprenavir (Agenerase)

- Drugs to lower cholesterol:

lovastatin (Mevacor)
indapamid (Lozol)
gemfibrozil (Lopid)

- All **antacids** (Unknown effect upon enteric coat release characteristics). The use of oral antacids is not allowed within 12 hours prior to and during PK sampling

- All **histamine-2 blockers** (Increased gastric pH): H2 blockers are not allowed within 24 hours prior to and during PK sampling:

  e.g. cimetidine
  famotidine
  nizatidine
  ranitidine

- All **Proton pump inhibitors** (Increased gastric pH):

  e.g. lansoprazole
  omeprazole
  pantoprazole
  rabeprazole
  esomeprazole

- Dugs effecting **gastric** emptying time (delayed ddI release from enteric coating):

  e.g. metoclopramide
  tegaserod
  erythromycin
  domperidone

- All **antineoplastics** (potential additive/synergist toxicity):

  e.g. cyclophosphamide (Cytoxan)
  doxorubicin (Adriamycin)
  finasteride (Propecia; Proscar)
  paclitaxel (Taxol)
  tamoxifen (Nolvadex)

- All **immunosuppressive agents** (potential additive/synergist toxicity):

  e.g. azathioprine (Imuran)
  cyclosporin (Sandimmune, Neoral)
  glatiramer acetate (Copaxone)
  mycophenolate (CellCept)
  sirolimus (Rapamune)

- All **other anti-HIV medications** (additive antiviral effects and drug interactions):

  abacavir + lamivudine + zidovudine (Ziagen + Epivir + Retrovir (Trizivir + Epzicom))
  amprenavir (Agenerase)
  atazanavir (Reyataz)
  crixivan (Indinavir)
  delavirdine (Rescriptor)
  darunavir (Prezista)
  efavirenz (Sustiva / Stocrin)
  elvucitabine
  emtricitabine + efavirenz+ tenofovir (Emtriva + Sustiva + Viread (Atripla))
  emtricitabine, FTC (Emtriva)
  enfuvirtide, T20 (Fuzeon)
  lamivudine + zidovudine (Epivir + Retrovir (Combivir))
  fosamprenavir (Lexiva)
  lamivudine, 3TC (Epivir)
  lopinavir + ritonavir (Kaletra)
  maraviroc (Celsentri (Selzentry))
  nelfinavir (Viracept)
  nevirapine (Viramune)
  raltegravir (Isentress)
  ritonavir (Norvir)
  saquinavir (Invirase)
  stavudine + d4T (Zerit)
  tenofovir (Viread)
  TMC-125 (Etravirine)
  tipranavir (Aptivus)
  tenofovir + emtricitabine (Viread + Emtriva (Truvada))
  zidovudine, AZT (Retrovir)

- **entecavir** (additive antiviral effects)

- **adefovir** (additive antiviral effects and potential additive/synergist toxicity)
- **ribavirin** (increased didanosine plasma concentrations/increased ddATP)
- **ganciclovir/valganciclovir** (increased didanosine plasma concentrations)
- **methadone** (decreased didanosine plasma concentrations)
- **allopurinol** (increased didanosine plasma concentrations)
