# Supplementary material for: VS411 Reduced Immune Activation and HIV-1 RNA Levels in 28 Days: Randomized Proof-of-Concept Study for AntiViral-HyperActivation Limiting Therapeutics
Source: PLoS One. 2012 Oct 19;7(10):e47485. doi: 10.1371/journal.pone.0047485 (PMC3477169; doi:10.1371/journal.pone.0047485)
Supplement: Sub-Study Demographics S1 — Sub-Study Demographics Table. (DOCX) [file pone.0047485.s003.docx]

|  |  |  |  |  |  |  |  |  |  |  |
| --- | --- | --- | --- | --- | --- | --- | --- | --- | --- | --- |

Sub-study Table 1. Biomarker Sub-study Baseline Demographics (n = 38) Versus mITT* (n = 58).

| **Subject number** | **Investigator Site** | **Treatment Arm** | **Baseline**CD4^+^a** | **Baseline HIV RNAb** | **Gender** | **Age (years)** | **Race** | **Height (cm)** | **Weight (kg)** | **BMI (kg/m2)** |
| --- | --- | --- | --- | --- | --- | --- | --- | --- | --- | --- |
| 302 | Italy - Site 1 | 600/400 | 347.0 | 4.21 | Male | 43 | Caucasian | 166.0 | 77.0 | 27.9 |
| 303 | Italy - Site 1 | 600/200 | 352.5 | 4.16 | Female | 31 | Caucasian | 163.0 | 90.0 | 33.9 |
| 304 | Italy - Site 1 | 300/200 | 383.5 | 4.21 | Male | 26 | Caucasian | 171.0 | 80.0 | 27.4 |
| 702 | Italy - Site 1 | 300/400 | 321.5 | 4.45 | Male | 30 | Caucasian | 174.0 | 77.0 | 25.4 |
| 305 | Italy - Site 2 | 900/200 | 567.5 | 4.29 | Male | 33 | Caucasian | 163.0 | 63.6 | 23.9 |
| 701 | Italy - Site 2 | 600/200 | 383.5 | 4.34 | Male | 30 | Black | 168.0 | 68.1 | 24.1 |
| 203 | Argentina- Site 1 | 300/200 | 529.0 | 4.01 | Male | 25 | Hispanic | 180.0 | 75.0 | 23.1 |
| 204 | Argentina- Site 1 | 300/400 | 704.0 | 4.95 | Male | 33 | Hispanic | 175.0 | 75.8 | 24.8 |
| 208 | Argentina- Site 1 | 600/200 | 966.0 | 3.62 | Female | 25 | Caucasian | 155.0 | 60.5 | 25.2 |
| 402 | Argentina- Site 1 | 300/200 | 511.0 | 4.18 | Female | 40 | Caucasian | 171.0 | 86.0 | 29.4 |
| 2 | Uganda | 600/400 | 470.5 | 3.97 | Female | 33 | Black | 166.0 | 60.0 | 21.8 |
| 3 | Uganda | 300/200 | 424.5 | 5.08 | Female | 24 | Black | 148.0 | 70.0 | 32.0 |
| 4 | Uganda | 900/200 | 823.0 | 3.93 | Male | 36 | Black | 162.0 | 68.5 | 26.1 |
| 5 | Uganda | 600/200 | 265.5 | 4.70 | Male | 35 | Black | 167.0 | 60.0 | 21.5 |
| 6 | Uganda | 600/400 | 262.0 | 4.66 | Female | 32 | Black | 162.0 | 76.0 | 29.0 |
| 7 | Uganda | 300/400 | 377.5 | missing | Female | 27 | Black | 164.0 | 79.5 | 29.6 |
| 8 | Uganda | 600/200 | 756.5 | missing | Female | 30 | Black | 157.0 | 62.5 | 25.4 |
| 9 | Uganda | 900/200 | 373.0 | 4.56 | Female | 30 | Black | 159.0 | 90.0 | 35.6 |
| 11 | Uganda | 300/200 | 540.0 | 4.84 | Female | 44 | Black | 172.0 | 69.0 | 23.3 |
| 10 | Uganda | 300/200 | 382.5 | 3.54 | Female | 39 | Black | 156.0 | 68.0 | 27.9 |
| 12 | Uganda | 600/200 | 724.0 | 4.00 | Female | 24 | Black | 168.0 | 66.0 | 23.4 |
| 15 | Uganda | 300/400 | 303.5 | 3.99 | Male | 21 | Black | 166.0 | 64.0 | 23.2 |
| 16 | Uganda | 900/200 | 269.0 | 4.74 | Male | 34 | Black | 182.0 | 82.0 | 24.8 |
| 17 | Uganda | 300/200 | 262.0 | 3.67 | Female | 40 | Black | 157.0 | 99.0 | 40.2 |
| 21 | Uganda | 300/400 | 396.0 | 3.35 | Female | 24 | Black | 159.0 | 60.0 | 23.7 |
| 22 | Uganda | 900/200 | 551.5 | 3.75 | Female | 43 | Black | 170.0 | 89.0 | 30.8 |
| 26 | Uganda | 900/200 | 501.5 | 4.33 | Female | 26 | Black | 161.0 | 65.0 | 25.1 |
| 24 | Uganda | 300/200 | 510.0 | 4.88 | Male | 33 | Black | 174.0 | 68.0 | 22.5 |
| 30 | Uganda | 300/400 | 357.0 | 4.96 | Female | 32 | Black | 160.0 | 60.0 | 23.4 |
| 28 | Uganda | 600/400 | 280.5 | 3.91 | Female | 51 | Black | 162.0 | 75.0 | 28.6 |
| 31 | Uganda | 600/400 | 511.5 | 4.61 | Male | 40 | Black | 169.0 | 70.0 | 24.5 |
| 32 | Uganda | 300/400 | 242.5 | 4.55 | Male | 33 | Black | 170.0 | 60.0 | 20.8 |
| **Cohort [n=38], mdn**^c^ |  |  | **389.8** | **4.24** |  | **32.5** |  | **166.0** | **69.5** | **25.2** |
| **mITT* [n=58], mdn** |  |  | **407.0** | **4.62** |  | **33.0** |  | **166.0** | **70.3** | **25.1** |

* Modified Intent To Treat ** Average of Visit 1 (screening) and Visit 2 (baseline) *** mdn = median a cells/mm3 b log10copies/mL

Sub-study Table 2. HIV-1 Specific Immune Response Sub-study Baseline Demographics (n = 22) Versus mITT *(n = 58).

| **Subject number** | **Investigator Site** | **Treatment Arm** | **Baseline **CD4^+^a** | **Baseline HIV RNAb** | **Gender** | **Age (years)** | **Race** | **Height (cm)** | **Weight (kg)** | **BMI (kg/m2)c** |
| --- | --- | --- | --- | --- | --- | --- | --- | --- | --- | --- |
| 302 | Italy - Site 1 | 600/400 | 347.0 | 4.21 | Male | **43** | Caucasian | 166 | 77.0 | 27.9 |
| 303 | Italy - Site 1 | 600/200 | 352.5 | 4.16 | Female | **31** | Caucasian | 163 | 90.0 | 33.9 |
| 304 | Italy - Site 1 | 300/200 | 383.5 | 4.21 | Male | **26** | Caucasian | 171 | 80.0 | 27.4 |
| 702 | Italy - Site 1 | 300/400 | 321.5 | 4.45 | Male | **30** | Caucasian | 174 | 77.0 | 25.4 |
| 305 | Italy - Site 2 | 900/200 | 567.5 | 4.29 | Male | **33** | Caucasian | 163 | 63.6 | 23.9 |
| 701 | Italy - Site 2 | 600/200 | 383.5 | 4.34 | Male | **30** | Black | 168 | 68.1 | 24.1 |
| 203 | Argentina - Site 1 | 300/200 | 529.0 | 4.01 | Male | **25** | Hispanic | 180 | 75.0 | 23.1 |
| 204 | Argentina - Site 1 | 300/400 | 704.0 | 4.95 | Male | **33** | Hispanic | 175. | 75.8 | 24.8 |
| 208 | Argentina - Site 1 | 600/200 | 966.0 | 3.62 | Female | **25** | Caucasian | 155 | 60.5 | 25.2 |
| 402 | Argentina - Site 1 | 300/200 | 511.0 | 4.18 | Female | **40** | Caucasian | 171 | 86.0 | 29.4 |
| 2 | Uganda | 600/400 | 470.5 | 3.97 | Female | **33** | Black | 166 | 60.0 | 21.8 |
| 3 | Uganda | 300/200 | 424.5 | 5.08 | Female | **24** | Black | 148 | 70.0 | 32.0 |
| 4 | Uganda | 900/200 | 823.0 | 3.93 | Male | **36** | Black | 162 | 68.5 | 26.1 |
| 7 | Uganda | 300/400 | 377.5 | missing | Female | **27** | Black | 164 | 79.5 | 29.6 |
| 8 | Uganda | 600/200 | 756.5 | missing | Female | **30** | Black | 157 | 62.5 | 25.4 |
| 9 | Uganda | 900/200 | 373.0 | 4.56 | Female | **30** | Black | 159 | 90.0 | 35.6 |
| 11 | Uganda | 300/200 | 540.0 | 4.84 | Female | **44** | Black | 172 | 69.0 | 23.3 |
| 16 | Uganda | 900/200 | 269.0 | 4.74 | Male | **34** | Black | 182 | 82.0 | 24.8 |
| 21 | Uganda | 300/400 | 396.0 | 3.35 | Female | **24** | Black | 159 | 60.0 | 23.7 |
| 22 | Uganda | 900/200 | 551.5 | 3.75 | Female | **43** | Black | 170 | 89.0 | 30.8 |
| 26 | Uganda | 900/200 | 501.5 | 4.33 | Female | **26** | Black | 161 | 65.0 | 25.1 |
| 24 | Uganda | 300/200 | 510.0 | 4.88 | Male | **33** | Black | 174 | 68.0 | 22.5 |
| **Cohort [n=22], mdn**^c^ |  |  | **486.0** | **4.25** |  | **30.5** |  | **166.0** | **72.5** | **25.3** |
| **mITT* [n=58], mdn** |  |  | **407.0** | **4.62** |  | **33.0** |  | **166.0** | **70.3** | **25.1** |

* Modified Intent To Treat ** Average of Visit 1 (screening) and Visit 2 (baseline) *** mdn = median a cells/mm3 b log10copies/mL
